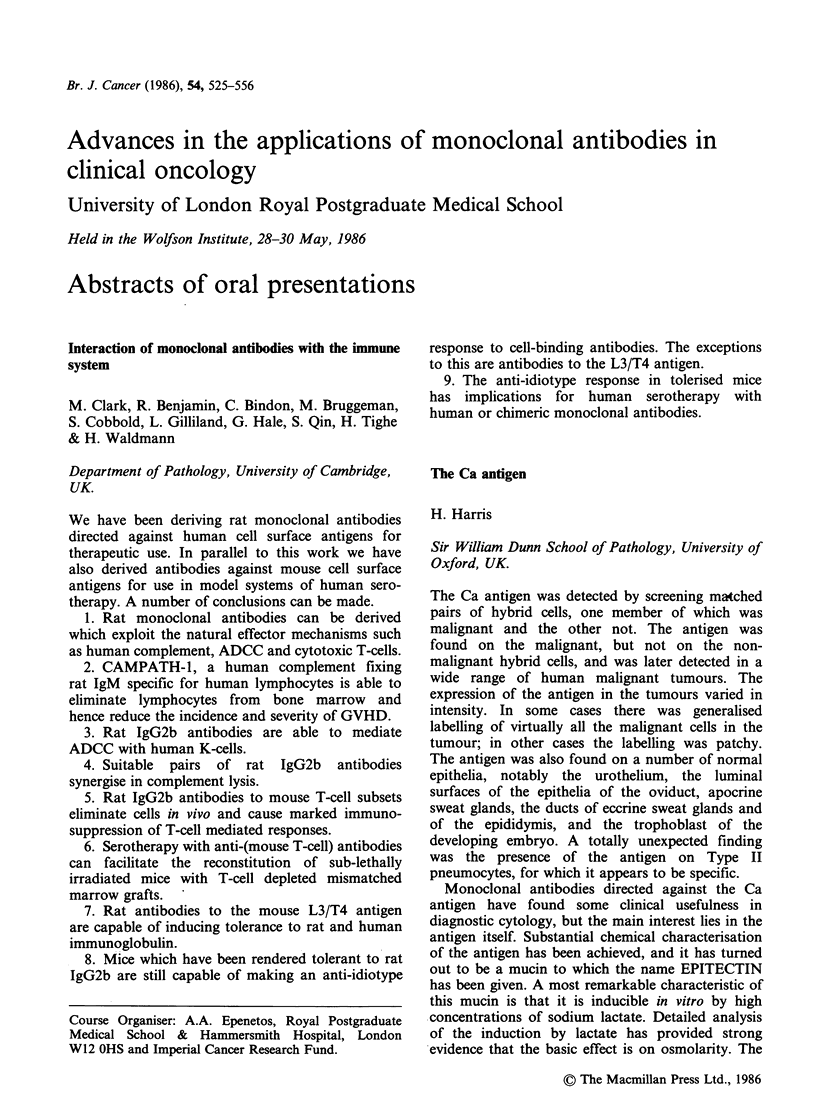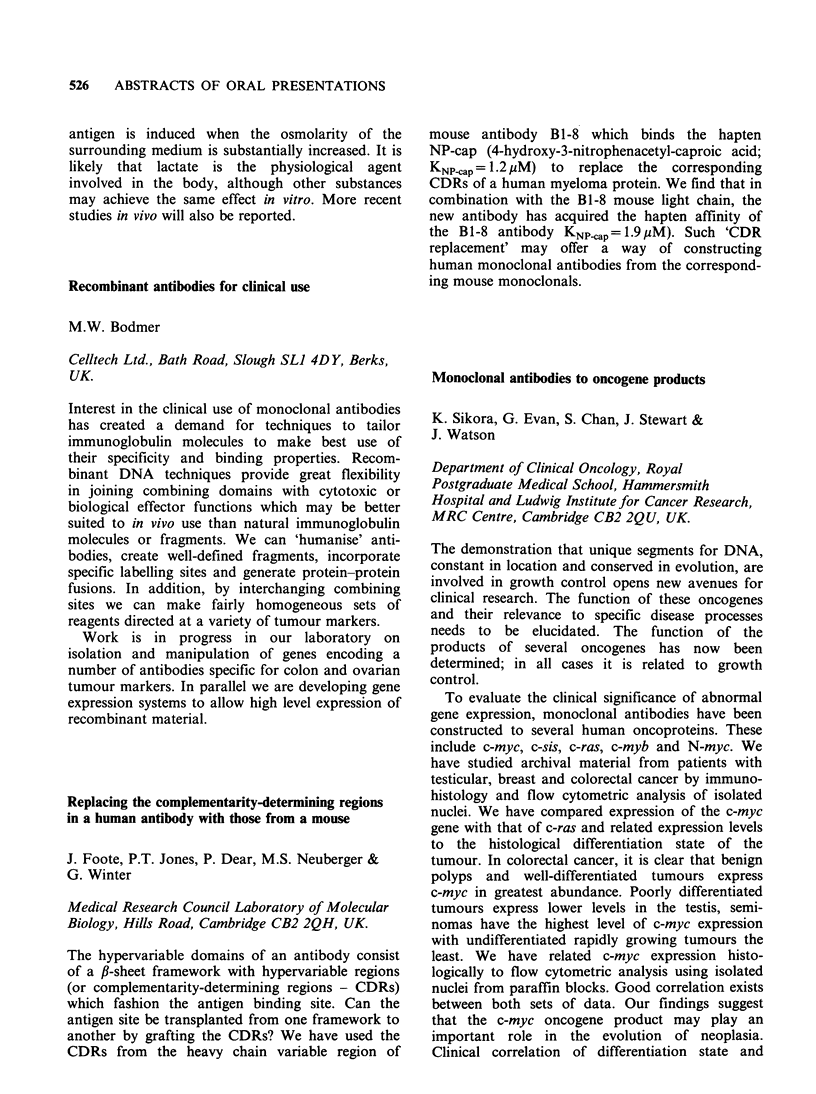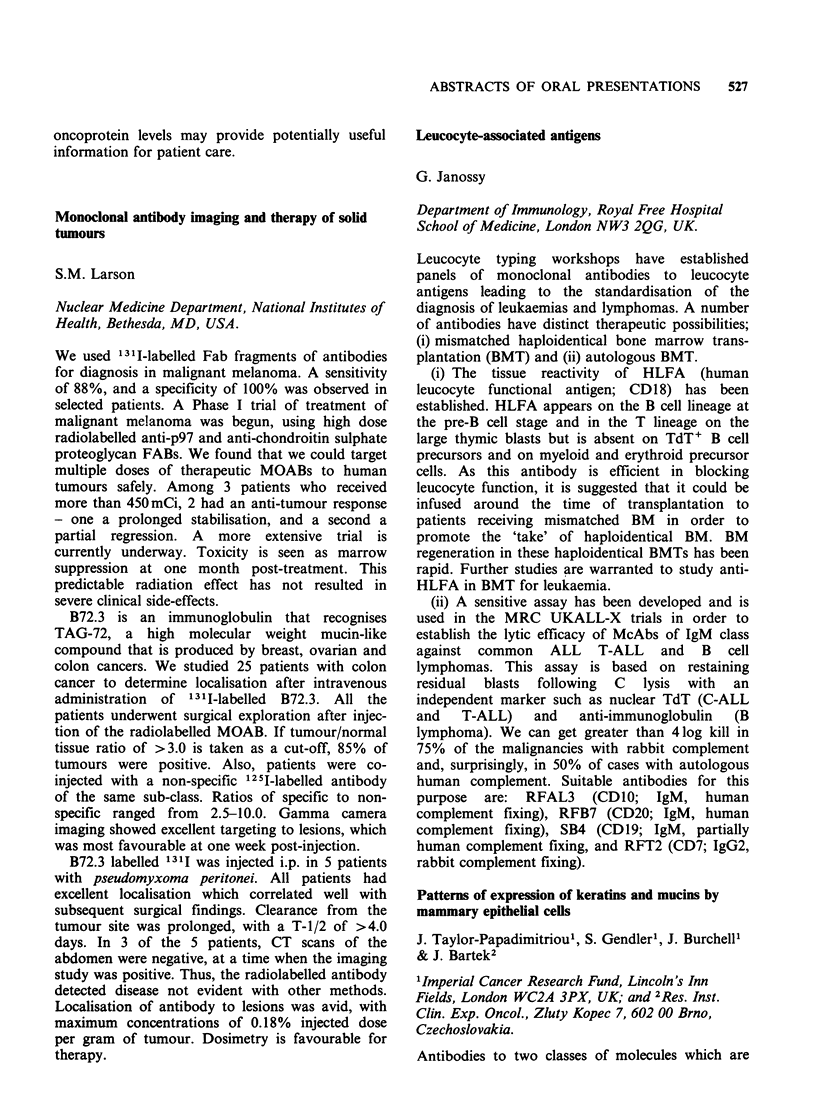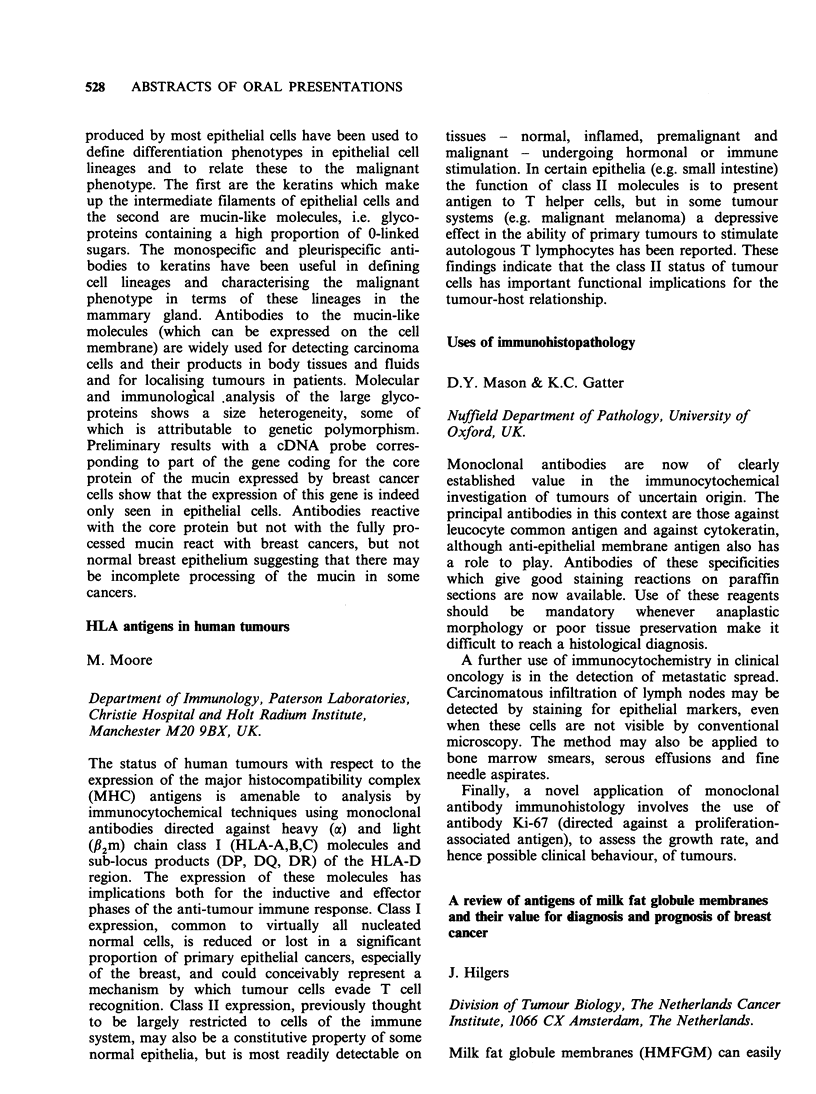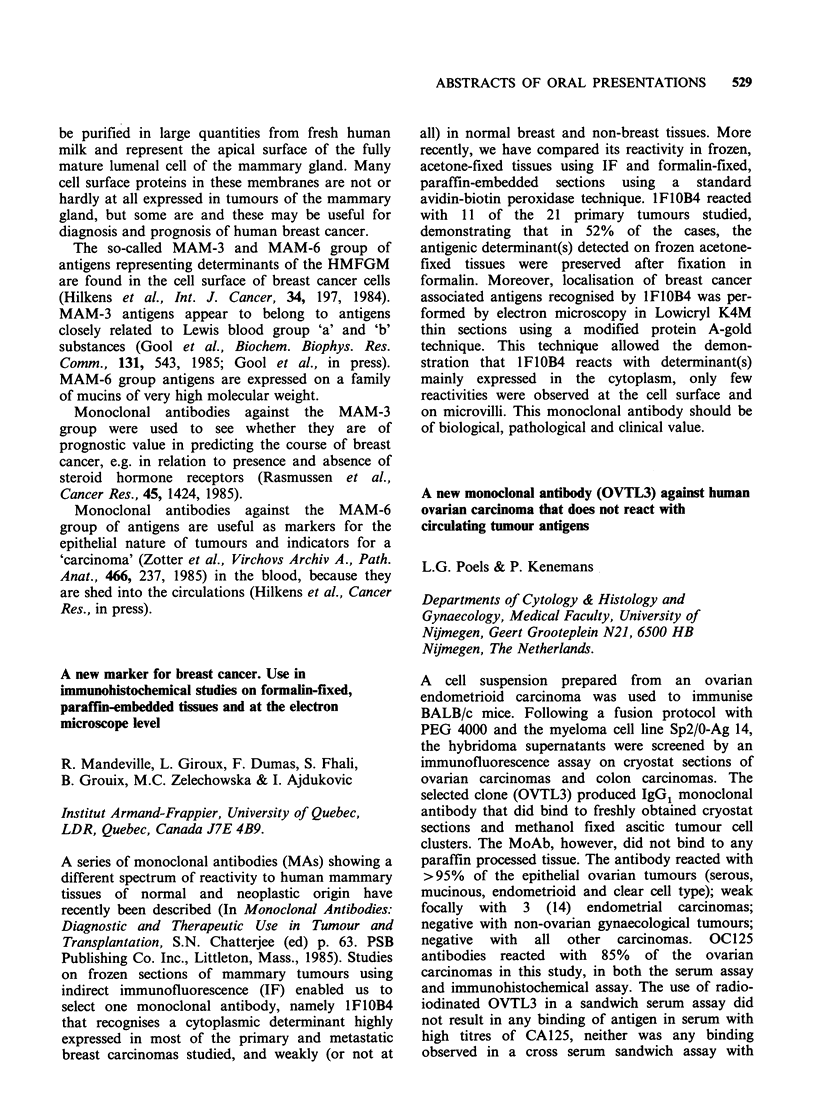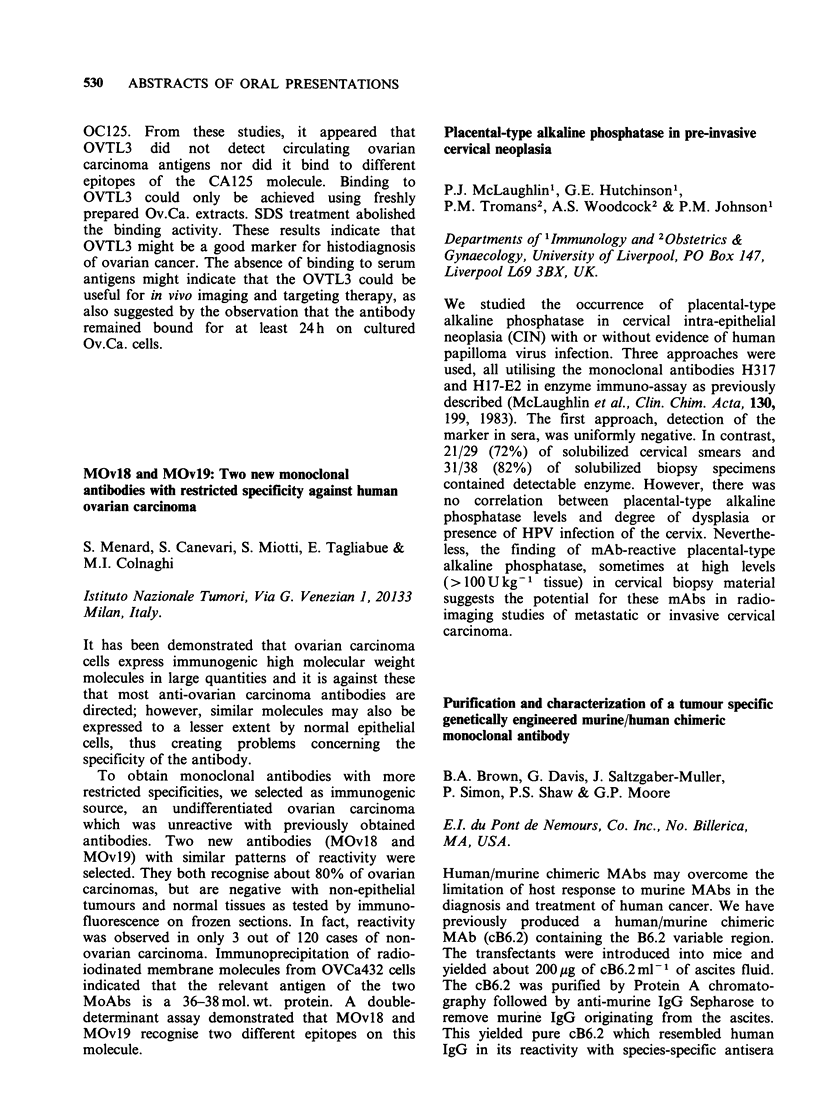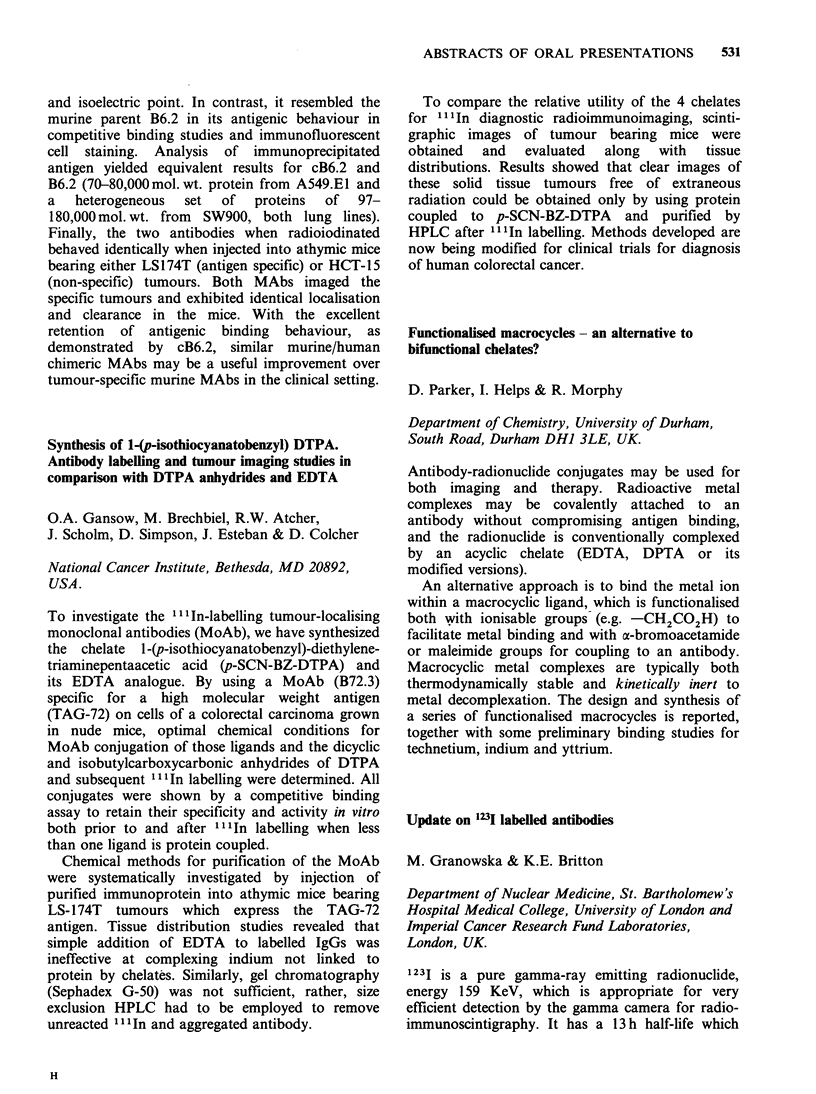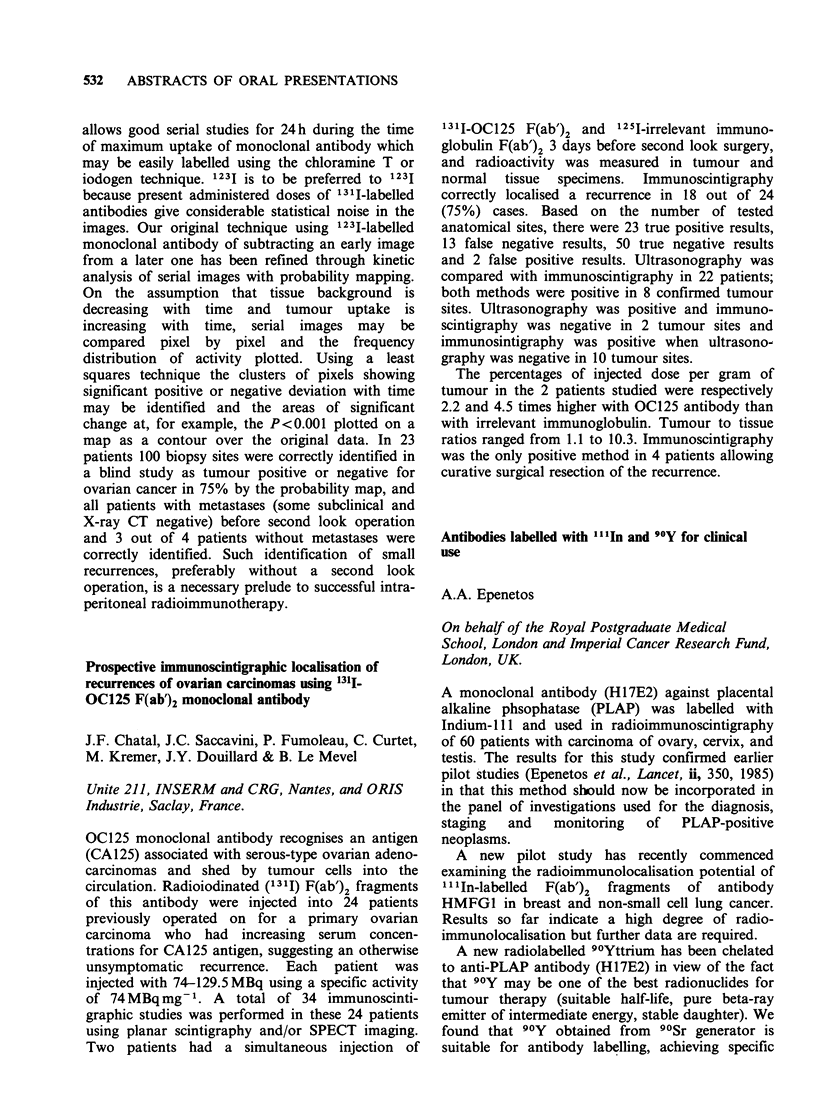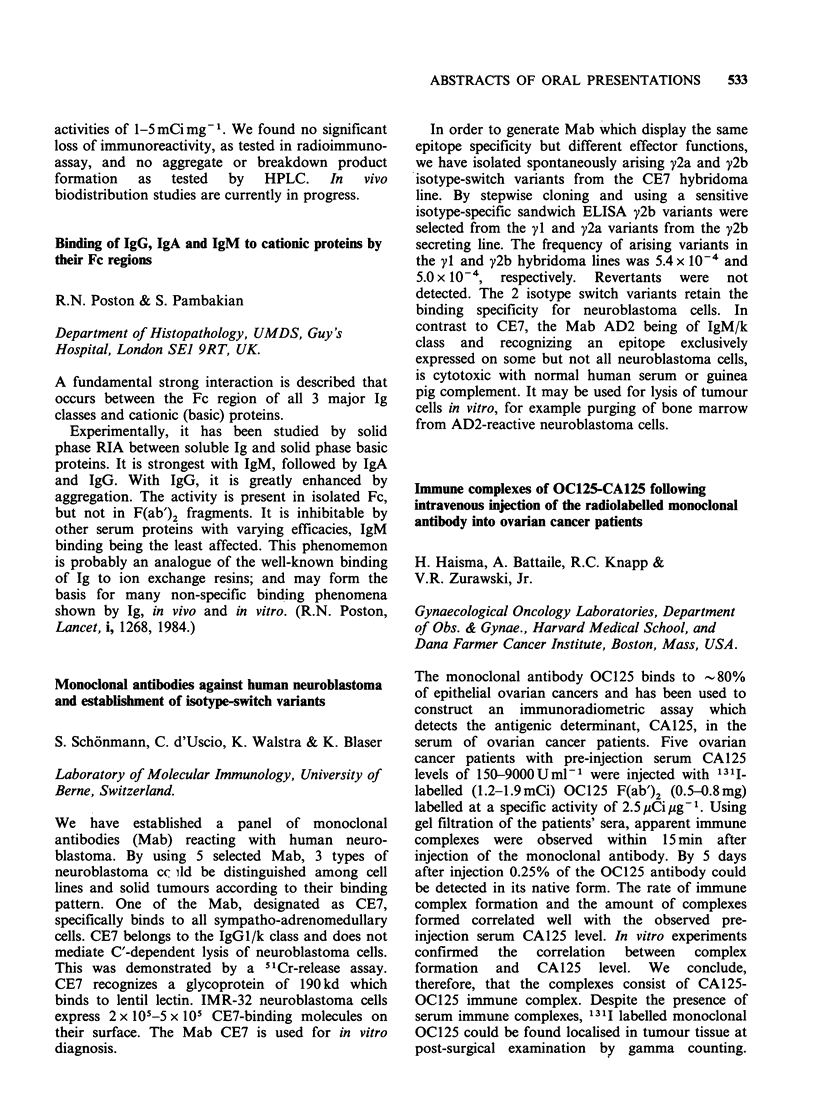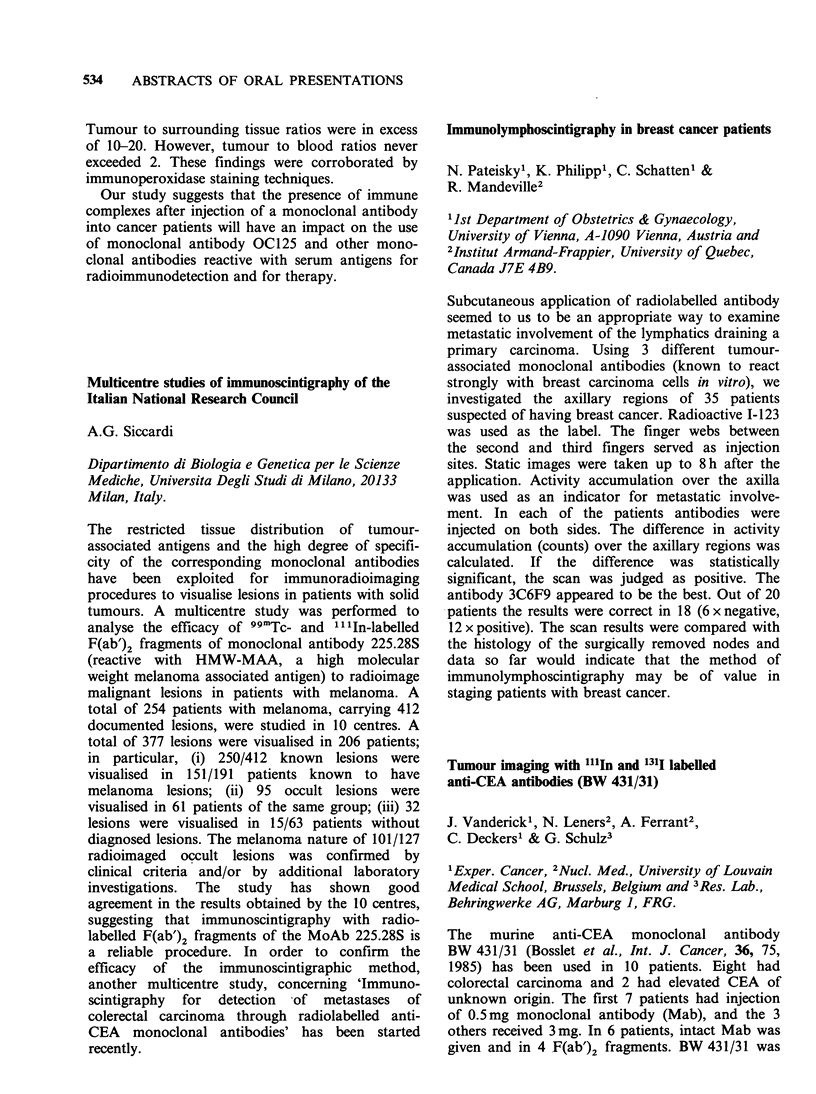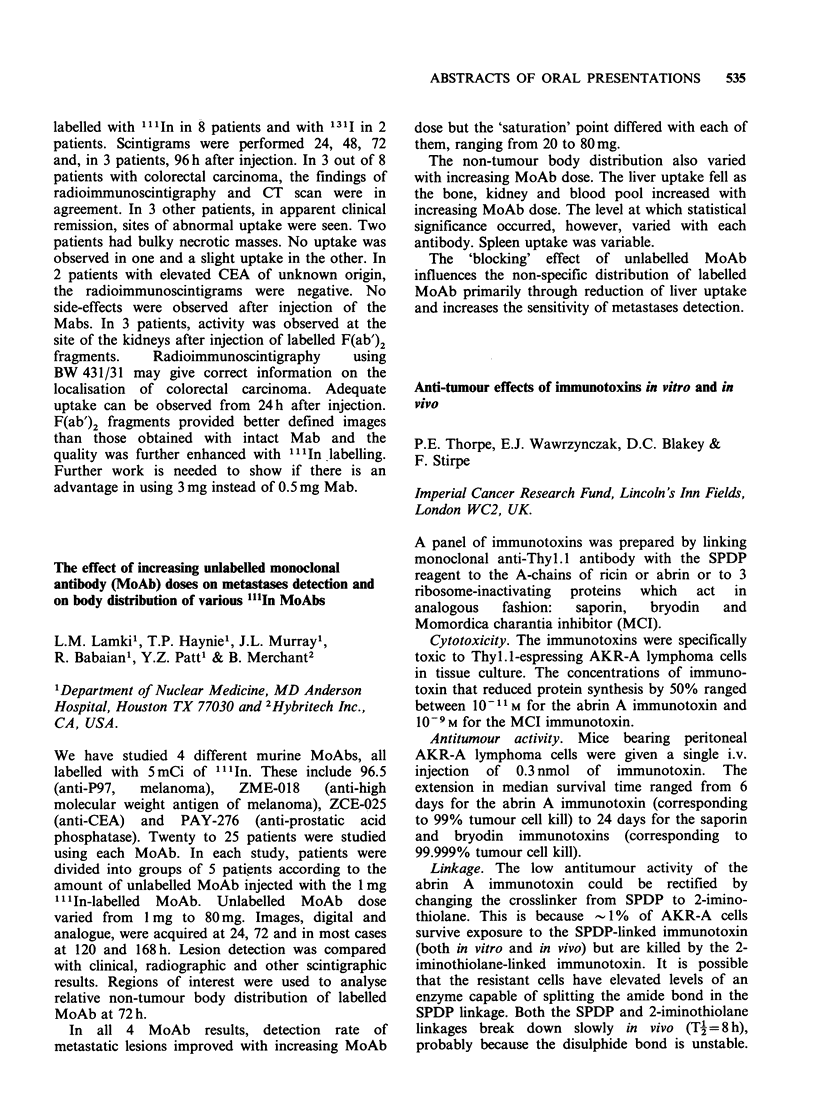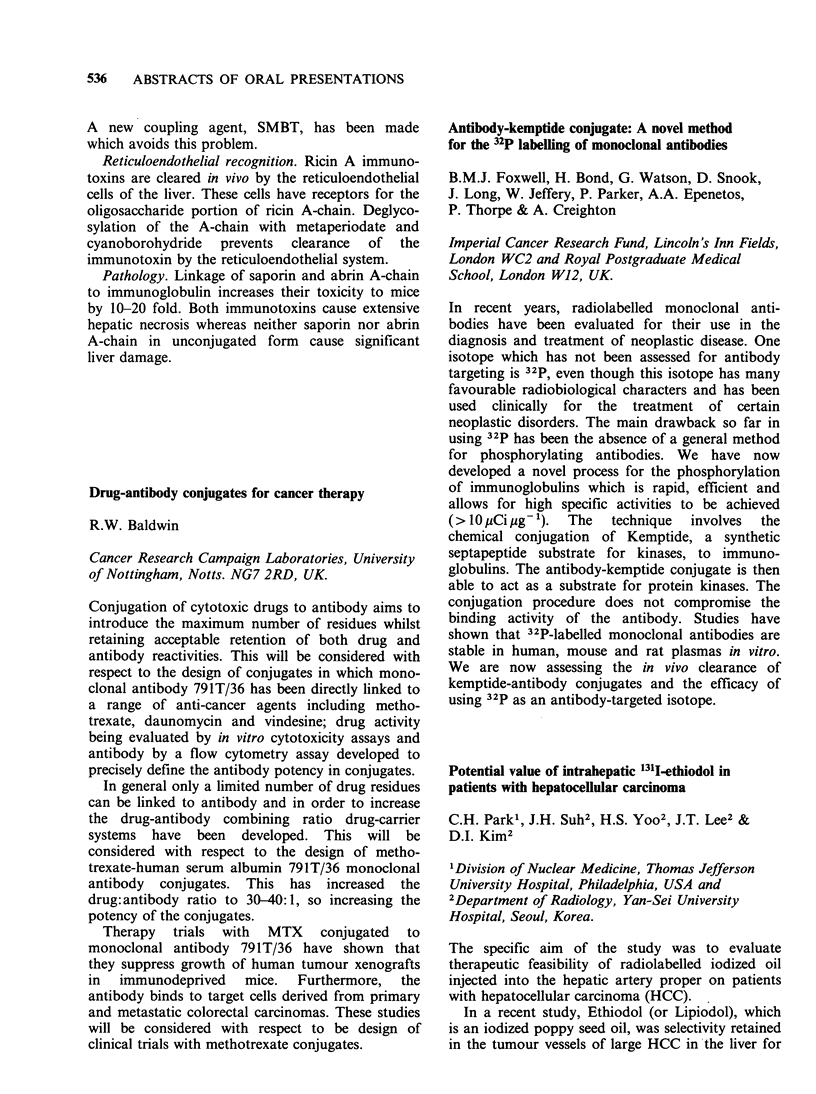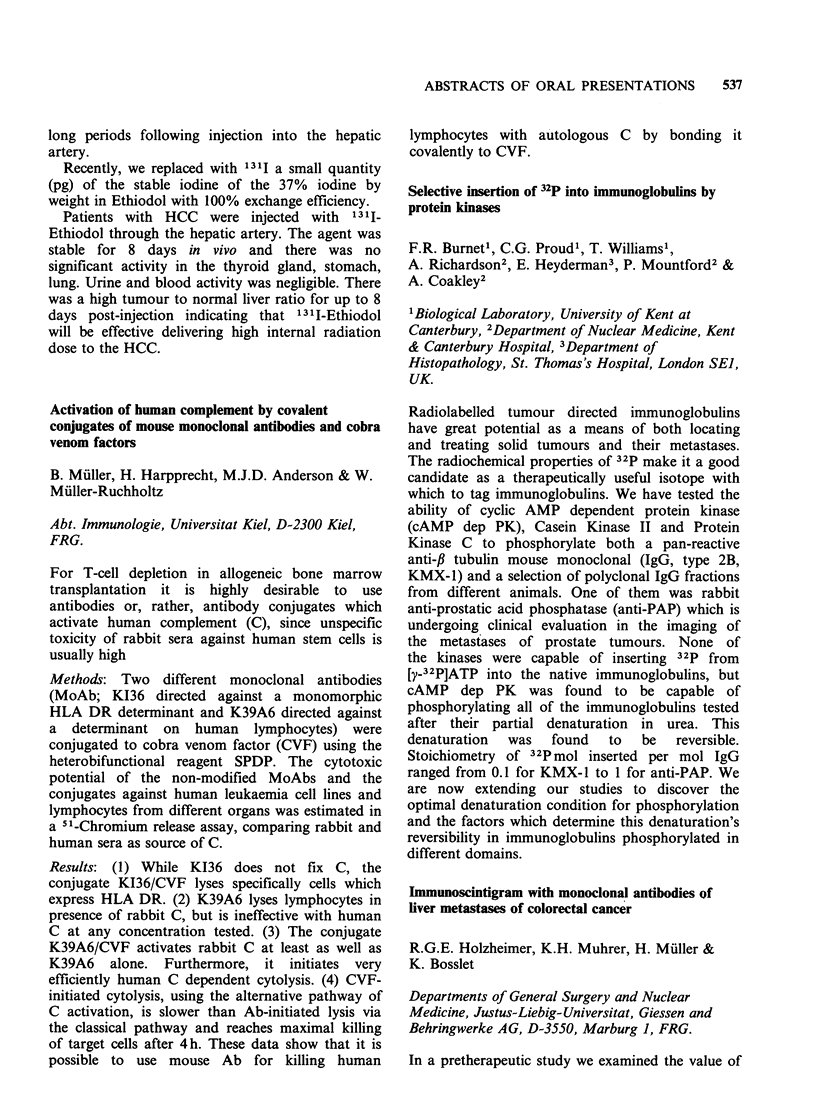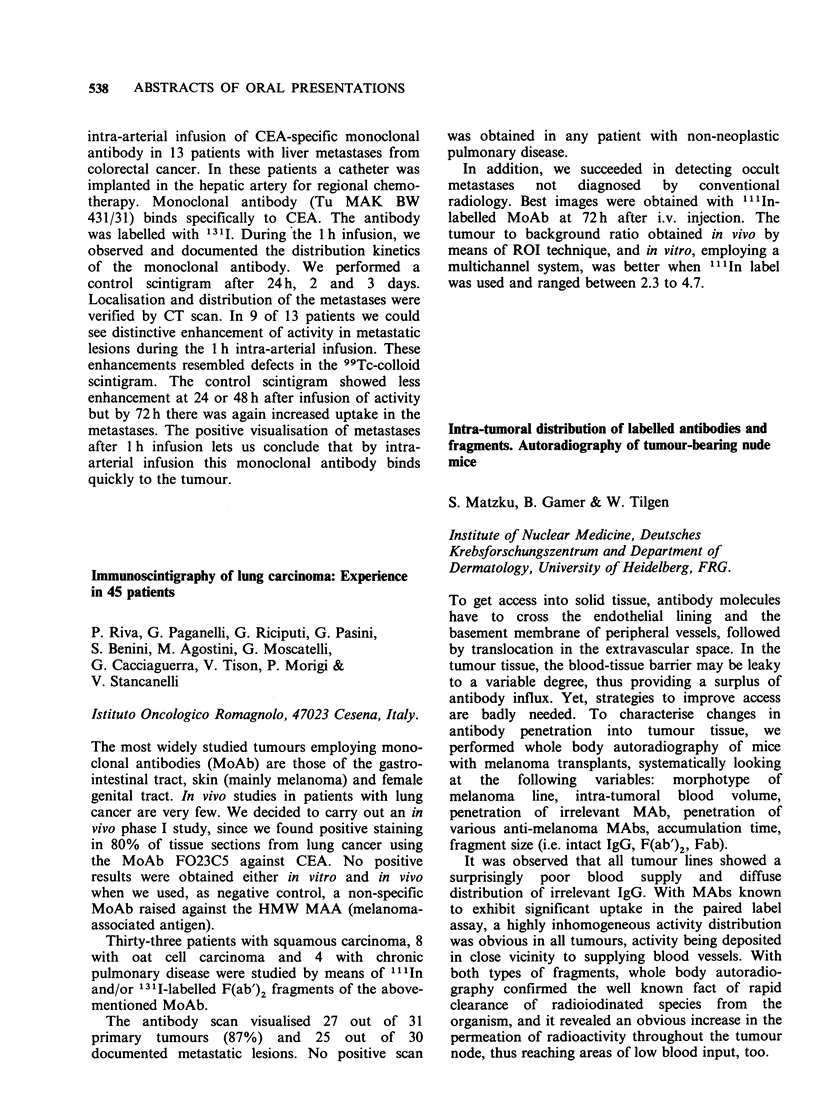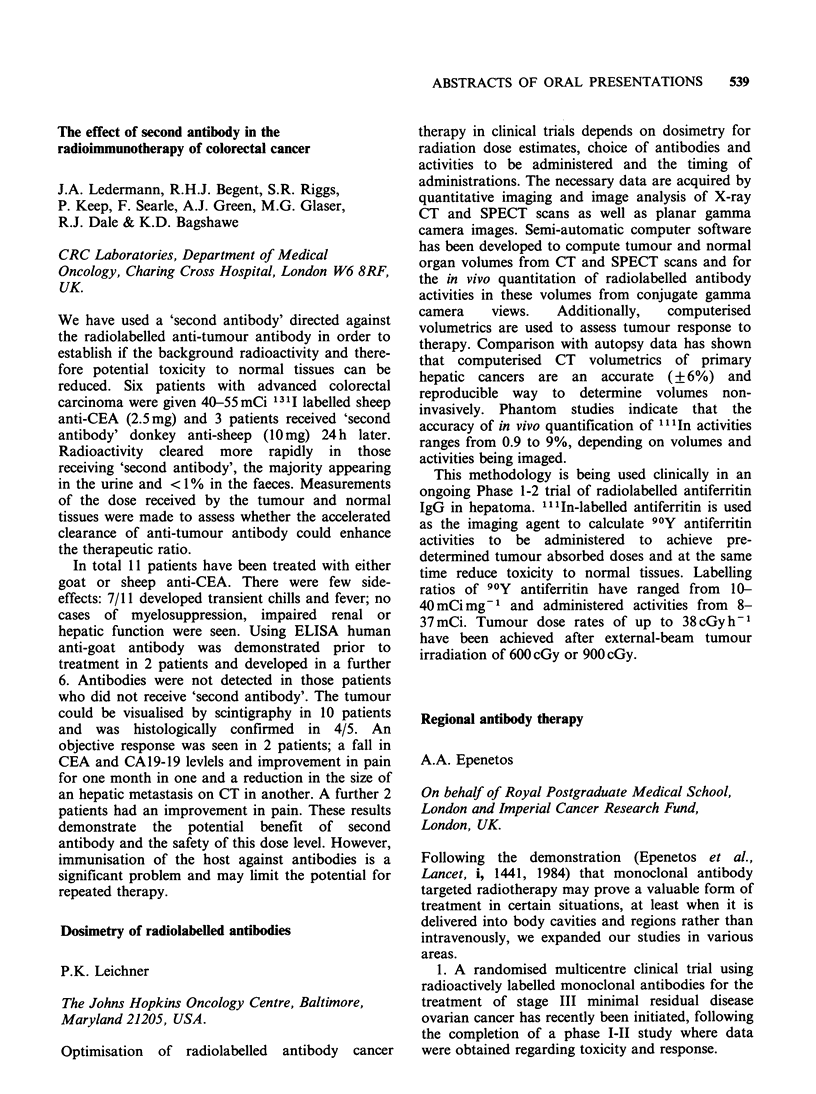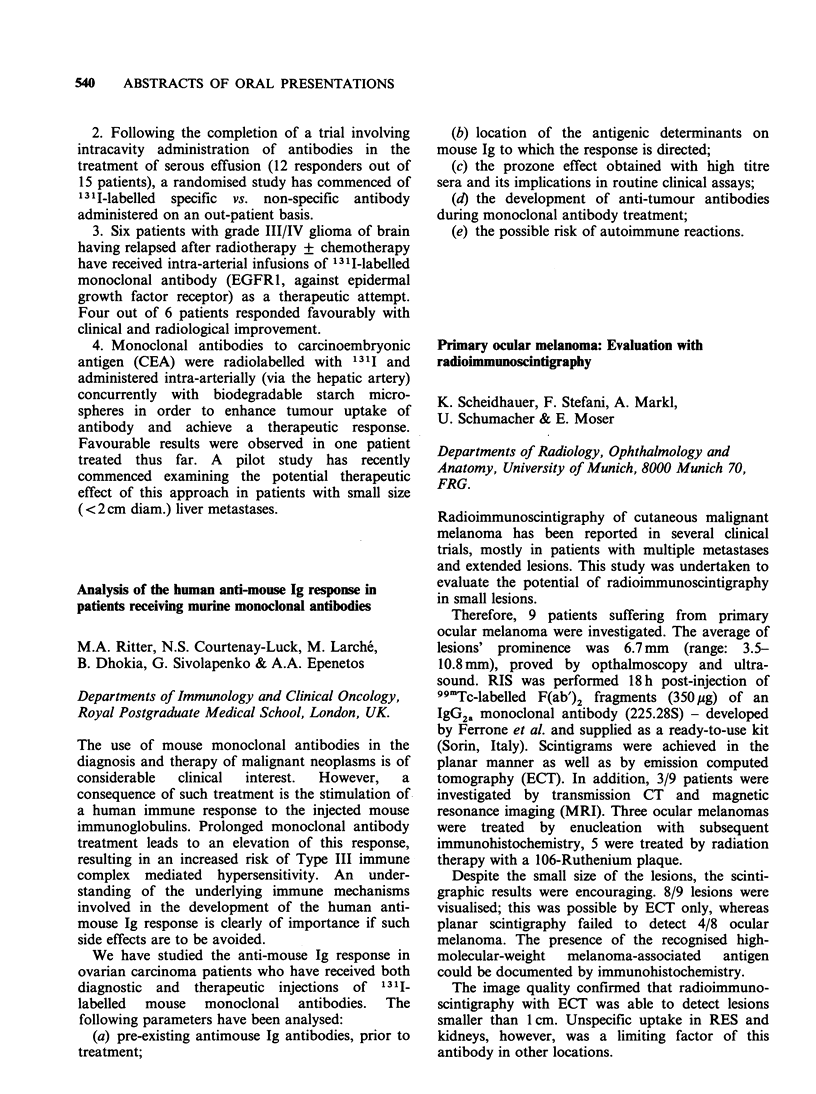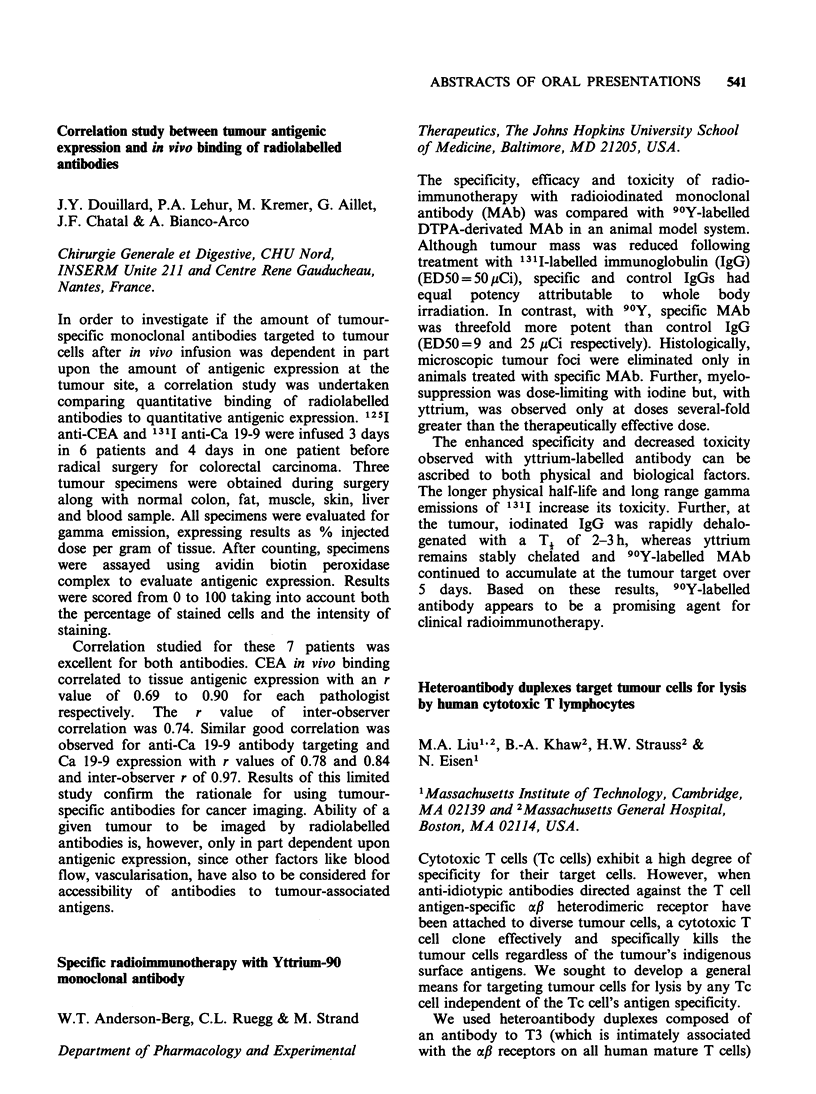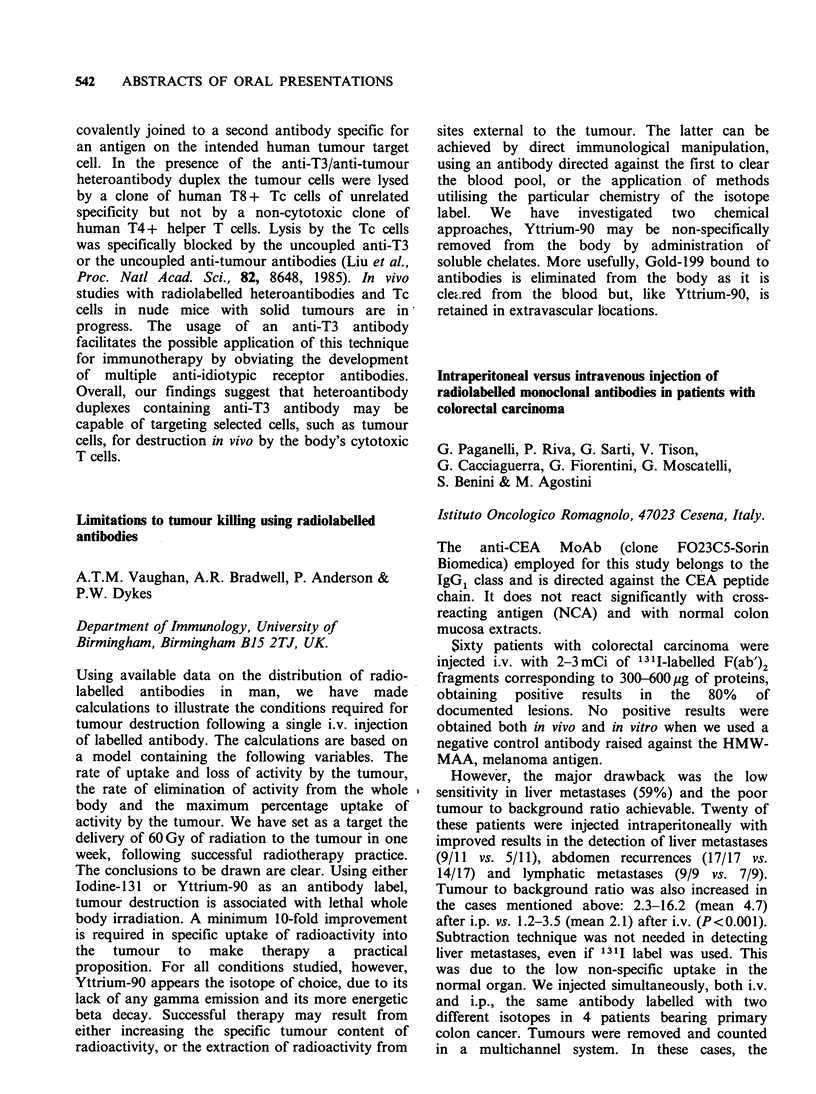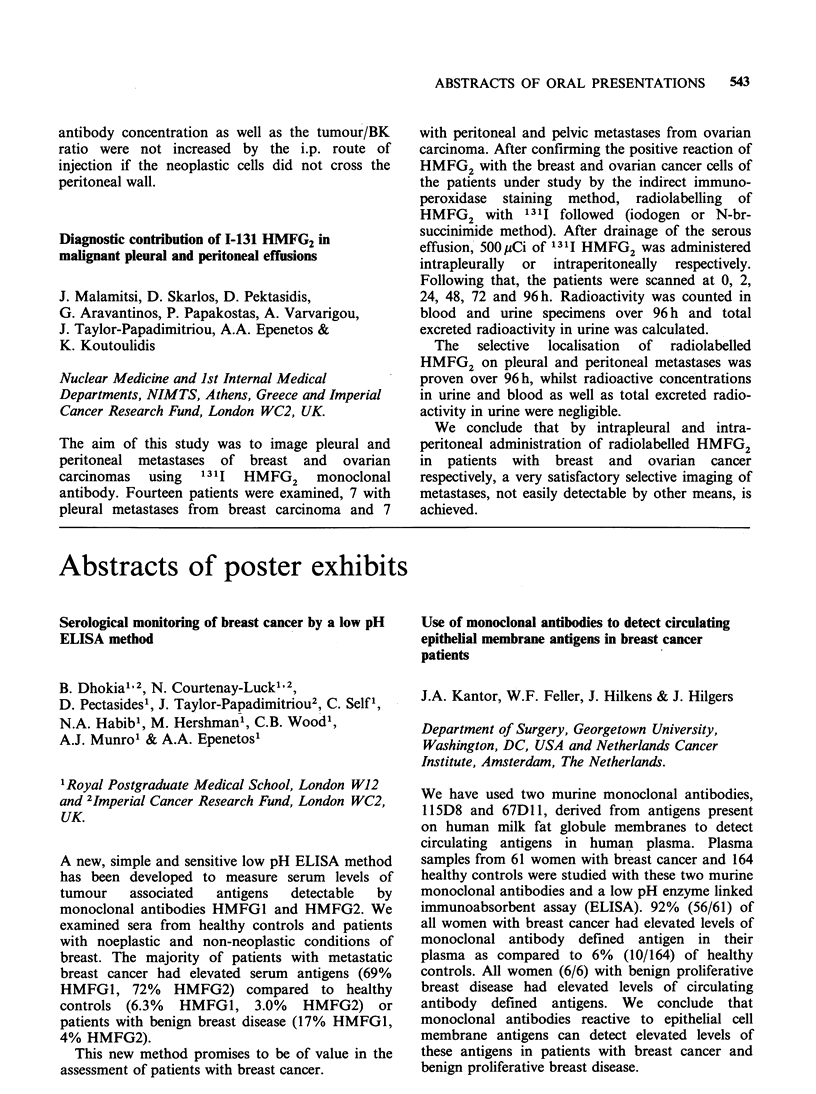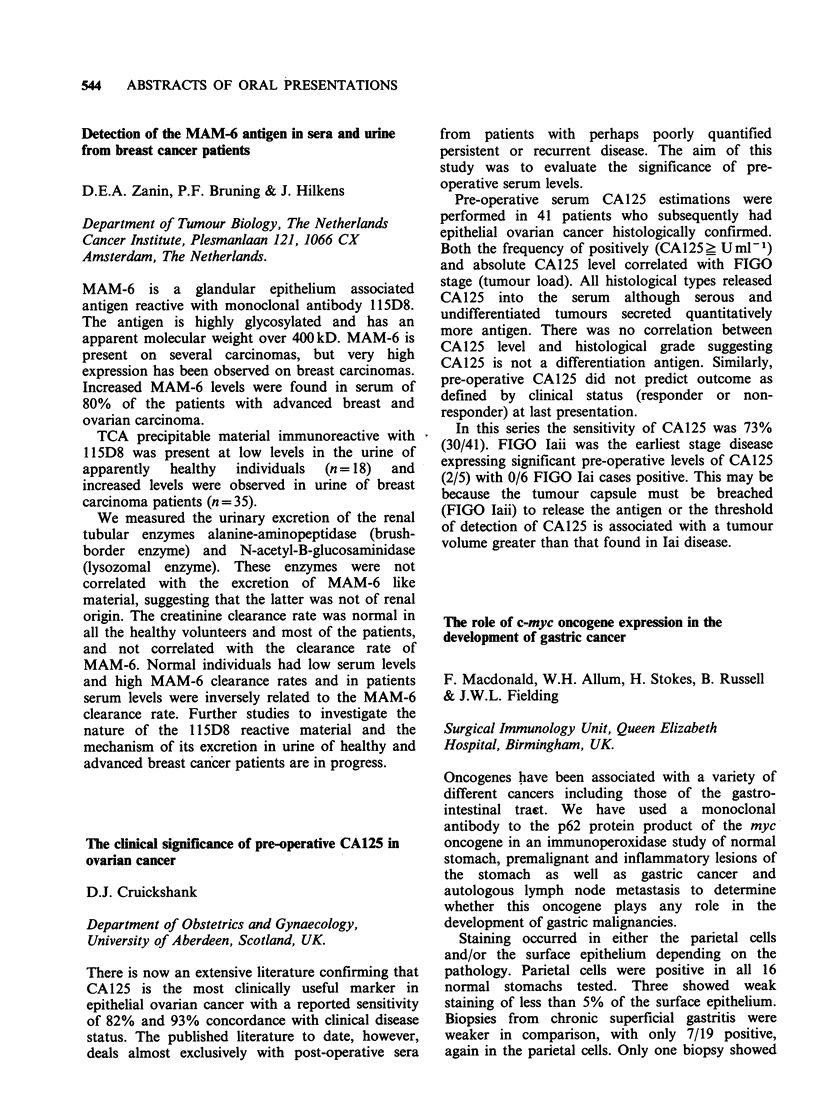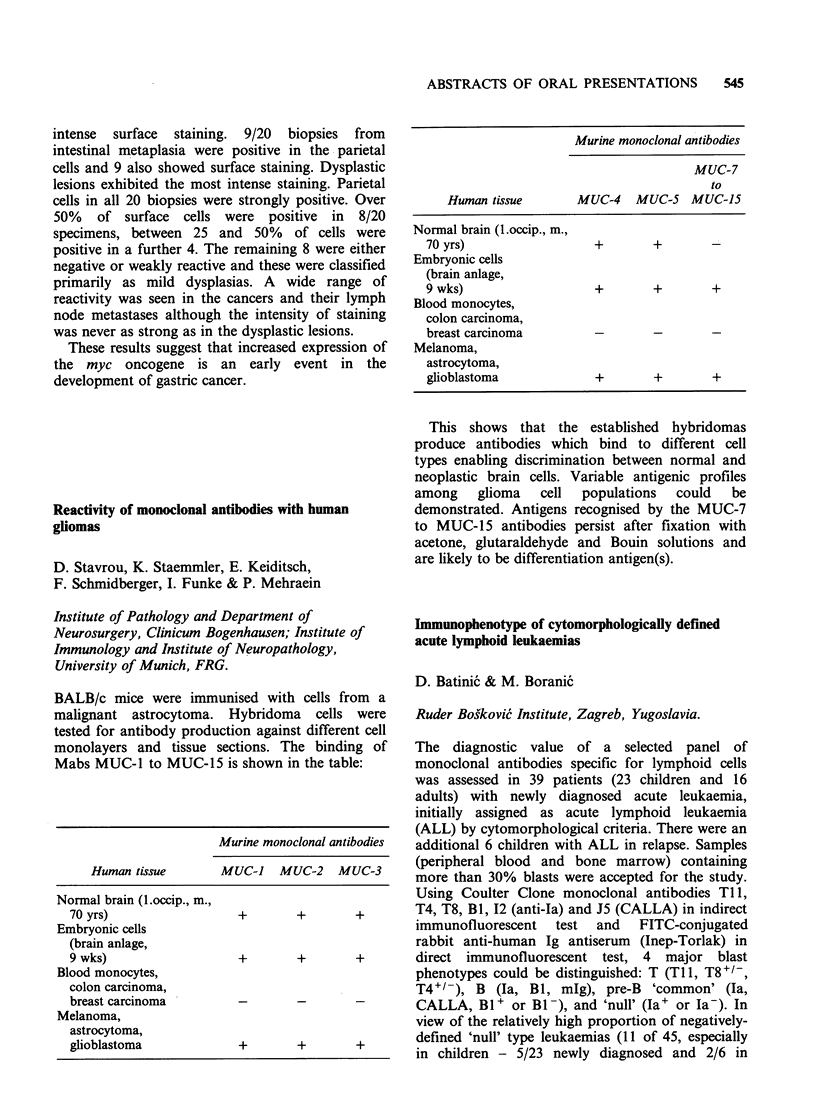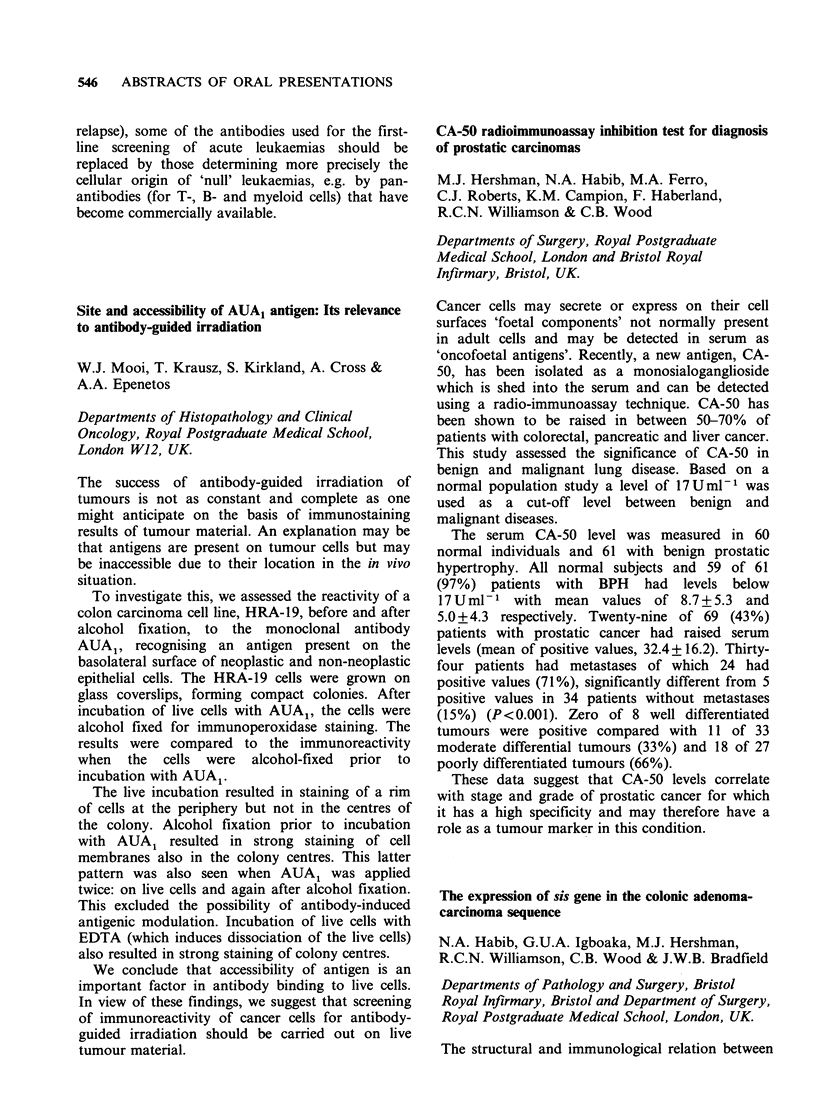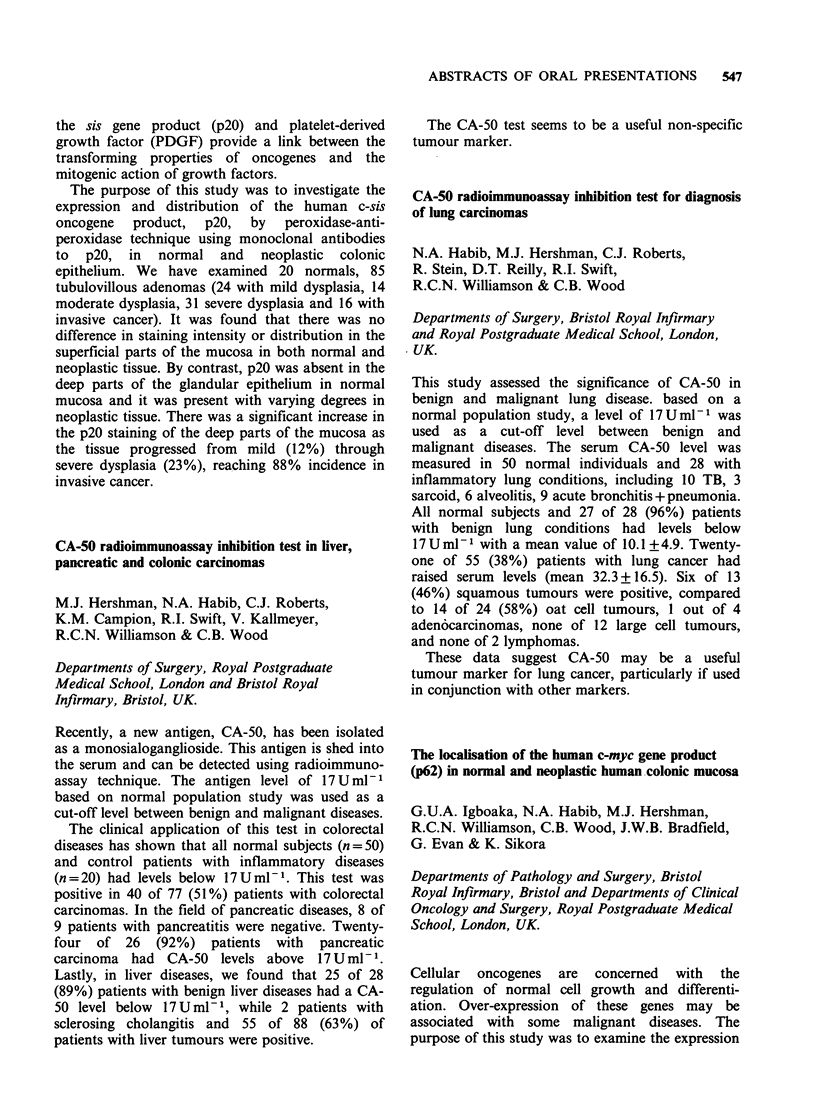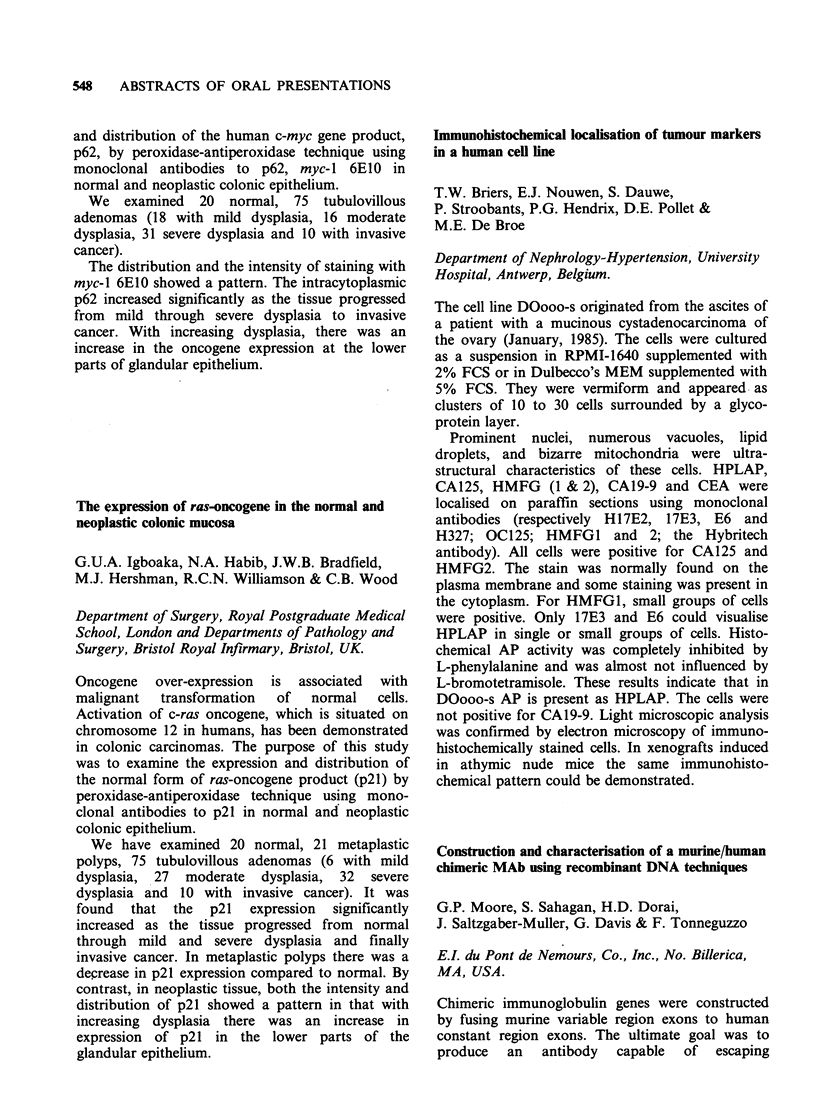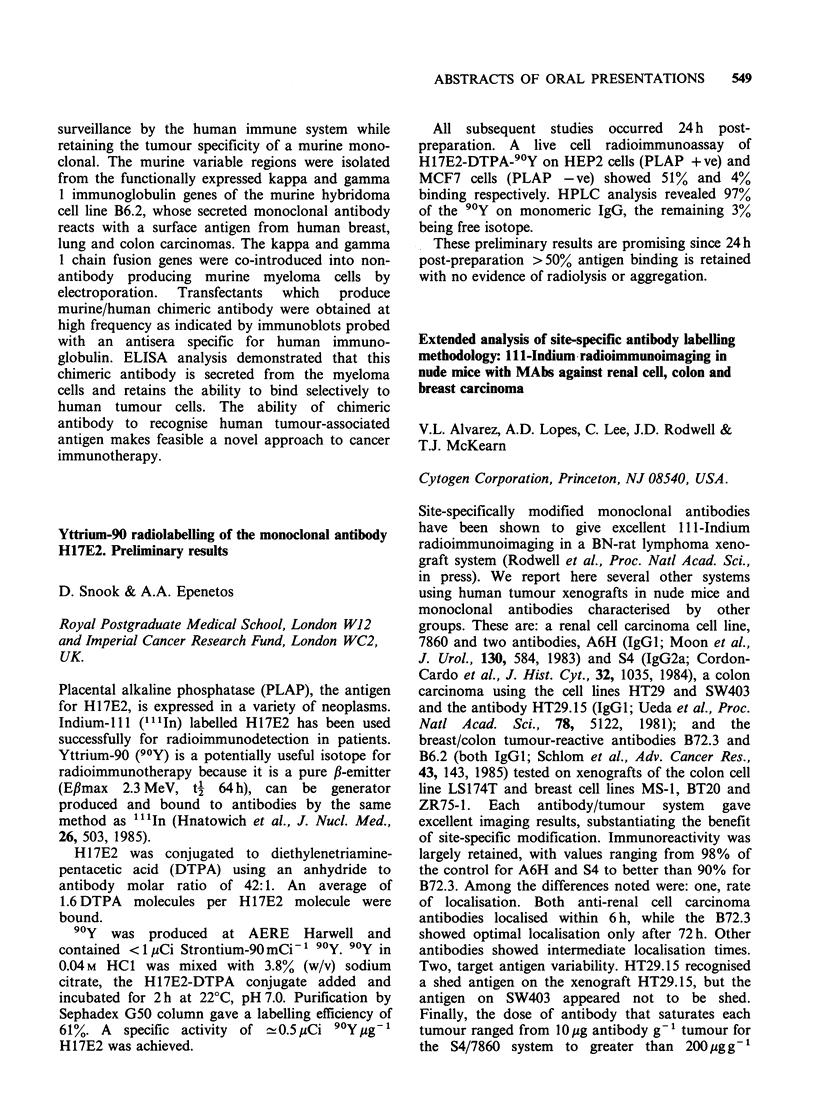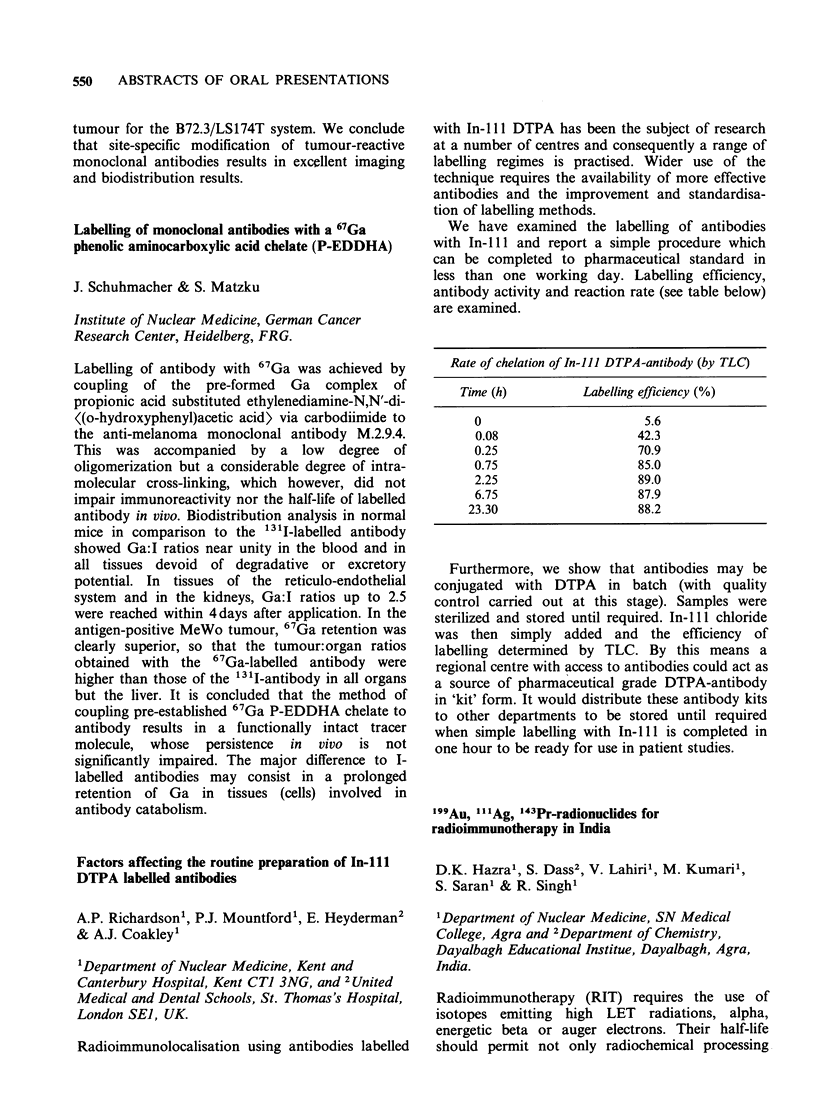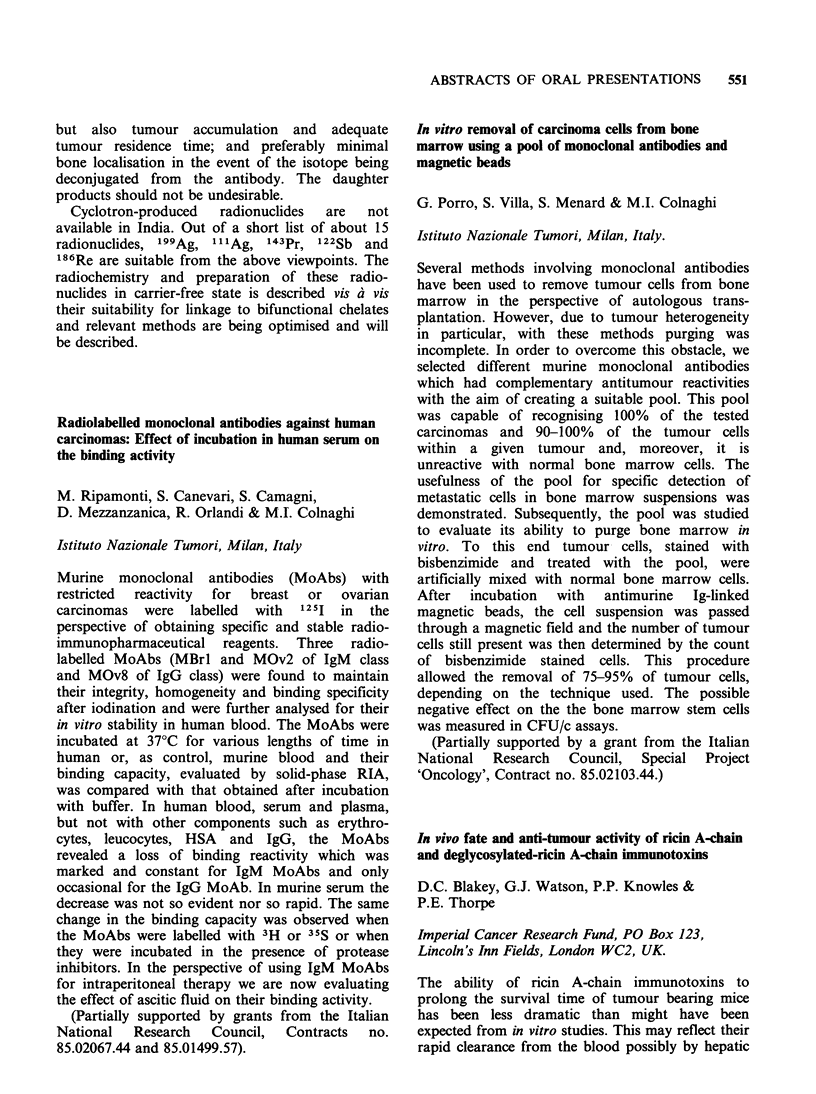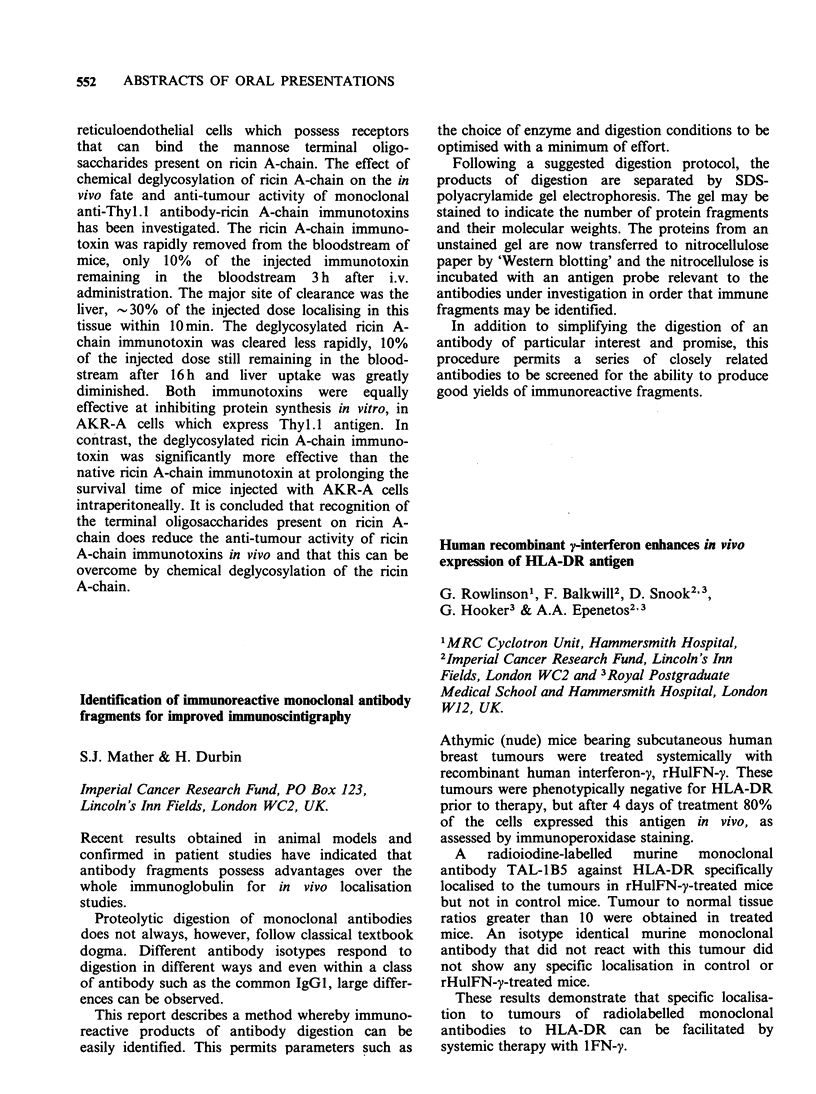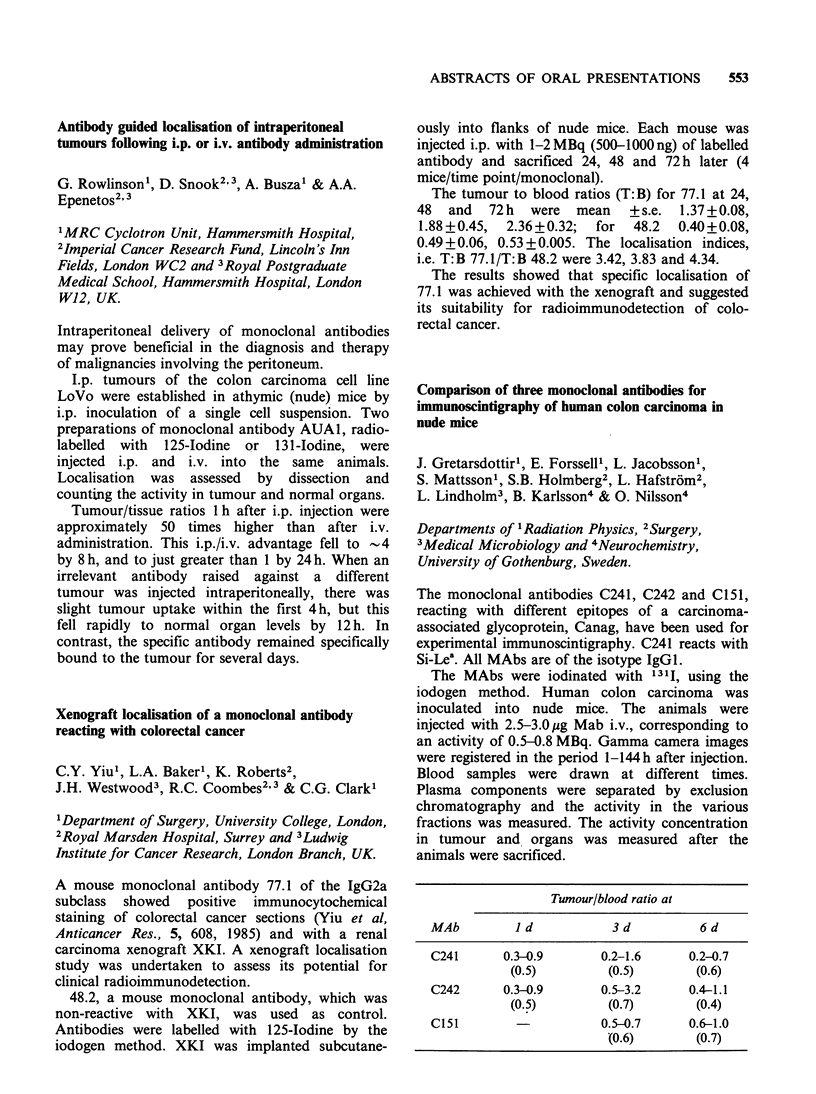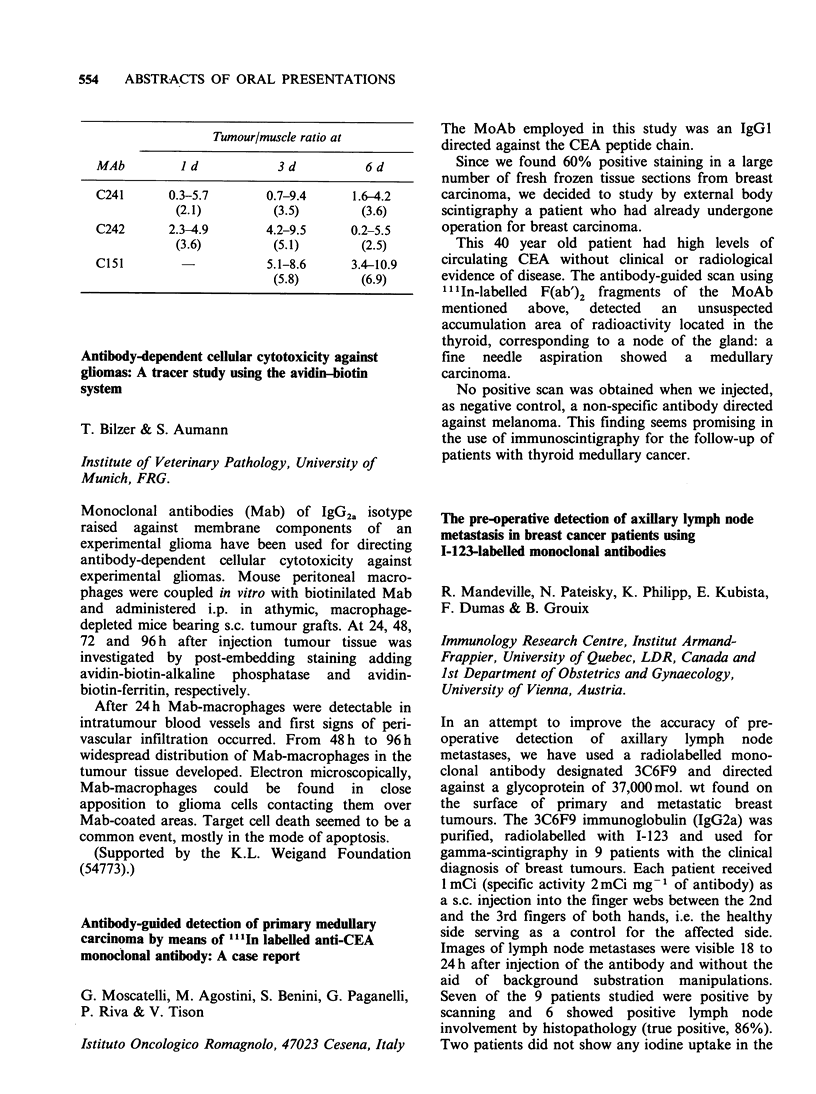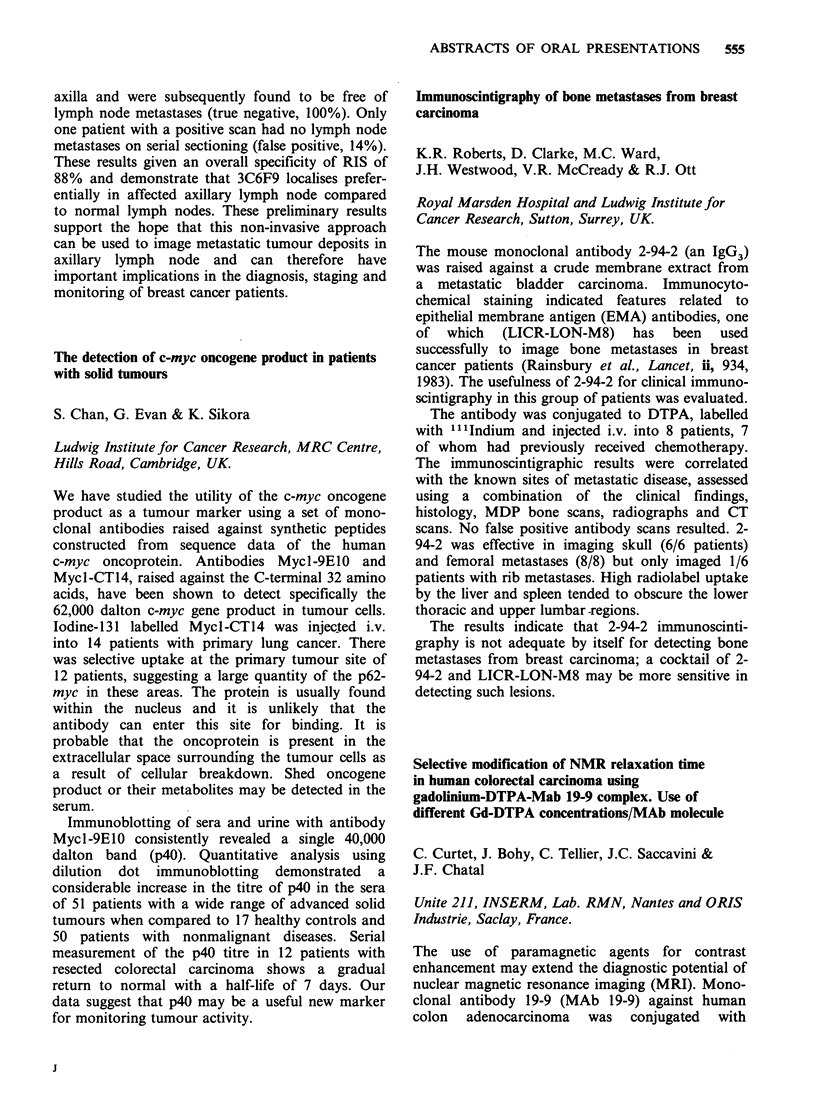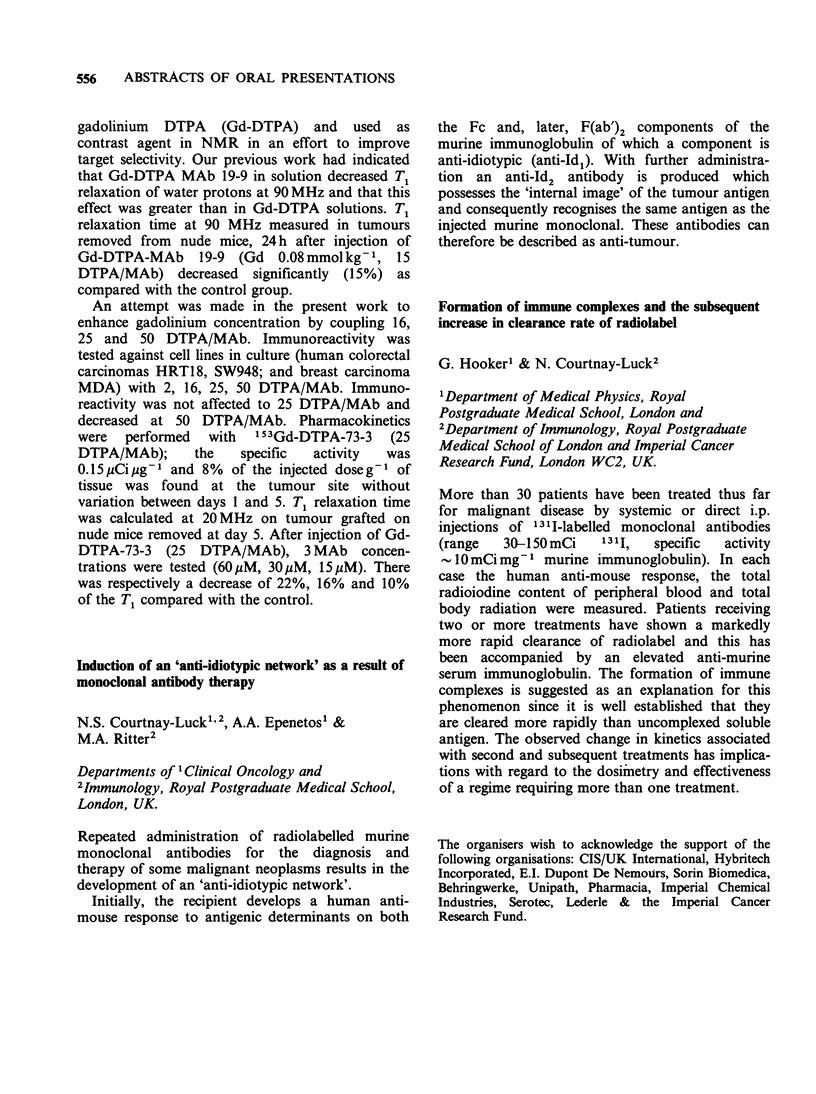# Advances in the applications of monoclonal antibodies in clinical oncology. London, 28-30 May 1986.

**DOI:** 10.1038/bjc.1986.208

**Published:** 1986-09

**Authors:** 


*Br. J. Cancer* (1986), **54**, 525–556

### Advances in the applications of monoclonal antibodies in clinical oncology

### University of London Royal Postgraduate Medical School

*Held in the Wolfson Institute, 28–30 May, 1986*

### Abstracts of oral presentations

### Interaction of monoclonal antibodies with the immune system

M. Clark, R. Benjamin, C. Bindon, M. Bruggeman, S. Cobbold, L. Gilliland, G. Hale, S. Qin, H. Tighe & H. Waldmann

*Department of Pathology, University of Cambridge, UK.*

We have been deriving rat monoclonal antibodies directed against human cell surface antigens for therapeutic use. In parallel to this work we have also derived antibodies against mouse cell surface antigens for use in model systems of human serotherapy. A number of conclusions can be made.

1. Rat monoclonal antibodies can be derived which exploit the natural effector mechanisms such as human complement, ADCC and cytotoxic T-cells.
2. CAMPATH-1, a human complement fixing rat IgM specific for human lymphocytes is able to eliminate lymphocytes from bone marrow and hence reduce the incidence and severity of GVHD.
3. Rat IgG2b antibodies are able to mediate ADCC with human K-cells.
4. Suitable pairs of rat IgG2b antibodies synergise in complement lysis.
5. Rat IgG2b antibodies to mouse T-cell subsets eliminate cells *in vivo* and cause marked immunosuppression of T-cell mediated responses.
6. Serotherapy with anti-(mouse T-cell) antibodies can facilitate the reconstitution of sub-lethally irradiated mice with T-cell depleted mismatched marrow grafts.
7. Rat antibodies to the mouse L3/T4 antigen are capable of inducing tolerance to rat and human immunoglobulin.
8. Mice which have been rendered tolerant to rat IgG2b are still capable of making an anti-idiotype response to cell-binding antibodies. The exceptions to this are antibodies to the L3/T4 antigen.
9. The anti-idiotype response in tolerised mice has implications for human serotherapy with human or chimeric monoclonal antibodies.

Course Organiser: A.A. Epenetos, Royal Postgraduate Medical School & Hammersmith Hospital, London W12 0HS and Imperial Cancer Research Fund.

### The Ca antigen

H. Harris

*Sir William Dunn School of Pathology, University of Oxford, UK.*

The Ca antigen was detected by screening matched pairs of hybrid cells, one member of which was malignant and the other not. The antigen was found on the malignant, but not on the non-malignant hybrid cells, and was later detected in a wide range of human malignant tumours. The expression of the antigen in the tumours varied in intensity. In some cases there was generalised labelling of virtually all the malignant cells in the tumour; in other cases the labelling was patchy. The antigen was also found on a number of normal epithelia, notably the urothelium, the luminal surfaces of the epithelia of the oviduct, apocrine sweat glands, the ducts of eccrine sweat glands and of the epididymis, and the trophoblast of the developing embryo. A totally unexpected finding was the presence of the antigen on Type II pneumocytes, for which it appears to be specific.

Monoclonal antibodies directed against the Ca antigen have found some clinical usefulness in diagnostic cytology, but the main interest lies in the antigen itself. Substantial chemical characterisation of the antigen has been achieved, and it has turned out to be a mucin to which the name EPITECTIN has been given. A most remarkable characteristic of this mucin is that it is inducible *in vitro* by high concentrations of sodium lactate. Detailed analysis of the induction by lactate has provided strong evidence that the basic effect is on osmolarity. The

© The Macmillan Press Ltd., 1986

antigen is induced when the osmolarity of the surrounding medium is substantially increased. It is likely that lactate is the physiological agent involved in the body, although other substances may achieve the same effect *in vitro*. More recent studies *in vivo* will also be reported.

**Recombinant antibodies for clinical use**

M.W. Bodmer

*Celltech Ltd., Bath Road, Slough SL1 4DY, Berks, UK.*

Interest in the clinical use of monoclonal antibodies has created a demand for techniques to tailor immunoglobulin molecules to make best use of their specificity and binding properties. Recombinant DNA techniques provide great flexibility in joining combining domains with cytotoxic or biological effector functions which may be better suited to *in vivo* use than natural immunoglobulin molecules or fragments. We can 'humanise' antibodies, create well-defined fragments, incorporate specific labelling sites and generate protein–protein fusions. In addition, by interchanging combining sites we can make fairly homogeneous sets of reagents directed at a variety of tumour markers.

Work is in progress in our laboratory on isolation and manipulation of genes encoding a number of antibodies specific for colon and ovarian tumour markers. In parallel we are developing gene expression systems to allow high level expression of recombinant material.

**Replacing the complementarity-determining regions in a human antibody with those from a mouse**

J. Foote, P.T. Jones, P. Dear, M.S. Neuberger & G. Winter

*Medical Research Council Laboratory of Molecular Biology, Hills Road, Cambridge CB2 2QH, UK.*

The hypervariable domains of an antibody consist of a $\beta$-sheet framework with hypervariable regions (or complementarity-determining regions – CDRs) which fashion the antigen binding site. Can the antigen site be transplanted from one framework to another by grafting the CDRs? We have used the CDRs from the heavy chain variable region of mouse antibody B1-8 which binds the hapten NP-cap (4-hydroxy-3-nitrophenacetyl-caproic acid; $K_{NP\text{-}cap} = 1.2\,\mu M$) to replace the corresponding CDRs of a human myeloma protein. We find that in combination with the B1-8 mouse light chain, the new antibody has acquired the hapten affinity of the B1-8 antibody $K_{NP\text{-}cap} = 1.9\,\mu M$). Such 'CDR replacement' may offer a way of constructing human monoclonal antibodies from the corresponding mouse monoclonals.

**Monoclonal antibodies to oncogene products**

K. Sikora, G. Evan, S. Chan, J. Stewart & J. Watson

*Department of Clinical Oncology, Royal Postgraduate Medical School, Hammersmith Hospital and Ludwig Institute for Cancer Research, MRC Centre, Cambridge CB2 2QU, UK.*

The demonstration that unique segments for DNA, constant in location and conserved in evolution, are involved in growth control opens new avenues for clinical research. The function of these oncogenes and their relevance to specific disease processes needs to be elucidated. The function of the products of several oncogenes has now been determined; in all cases it is related to growth control.

To evaluate the clinical significance of abnormal gene expression, monoclonal antibodies have been constructed to several human oncoproteins. These include c-*myc*, c-*sis*, c-*ras*, c-*myb* and N-*myc*. We have studied archival material from patients with testicular, breast and colorectal cancer by immunohistology and flow cytometric analysis of isolated nuclei. We have compared expression of the c-*myc* gene with that of c-*ras* and related expression levels to the histological differentiation state of the tumour. In colorectal cancer, it is clear that benign polyps and well-differentiated tumours express c-*myc* in greatest abundance. Poorly differentiated tumours express lower levels in the testis, seminomas have the highest level of c-*myc* expression with undifferentiated rapidly growing tumours the least. We have related c-*myc* expression histologically to flow cytometric analysis using isolated nuclei from paraffin blocks. Good correlation exists between both sets of data. Our findings suggest that the c-*myc* oncogene product may play an important role in the evolution of neoplasia. Clinical correlation of differentiation state and oncoprotein levels may provide potentially useful information for patient care.

### Monoclonal antibody imaging and therapy of solid tumours

S.M. Larson

*Nuclear Medicine Department, National Institutes of Health, Bethesda, MD, USA.*

We used $^{131}I$-labelled Fab fragments of antibodies for diagnosis in malignant melanoma. A sensitivity of 88%, and a specificity of 100% was observed in selected patients. A Phase I trial of treatment of malignant melanoma was begun, using high dose radiolabelled anti-p97 and anti-chondroitin sulphate proteoglycan FABs. We found that we could target multiple doses of therapeutic MOABs to human tumours safely. Among 3 patients who received more than 450 mCi, 2 had an anti-tumour response – one a prolonged stabilisation, and a second a partial regression. A more extensive trial is currently underway. Toxicity is seen as marrow suppression at one month post-treatment. This predictable radiation effect has not resulted in severe clinical side-effects.

B72.3 is an immunoglobulin that recognises TAG-72, a high molecular weight mucin-like compound that is produced by breast, ovarian and colon cancers. We studied 25 patients with colon cancer to determine localisation after intravenous administration of $^{131}I$-labelled B72.3. All the patients underwent surgical exploration after injection of the radiolabelled MOAB. If tumour/normal tissue ratio of $>3.0$ is taken as a cut-off, 85% of tumours were positive. Also, patients were co-injected with a non-specific $^{125}I$-labelled antibody of the same sub-class. Ratios of specific to non-specific ranged from 2.5–10.0. Gamma camera imaging showed excellent targeting to lesions, which was most favourable at one week post-injection.

B72.3 labelled $^{131}I$ was injected i.p. in 5 patients with *pseudomyxoma peritonei.* All patients had excellent localisation which correlated well with subsequent surgical findings. Clearance from the tumour site was prolonged, with a T-1/2 of $>4.0$ days. In 3 of the 5 patients, CT scans of the abdomen were negative, at a time when the imaging study was positive. Thus, the radiolabelled antibody detected disease not evident with other methods. Localisation of antibody to lesions was avid, with maximum concentrations of 0.18% injected dose per gram of tumour. Dosimetry is favourable for therapy.

### Leucocyte-associated antigens

G. Janossy

*Department of Immunology, Royal Free Hospital School of Medicine, London NW3 2QG, UK.*

Leucocyte typing workshops have established panels of monoclonal antibodies to leucocyte antigens leading to the standardisation of the diagnosis of leukaemias and lymphomas. A number of antibodies have distinct therapeutic possibilities; (i) mismatched haploidentical bone marrow transplantation (BMT) and (ii) autologous BMT.

(i) The tissue reactivity of HLFA (human leucocyte functional antigen; CD18) has been established. HLFA appears on the B cell lineage at the pre-B cell stage and in the T lineage on the large thymic blasts but is absent on $TdT^+$ B cell precursors and on myeloid and erythroid precursor cells. As this antibody is efficient in blocking leucocyte function, it is suggested that it could be infused around the time of transplantation to patients receiving mismatched BM in order to promote the 'take' of haploidentical BM. BM regeneration in these haploidentical BMTs has been rapid. Further studies are warranted to study anti-HLFA in BMT for leukaemia.

(ii) A sensitive assay has been developed and is used in the MRC UKALL-X trials in order to establish the lytic efficacy of McAbs of IgM class against common ALL T-ALL and B cell lymphomas. This assay is based on restaining residual blasts following C lysis with an independent marker such as nuclear TdT (C-ALL and T-ALL) and anti-immunoglobulin (B lymphoma). We can get greater than 4 log kill in 75% of the malignancies with rabbit complement and, surprisingly, in 50% of cases with autologous human complement. Suitable antibodies for this purpose are: RFAL3 (CD10; IgM, human complement fixing), RFB7 (CD20; IgM, human complement fixing), SB4 (CD19; IgM, partially human complement fixing, and RFT2 (CD7; IgG2, rabbit complement fixing).

### Patterns of expression of keratins and mucins by mammary epithelial cells

J. Taylor-Papadimitriou[1], S. Gendler[1], J. Burchell[1] & J. Bartek[2]

[1]*Imperial Cancer Research Fund, Lincoln's Inn Fields, London WC2A 3PX, UK; and* [2]*Res. Inst. Clin. Exp. Oncol., Zluty Kopec 7, 602 00 Brno, Czechoslovakia.*

Antibodies to two classes of molecules which are produced by most epithelial cells have been used to define differentiation phenotypes in epithelial cell lineages and to relate these to the malignant phenotype. The first are the keratins which make up the intermediate filaments of epithelial cells and the second are mucin-like molecules, i.e. glycoproteins containing a high proportion of 0-linked sugars. The monospecific and pleurispecific antibodies to keratins have been useful in defining cell lineages and characterising the malignant phenotype in terms of these lineages in the mammary gland. Antibodies to the mucin-like molecules (which can be expressed on the cell membrane) are widely used for detecting carcinoma cells and their products in body tissues and fluids and for localising tumours in patients. Molecular and immunological .analysis of the large glycoproteins shows a size heterogeneity, some of which is attributable to genetic polymorphism. Preliminary results with a cDNA probe corresponding to part of the gene coding for the core protein of the mucin expressed by breast cancer cells show that the expression of this gene is indeed only seen in epithelial cells. Antibodies reactive with the core protein but not with the fully processed mucin react with breast cancers, but not normal breast epithelium suggesting that there may be incomplete processing of the mucin in some cancers.

**HLA antigens in human tumours**

M. Moore

*Department of Immunology, Paterson Laboratories, Christie Hospital and Holt Radium Institute, Manchester M20 9BX, UK.*

The status of human tumours with respect to the expression of the major histocompatibility complex (MHC) antigens is amenable to analysis by immunocytochemical techniques using monoclonal antibodies directed against heavy ($\alpha$) and light ($\beta_2$m) chain class I (HLA-A,B,C) molecules and sub-locus products (DP, DQ, DR) of the HLA-D region. The expression of these molecules has implications both for the inductive and effector phases of the anti-tumour immune response. Class I expression, common to virtually all nucleated normal cells, is reduced or lost in a significant proportion of primary epithelial cancers, especially of the breast, and could conceivably represent a mechanism by which tumour cells evade T cell recognition. Class II expression, previously thought to be largely restricted to cells of the immune system, may also be a constitutive property of some normal epithelia, but is most readily detectable on tissues – normal, inflamed, premalignant and malignant – undergoing hormonal or immune stimulation. In certain epithelia (e.g. small intestine) the function of class II molecules is to present antigen to T helper cells, but in some tumour systems (e.g. malignant melanoma) a depressive effect in the ability of primary tumours to stimulate autologous T lymphocytes has been reported. These findings indicate that the class II status of tumour cells has important functional implications for the tumour-host relationship.

**Uses of immunohistopathology**

D.Y. Mason & K.C. Gatter

*Nuffield Department of Pathology, University of Oxford, UK.*

Monoclonal antibodies are now of clearly established value in the immunocytochemical investigation of tumours of uncertain origin. The principal antibodies in this context are those against leucocyte common antigen and against cytokeratin, although anti-epithelial membrane antigen also has a role to play. Antibodies of these specificities which give good staining reactions on paraffin sections are now available. Use of these reagents should be mandatory whenever anaplastic morphology or poor tissue preservation make it difficult to reach a histological diagnosis.

A further use of immunocytochemistry in clinical oncology is in the detection of metastatic spread. Carcinomatous infiltration of lymph nodes may be detected by staining for epithelial markers, even when these cells are not visible by conventional microscopy. The method may also be applied to bone marrow smears, serous effusions and fine needle aspirates.

Finally, a novel application of monoclonal antibody immunohistology involves the use of antibody Ki-67 (directed against a proliferation-associated antigen), to assess the growth rate, and hence possible clinical behaviour, of tumours.

**A review of antigens of milk fat globule membranes and their value for diagnosis and prognosis of breast cancer**

J. Hilgers

*Division of Tumour Biology, The Netherlands Cancer Institute, 1066 CX Amsterdam, The Netherlands.*

Milk fat globule membranes (HMFGM) can easily be purified in large quantities from fresh human milk and represent the apical surface of the fully mature lumenal cell of the mammary gland. Many cell surface proteins in these membranes are not or hardly at all expressed in tumours of the mammary gland, but some are and these may be useful for diagnosis and prognosis of human breast cancer.

The so-called MAM-3 and MAM-6 group of antigens representing determinants of the HMFGM are found in the cell surface of breast cancer cells (Hilkens *et al., Int. J. Cancer,* **34,** 197, 1984). MAM-3 antigens appear to belong to antigens closely related to Lewis blood group 'a' and 'b' substances (Gool *et al., Biochem. Biophys. Res. Comm.,* **131,** 543, 1985; Gool *et al.,* in press). MAM-6 group antigens are expressed on a family of mucins of very high molecular weight.

Monoclonal antibodies against the MAM-3 group were used to see whether they are of prognostic value in predicting the course of breast cancer, e.g. in relation to presence and absence of steroid hormone receptors (Rasmussen *et al., Cancer Res.,* **45,** 1424, 1985).

Monoclonal antibodies against the MAM-6 group of antigens are useful as markers for the epithelial nature of tumours and indicators for a 'carcinoma' (Zotter *et al., Virchovs Archiv A., Path. Anat.,* **466,** 237, 1985) in the blood, because they are shed into the circulations (Hilkens *et al., Cancer Res.,* in press).

### A new marker for breast cancer. Use in immunohistochemical studies on formalin-fixed, paraffin-embedded tissues and at the electron microscope level

R. Mandeville, L. Giroux, F. Dumas, S. Fhali, B. Grouix, M.C. Zelechowska & I. Ajdukovic

*Institut Armand-Frappier, University of Quebec, LDR, Quebec, Canada J7E 4B9.*

A series of monoclonal antibodies (MAs) showing a different spectrum of reactivity to human mammary tissues of normal and neoplastic origin have recently been described (In *Monoclonal Antibodies: Diagnostic and Therapeutic Use in Tumour and Transplantation,* S.N. Chatterjee (ed) p. 63. PSB Publishing Co. Inc., Littleton, Mass., 1985). Studies on frozen sections of mammary tumours using indirect immunofluorescence (IF) enabled us to select one monoclonal antibody, namely 1F10B4 that recognises a cytoplasmic determinant highly expressed in most of the primary and metastatic breast carcinomas studied, and weakly (or not at all) in normal breast and non-breast tissues. More recently, we have compared its reactivity in frozen, acetone-fixed tissues using IF and formalin-fixed, paraffin-embedded sections using a standard avidin-biotin peroxidase technique. 1F10B4 reacted with 11 of the 21 primary tumours studied, demonstrating that in 52% of the cases, the antigenic determinant(s) detected on frozen acetone-fixed tissues were preserved after fixation in formalin. Moreover, localisation of breast cancer associated antigens recognised by 1F10B4 was performed by electron microscopy in Lowicryl K4M thin sections using a modified protein A-gold technique. This technique allowed the demonstration that 1F10B4 reacts with determinant(s) mainly expressed in the cytoplasm, only few reactivities were observed at the cell surface and on microvilli. This monoclonal antibody should be of biological, pathological and clinical value.

### A new monoclonal antibody (OVTL3) against human ovarian carcinoma that does not react with circulating tumour antigens

L.G. Poels & P. Kenemans

*Departments of Cytology & Histology and Gynaecology, Medical Faculty, University of Nijmegen, Geert Grooteplein N21, 6500 HB Nijmegen, The Netherlands.*

A cell suspension prepared from an ovarian endometrioid carcinoma was used to immunise BALB/c mice. Following a fusion protocol with PEG 4000 and the myeloma cell line Sp2/0-Ag 14, the hybridoma supernatants were screened by an immunofluorescence assay on cryostat sections of ovarian carcinomas and colon carcinomas. The selected clone (OVTL3) produced $IgG_1$ monoclonal antibody that did bind to freshly obtained cryostat sections and methanol fixed ascitic tumour cell clusters. The MoAb, however, did not bind to any paraffin processed tissue. The antibody reacted with >95% of the epithelial ovarian tumours (serous, mucinous, endometrioid and clear cell type); weak focally with 3 (14) endometrial carcinomas; negative with non-ovarian gynaecological tumours; negative with all other carcinomas. OC125 antibodies reacted with 85% of the ovarian carcinomas in this study, in both the serum assay and immunohistochemical assay. The use of radio-iodinated OVTL3 in a sandwich serum assay did not result in any binding of antigen in serum with high titres of CA125, neither was any binding observed in a cross serum sandwich assay with

OC125. From these studies, it appeared that OVTL3 did not detect circulating ovarian carcinoma antigens nor did it bind to different epitopes of the CA125 molecule. Binding to OVTL3 could only be achieved using freshly prepared Ov.Ca. extracts. SDS treatment abolished the binding activity. These results indicate that OVTL3 might be a good marker for histodiagnosis of ovarian cancer. The absence of binding to serum antigens might indicate that the OVTL3 could be useful for *in vivo* imaging and targeting therapy, as also suggested by the observation that the antibody remained bound for at least 24 h on cultured Ov.Ca. cells.

**MOv18 and MOv19: Two new monoclonal antibodies with restricted specificity against human ovarian carcinoma**

S. Menard, S. Canevari, S. Miotti, E. Tagliabue & M.I. Colnaghi

*Istituto Nazionale Tumori, Via G. Venezian 1, 20133 Milan, Italy.*

It has been demonstrated that ovarian carcinoma cells express immunogenic high molecular weight molecules in large quantities and it is against these that most anti-ovarian carcinoma antibodies are directed; however, similar molecules may also be expressed to a lesser extent by normal epithelial cells, thus creating problems concerning the specificity of the antibody.

To obtain monoclonal antibodies with more restricted specificities, we selected as immunogenic source, an undifferentiated ovarian carcinoma which was unreactive with previously obtained antibodies. Two new antibodies (MOv18 and MOv19) with similar patterns of reactivity were selected. They both recognise about 80% of ovarian carcinomas, but are negative with non-epithelial tumours and normal tissues as tested by immunofluorescence on frozen sections. In fact, reactivity was observed in only 3 out of 120 cases of non-ovarian carcinoma. Immunoprecipitation of radioiodinated membrane molecules from OVCa432 cells indicated that the relevant antigen of the two MoAbs is a 36–38 mol. wt. protein. A double-determinant assay demonstrated that MOv18 and MOv19 recognise two different epitopes on this molecule.

**Placental-type alkaline phosphatase in pre-invasive cervical neoplasia**

P.J. McLaughlin[1], G.E. Hutchinson[1], P.M. Tromans[2], A.S. Woodcock[2] & P.M. Johnson[1]

*Departments of [1]Immunology and [2]Obstetrics & Gynaecology, University of Liverpool, PO Box 147, Liverpool L69 3BX, UK.*

We studied the occurrence of placental-type alkaline phosphatase in cervical intra-epithelial neoplasia (CIN) with or without evidence of human papilloma virus infection. Three approaches were used, all utilising the monoclonal antibodies H317 and H17-E2 in enzyme immuno-assay as previously described (McLaughlin *et al.*, *Clin. Chim. Acta*, **130,** 199, 1983). The first approach, detection of the marker in sera, was uniformly negative. In contrast, 21/29 (72%) of solubilized cervical smears and 31/38 (82%) of solubilized biopsy specimens contained detectable enzyme. However, there was no correlation between placental-type alkaline phosphatase levels and degree of dysplasia or presence of HPV infection of the cervix. Nevertheless, the finding of mAb-reactive placental-type alkaline phosphatase, sometimes at high levels ($>100\,U\,kg^{-1}$ tissue) in cervical biopsy material suggests the potential for these mAbs in radio-imaging studies of metastatic or invasive cervical carcinoma.

**Purification and characterization of a tumour specific genetically engineered murine/human chimeric monoclonal antibody**

B.A. Brown, G. Davis, J. Saltzgaber-Muller, P. Simon, P.S. Shaw & G.P. Moore

*E.I. du Pont de Nemours, Co. Inc., No. Billerica, MA, USA.*

Human/murine chimeric MAbs may overcome the limitation of host response to murine MAbs in the diagnosis and treatment of human cancer. We have previously produced a human/murine chimeric MAb (cB6.2) containing the B6.2 variable region. The transfectants were introduced into mice and yielded about $200\,\mu g$ of $cB6.2\,ml^{-1}$ of ascites fluid. The cB6.2 was purified by Protein A chromatography followed by anti-murine IgG Sepharose to remove murine IgG originating from the ascites. This yielded pure cB6.2 which resembled human IgG in its reactivity with species-specific antisera and isoelectric point. In contrast, it resembled the murine parent B6.2 in its antigenic behaviour in competitive binding studies and immunofluorescent cell staining. Analysis of immunoprecipitated antigen yielded equivalent results for cB6.2 and B6.2 (70–80,000 mol. wt. protein from A549.E1 and a heterogeneous set of proteins of 97–180,000 mol. wt. from SW900, both lung lines). Finally, the two antibodies when radioiodinated behaved identically when injected into athymic mice bearing either LS174T (antigen specific) or HCT-15 (non-specific) tumours. Both MAbs imaged the specific tumours and exhibited identical localisation and clearance in the mice. With the excellent retention of antigenic binding behaviour, as demonstrated by cB6.2, similar murine/human chimeric MAbs may be a useful improvement over tumour-specific murine MAbs in the clinical setting.

**Synthesis of 1-(*p*-isothiocyanatobenzyl) DTPA. Antibody labelling and tumour imaging studies in comparison with DTPA anhydrides and EDTA**

O.A. Gansow, M. Brechbiel, R.W. Atcher, J. Scholm, D. Simpson, J. Esteban & D. Colcher

*National Cancer Institute, Bethesda, MD 20892, USA.*

To investigate the $^{111}$In-labelling tumour-localising monoclonal antibodies (MoAb), we have synthesized the chelate 1-(*p*-isothiocyanatobenzyl)-diethylenetriaminepentaacetic acid (*p*-SCN-BZ-DTPA) and its EDTA analogue. By using a MoAb (B72.3) specific for a high molecular weight antigen (TAG-72) on cells of a colorectal carcinoma grown in nude mice, optimal chemical conditions for MoAb conjugation of those ligands and the dicyclic and isobutylcarboxycarbonic anhydrides of DTPA and subsequent $^{111}$In labelling were determined. All conjugates were shown by a competitive binding assay to retain their specificity and activity *in vitro* both prior to and after $^{111}$In labelling when less than one ligand is protein coupled.

Chemical methods for purification of the MoAb were systematically investigated by injection of purified immunoprotein into athymic mice bearing LS-174T tumours which express the TAG-72 antigen. Tissue distribution studies revealed that simple addition of EDTA to labelled IgGs was ineffective at complexing indium not linked to protein by chelates. Similarly, gel chromatography (Sephadex G-50) was not sufficient, rather, size exclusion HPLC had to be employed to remove unreacted $^{111}$In and aggregated antibody.

To compare the relative utility of the 4 chelates for $^{111}$In diagnostic radioimmunoimaging, scintigraphic images of tumour bearing mice were obtained and evaluated along with tissue distributions. Results showed that clear images of these solid tissue tumours free of extraneous radiation could be obtained only by using protein coupled to *p*-SCN-BZ-DTPA and purified by HPLC after $^{111}$In labelling. Methods developed are now being modified for clinical trials for diagnosis of human colorectal cancer.

**Functionalised macrocycles – an alternative to bifunctional chelates?**

D. Parker, I. Helps & R. Morphy

*Department of Chemistry, University of Durham, South Road, Durham DH1 3LE, UK.*

Antibody-radionuclide conjugates may be used for both imaging and therapy. Radioactive metal complexes may be covalently attached to an antibody without compromising antigen binding, and the radionuclide is conventionally complexed by an acyclic chelate (EDTA, DPTA or its modified versions).

An alternative approach is to bind the metal ion within a macrocyclic ligand, which is functionalised both with ionisable groups (e.g. —$CH_2CO_2H$) to facilitate metal binding and with α-bromoacetamide or maleimide groups for coupling to an antibody. Macrocyclic metal complexes are typically both thermodynamically stable and *kinetically inert* to metal decomplexation. The design and synthesis of a series of functionalised macrocycles is reported, together with some preliminary binding studies for technetium, indium and yttrium.

**Update on $^{123}$I labelled antibodies**

M. Granowska & K.E. Britton

*Department of Nuclear Medicine, St. Bartholomew's Hospital Medical College, University of London and Imperial Cancer Research Fund Laboratories, London, UK.*

$^{123}$I is a pure gamma-ray emitting radionuclide, energy 159 KeV, which is appropriate for very efficient detection by the gamma camera for radioimmunoscintigraphy. It has a 13 h half-life which allows good serial studies for 24 h during the time of maximum uptake of monoclonal antibody which may be easily labelled using the chloramine T or iodogen technique. $^{123}$I is to be preferred to $^{123}$I because present administered doses of $^{131}$I-labelled antibodies give considerable statistical noise in the images. Our original technique using $^{123}$I-labelled monoclonal antibody of subtracting an early image from a later one has been refined through kinetic analysis of serial images with probability mapping. On the assumption that tissue background is decreasing with time and tumour uptake is increasing with time, serial images may be compared pixel by pixel and the frequency distribution of activity plotted. Using a least squares technique the clusters of pixels showing significant positive or negative deviation with time may be identified and the areas of significant change at, for example, the $P<0.001$ plotted on a map as a contour over the original data. In 23 patients 100 biopsy sites were correctly identified in a blind study as tumour positive or negative for ovarian cancer in 75% by the probability map, and all patients with metastases (some subclinical and X-ray CT negative) before second look operation and 3 out of 4 patients without metastases were correctly identified. Such identification of small recurrences, preferably without a second look operation, is a necessary prelude to successful intraperitoneal radioimmunotherapy.

### Prospective immunoscintigraphic localisation of recurrences of ovarian carcinomas using $^{131}$I-OC125 F(ab$'$)$_2$ monoclonal antibody

J.F. Chatal, J.C. Saccavini, P. Fumoleau, C. Curtet, M. Kremer, J.Y. Douillard & B. Le Mevel

*Unite 211, INSERM and CRG, Nantes, and ORIS Industrie, Saclay, France.*

OC125 monoclonal antibody recognises an antigen (CA125) associated with serous-type ovarian adenocarcinomas and shed by tumour cells into the circulation. Radioiodinated ($^{131}$I) F(ab$'$)$_2$ fragments of this antibody were injected into 24 patients previously operated on for a primary ovarian carcinoma who had increasing serum concentrations for CA125 antigen, suggesting an otherwise unsymptomatic recurrence. Each patient was injected with 74–129.5 MBq using a specific activity of 74 MBq mg$^{-1}$. A total of 34 immunoscintigraphic studies was performed in these 24 patients using planar scintigraphy and/or SPECT imaging. Two patients had a simultaneous injection of $^{131}$I-OC125 F(ab$'$)$_2$ and $^{125}$I-irrelevant immunoglobulin F(ab$'$)$_2$ 3 days before second look surgery, and radioactivity was measured in tumour and normal tissue specimens. Immunoscintigraphy correctly localised a recurrence in 18 out of 24 (75%) cases. Based on the number of tested anatomical sites, there were 23 true positive results, 13 false negative results, 50 true negative results and 2 false positive results. Ultrasonography was compared with immunoscintigraphy in 22 patients; both methods were positive in 8 confirmed tumour sites. Ultrasonography was positive and immunoscintigraphy was negative in 2 tumour sites and immunosintigraphy was positive when ultrasonography was negative in 10 tumour sites.

The percentages of injected dose per gram of tumour in the 2 patients studied were respectively 2.2 and 4.5 times higher with OC125 antibody than with irrelevant immunoglobulin. Tumour to tissue ratios ranged from 1.1 to 10.3. Immunoscintigraphy was the only positive method in 4 patients allowing curative surgical resection of the recurrence.

### Antibodies labelled with $^{111}$In and $^{90}$Y for clinical use

A.A. Epenetos

*On behalf of the Royal Postgraduate Medical School, London and Imperial Cancer Research Fund, London, UK.*

A monoclonal antibody (H17E2) against placental alkaline phsophatase (PLAP) was labelled with Indium-111 and used in radioimmunoscintigraphy of 60 patients with carcinoma of ovary, cervix, and testis. The results for this study confirmed earlier pilot studies (Epenetos *et al.*, *Lancet*, **ii**, 350, 1985) in that this method should now be incorporated in the panel of investigations used for the diagnosis, staging and monitoring of PLAP-positive neoplasms.

A new pilot study has recently commenced examining the radioimmunolocalisation potential of $^{111}$In-labelled F(ab$'$)$_2$ fragments of antibody HMFG1 in breast and non-small cell lung cancer. Results so far indicate a high degree of radioimmunolocalisation but further data are required.

A new radiolabelled $^{90}$Yttrium has been chelated to anti-PLAP antibody (H17E2) in view of the fact that $^{90}$Y may be one of the best radionuclides for tumour therapy (suitable half-life, pure beta-ray emitter of intermediate energy, stable daughter). We found that $^{90}$Y obtained from $^{90}$Sr generator is suitable for antibody labelling, achieving specific activities of 1–5 mCi $mg^{-1}$. We found no significant loss of immunoreactivity, as tested in radioimmunoassay, and no aggregate or breakdown product formation as tested by HPLC. *In vivo* biodistribution studies are currently in progress.

**Binding of IgG, IgA and IgM to cationic proteins by their Fc regions**

R.N. Poston & S. Pambakian

*Department of Histopathology, UMDS, Guy's Hospital, London SE1 9RT, UK.*

A fundamental strong interaction is described that occurs between the Fc region of all 3 major Ig classes and cationic (basic) proteins.

Experimentally, it has been studied by solid phase RIA between soluble Ig and solid phase basic proteins. It is strongest with IgM, followed by IgA and IgG. With IgG, it is greatly enhanced by aggregation. The activity is present in isolated Fc, but not in $F(ab')_2$ fragments. It is inhibitable by other serum proteins with varying efficacies, IgM binding being the least affected. This phenomemon is probably an analogue of the well-known binding of Ig to ion exchange resins; and may form the basis for many non-specific binding phenomena shown by Ig, *in vivo* and *in vitro*. (R.N. Poston, *Lancet*, **i**, 1268, 1984.)

**Monoclonal antibodies against human neuroblastoma and establishment of isotype-switch variants**

S. Schönmann, C. d'Uscio, K. Walstra & K. Blaser

*Laboratory of Molecular Immunology, University of Berne, Switzerland.*

We have established a panel of monoclonal antibodies (Mab) reacting with human neuroblastoma. By using 5 selected Mab, 3 types of neuroblastoma could be distinguished among cell lines and solid tumours according to their binding pattern. One of the Mab, designated as CE7, specifically binds to all sympatho-adrenomedullary cells. CE7 belongs to the IgG1/k class and does not mediate C′-dependent lysis of neuroblastoma cells. This was demonstrated by a $^{51}Cr$-release assay. CE7 recognizes a glycoprotein of 190 kd which binds to lentil lectin. IMR-32 neuroblastoma cells express $2\times10^5$–$5\times10^5$ CE7-binding molecules on their surface. The Mab CE7 is used for *in vitro* diagnosis.

In order to generate Mab which display the same epitope specificity but different effector functions, we have isolated spontaneously arising γ2a and γ2b isotype-switch variants from the CE7 hybridoma line. By stepwise cloning and using a sensitive isotype-specific sandwich ELISA γ2b variants were selected from the γ1 and γ2a variants from the γ2b secreting line. The frequency of arising variants in the γ1 and γ2b hybridoma lines was $5.4\times10^{-4}$ and $5.0\times10^{-4}$, respectively. Revertants were not detected. The 2 isotype switch variants retain the binding specificity for neuroblastoma cells. In contrast to CE7, the Mab AD2 being of IgM/k class and recognizing an epitope exclusively expressed on some but not all neuroblastoma cells, is cytotoxic with normal human serum or guinea pig complement. It may be used for lysis of tumour cells *in vitro*, for example purging of bone marrow from AD2-reactive neuroblastoma cells.

**Immune complexes of OC125-CA125 following intravenous injection of the radiolabelled monoclonal antibody into ovarian cancer patients**

H. Haisma, A. Battaile, R.C. Knapp & V.R. Zurawski, Jr.

*Gynaecological Oncology Laboratories, Department of Obs. & Gynae., Harvard Medical School, and Dana Farmer Cancer Institute, Boston, Mass, USA.*

The monoclonal antibody OC125 binds to ~80% of epithelial ovarian cancers and has been used to construct an immunoradiometric assay which detects the antigenic determinant, CA125, in the serum of ovarian cancer patients. Five ovarian cancer patients with pre-injection serum CA125 levels of 150–9000 U $ml^{-1}$ were injected with $^{131}I$-labelled (1.2–1.9 mCi) OC125 $F(ab')_2$ (0.5–0.8 mg) labelled at a specific activity of 2.5 μCi $\mu g^{-1}$. Using gel filtration of the patients' sera, apparent immune complexes were observed within 15 min after injection of the monoclonal antibody. By 5 days after injection 0.25% of the OC125 antibody could be detected in its native form. The rate of immune complex formation and the amount of complexes formed correlated well with the observed pre-injection serum CA125 level. *In vitro* experiments confirmed the correlation between complex formation and CA125 level. We conclude, therefore, that the complexes consist of CA125-OC125 immune complex. Despite the presence of serum immune complexes, $^{131}I$ labelled monoclonal OC125 could be found localised in tumour tissue at post-surgical examination by gamma counting.

Tumour to surrounding tissue ratios were in excess of 10–20. However, tumour to blood ratios never exceeded 2. These findings were corroborated by immunoperoxidase staining techniques.

Our study suggests that the presence of immune complexes after injection of a monoclonal antibody into cancer patients will have an impact on the use of monoclonal antibody OC125 and other monoclonal antibodies reactive with serum antigens for radioimmunodetection and for therapy.

### Multicentre studies of immunoscintigraphy of the Italian National Research Council

A.G. Siccardi

*Dipartimento di Biologia e Genetica per le Scienze Mediche, Universita Degli Studi di Milano, 20133 Milan, Italy.*

The restricted tissue distribution of tumour-associated antigens and the high degree of specificity of the corresponding monoclonal antibodies have been exploited for immunoradioimaging procedures to visualise lesions in patients with solid tumours. A multicentre study was performed to analyse the efficacy of $^{99m}$Tc- and $^{111}$In-labelled $F(ab')_2$ fragments of monoclonal antibody 225.28S (reactive with HMW-MAA, a high molecular weight melanoma associated antigen) to radioimage malignant lesions in patients with melanoma. A total of 254 patients with melanoma, carrying 412 documented lesions, were studied in 10 centres. A total of 377 lesions were visualised in 206 patients; in particular, (i) 250/412 known lesions were visualised in 151/191 patients known to have melanoma lesions; (ii) 95 occult lesions were visualised in 61 patients of the same group; (iii) 32 lesions were visualised in 15/63 patients without diagnosed lesions. The melanoma nature of 101/127 radioimaged occult lesions was confirmed by clinical criteria and/or by additional laboratory investigations. The study has shown good agreement in the results obtained by the 10 centres, suggesting that immunoscintigraphy with radiolabelled $F(ab')_2$ fragments of the MoAb 225.28S is a reliable procedure. In order to confirm the efficacy of the immunoscintigraphic method, another multicentre study, concerning 'Immunoscintigraphy for detection of metastases of colerectal carcinoma through radiolabelled anti-CEA monoclonal antibodies' has been started recently.

### Immunolymphoscintigraphy in breast cancer patients

N. Pateisky[1], K. Philipp[1], C. Schatten[1] & R. Mandeville[2]

[1] *1st Department of Obstetrics & Gynaecology, University of Vienna, A-1090 Vienna, Austria and* [2] *Institut Armand-Frappier, University of Quebec, Canada J7E 4B9.*

Subcutaneous application of radiolabelled antibody seemed to us to be an appropriate way to examine metastatic involvement of the lymphatics draining a primary carcinoma. Using 3 different tumour-associated monoclonal antibodies (known to react strongly with breast carcinoma cells *in vitro*), we investigated the axillary regions of 35 patients suspected of having breast cancer. Radioactive I-123 was used as the label. The finger webs between the second and third fingers served as injection sites. Static images were taken up to 8 h after the application. Activity accumulation over the axilla was used as an indicator for metastatic involvement. In each of the patients antibodies were injected on both sides. The difference in activity accumulation (counts) over the axillary regions was calculated. If the difference was statistically significant, the scan was judged as positive. The antibody 3C6F9 appeared to be the best. Out of 20 patients the results were correct in 18 ($6\times$negative, $12\times$positive). The scan results were compared with the histology of the surgically removed nodes and data so far would indicate that the method of immunolymphoscintigraphy may be of value in staging patients with breast cancer.

### Tumour imaging with $^{111}$In and $^{131}$I labelled anti-CEA antibodies (BW 431/31)

J. Vanderick[1], N. Leners[2], A. Ferrant[2], C. Deckers[1] & G. Schulz[3]

[1] *Exper. Cancer,* [2] *Nucl. Med., University of Louvain Medical School, Brussels, Belgium and* [3] *Res. Lab., Behringwerke AG, Marburg 1, FRG.*

The murine anti-CEA monoclonal antibody BW 431/31 (Bosslet *et al., Int. J. Cancer,* **36,** 75, 1985) has been used in 10 patients. Eight had colorectal carcinoma and 2 had elevated CEA of unknown origin. The first 7 patients had injection of 0.5 mg monoclonal antibody (Mab), and the 3 others received 3 mg. In 6 patients, intact Mab was given and in 4 $F(ab')_2$ fragments. BW 431/31 was labelled with $^{111}$In in 8 patients and with $^{131}$I in 2 patients. Scintigrams were performed 24, 48, 72 and, in 3 patients, 96 h after injection. In 3 out of 8 patients with colorectal carcinoma, the findings of radioimmunoscintigraphy and CT scan were in agreement. In 3 other patients, in apparent clinical remission, sites of abnormal uptake were seen. Two patients had bulky necrotic masses. No uptake was observed in one and a slight uptake in the other. In 2 patients with elevated CEA of unknown origin, the radioimmunoscintigrams were negative. No side-effects were observed after injection of the Mabs. In 3 patients, activity was observed at the site of the kidneys after injection of labelled $F(ab')_2$ fragments. Radioimmunoscintigraphy using BW 431/31 may give correct information on the localisation of colorectal carcinoma. Adequate uptake can be observed from 24 h after injection. $F(ab')_2$ fragments provided better defined images than those obtained with intact Mab and the quality was further enhanced with $^{111}$In labelling. Further work is needed to show if there is an advantage in using 3 mg instead of 0.5 mg Mab.

**The effect of increasing unlabelled monoclonal antibody (MoAb) doses on metastases detection and on body distribution of various $^{111}$In MoAbs**

L.M. Lamki[1], T.P. Haynie[1], J.L. Murray[1], R. Babaian[1], Y.Z. Patt[1] & B. Merchant[2]

[1]*Department of Nuclear Medicine, MD Anderson Hospital, Houston TX 77030 and* [2]*Hybritech Inc., CA, USA.*

We have studied 4 different murine MoAbs, all labelled with 5 mCi of $^{111}$In. These include 96.5 (anti-P97, melanoma), ZME-018 (anti-high molecular weight antigen of melanoma), ZCE-025 (anti-CEA) and PAY-276 (anti-prostatic acid phosphatase). Twenty to 25 patients were studied using each MoAb. In each study, patients were divided into groups of 5 patients according to the amount of unlabelled MoAb injected with the 1 mg $^{111}$In-labelled MoAb. Unlabelled MoAb dose varied from 1 mg to 80 mg. Images, digital and analogue, were acquired at 24, 72 and in most cases at 120 and 168 h. Lesion detection was compared with clinical, radiographic and other scintigraphic results. Regions of interest were used to analyse relative non-tumour body distribution of labelled MoAb at 72 h.

In all 4 MoAb results, detection rate of metastatic lesions improved with increasing MoAb dose but the 'saturation' point differed with each of them, ranging from 20 to 80 mg.

The non-tumour body distribution also varied with increasing MoAb dose. The liver uptake fell as the bone, kidney and blood pool increased with increasing MoAb dose. The level at which statistical significance occurred, however, varied with each antibody. Spleen uptake was variable.

The 'blocking' effect of unlabelled MoAb influences the non-specific distribution of labelled MoAb primarily through reduction of liver uptake and increases the sensitivity of metastases detection.

**Anti-tumour effects of immunotoxins *in vitro* and *in vivo***

P.E. Thorpe, E.J. Wawrzynczak, D.C. Blakey & F. Stirpe

*Imperial Cancer Research Fund, Lincoln's Inn Fields, London WC2, UK.*

A panel of immunotoxins was prepared by linking monoclonal anti-Thy1.1 antibody with the SPDP reagent to the A-chains of ricin or abrin or to 3 ribosome-inactivating proteins which act in analogous fashion: saporin, bryodin and Momordica charantia inhibitor (MCI).

*Cytotoxicity.* The immunotoxins were specifically toxic to Thy1.1-espressing AKR-A lymphoma cells in tissue culture. The concentrations of immunotoxin that reduced protein synthesis by 50% ranged between $10^{-11}$ M for the abrin A immunotoxin and $10^{-9}$ M for the MCI immunotoxin.

*Antitumour activity.* Mice bearing peritoneal AKR-A lymphoma cells were given a single i.v. injection of 0.3 nmol of immunotoxin. The extension in median survival time ranged from 6 days for the abrin A immunotoxin (corresponding to 99% tumour cell kill) to 24 days for the saporin and bryodin immunotoxins (corresponding to 99.999% tumour cell kill).

*Linkage.* The low antitumour activity of the abrin A immunotoxin could be rectified by changing the crosslinker from SPDP to 2-iminothiolane. This is because ~1% of AKR-A cells survive exposure to the SPDP-linked immunotoxin (both *in vitro* and *in vivo*) but are killed by the 2-iminothiolane-linked immunotoxin. It is possible that the resistant cells have elevated levels of an enzyme capable of splitting the amide bond in the SPDP linkage. Both the SPDP and 2-iminothiolane linkages break down slowly *in vivo* ($T\frac{1}{2}=8$ h), probably because the disulphide bond is unstable.

A new coupling agent, SMBT, has been made which avoids this problem.

*Reticuloendothelial recognition.* Ricin A immunotoxins are cleared *in vivo* by the reticuloendothelial cells of the liver. These cells have receptors for the oligosaccharide portion of ricin A-chain. Deglycosylation of the A-chain with metaperiodate and cyanoborohydride prevents clearance of the immunotoxin by the reticuloendothelial system.

*Pathology.* Linkage of saporin and abrin A-chain to immunoglobulin increases their toxicity to mice by 10–20 fold. Both immunotoxins cause extensive hepatic necrosis whereas neither saporin nor abrin A-chain in unconjugated form cause significant liver damage.

### Drug-antibody conjugates for cancer therapy

R.W. Baldwin

*Cancer Research Campaign Laboratories, University of Nottingham, Notts. NG7 2RD, UK.*

Conjugation of cytotoxic drugs to antibody aims to introduce the maximum number of residues whilst retaining acceptable retention of both drug and antibody reactivities. This will be considered with respect to the design of conjugates in which monoclonal antibody 791T/36 has been directly linked to a range of anti-cancer agents including methotrexate, daunomycin and vindesine; drug activity being evaluated by *in vitro* cytotoxicity assays and antibody by a flow cytometry assay developed to precisely define the antibody potency in conjugates.

In general only a limited number of drug residues can be linked to antibody and in order to increase the drug-antibody combining ratio drug-carrier systems have been developed. This will be considered with respect to the design of methotrexate-human serum albumin 791T/36 monoclonal antibody conjugates. This has increased the drug:antibody ratio to 30–40:1, so increasing the potency of the conjugates.

Therapy trials with MTX conjugated to monoclonal antibody 791T/36 have shown that they suppress growth of human tumour xenografts in immunodeprived mice. Furthermore, the antibody binds to target cells derived from primary and metastatic colorectal carcinomas. These studies will be considered with respect to be design of clinical trials with methotrexate conjugates.

### Antibody-kemptide conjugate: A novel method for the $^{32}P$ labelling of monoclonal antibodies

B.M.J. Foxwell, H. Bond, G. Watson, D. Snook, J. Long, W. Jeffery, P. Parker, A.A. Epenetos, P. Thorpe & A. Creighton

*Imperial Cancer Research Fund, Lincoln's Inn Fields, London WC2 and Royal Postgraduate Medical School, London W12, UK.*

In recent years, radiolabelled monoclonal antibodies have been evaluated for their use in the diagnosis and treatment of neoplastic disease. One isotope which has not been assessed for antibody targeting is $^{32}P$, even though this isotope has many favourable radiobiological characters and has been used clinically for the treatment of certain neoplastic disorders. The main drawback so far in using $^{32}P$ has been the absence of a general method for phosphorylating antibodies. We have now developed a novel process for the phosphorylation of immunoglobulins which is rapid, efficient and allows for high specific activities to be achieved ($>10\,\mu Ci\,\mu g^{-1}$). The technique involves the chemical conjugation of Kemptide, a synthetic septapeptide substrate for kinases, to immunoglobulins. The antibody-kemptide conjugate is then able to act as a substrate for protein kinases. The conjugation procedure does not compromise the binding activity of the antibody. Studies have shown that $^{32}P$-labelled monoclonal antibodies are stable in human, mouse and rat plasmas *in vitro*. We are now assessing the *in vivo* clearance of kemptide-antibody conjugates and the efficacy of using $^{32}P$ as an antibody-targeted isotope.

### Potential value of intrahepatic $^{131}I$-ethiodol in patients with hepatocellular carcinoma

C.H. Park[1], J.H. Suh[2], H.S. Yoo[2], J.T. Lee[2] & D.I. Kim[2]

[1]*Division of Nuclear Medicine, Thomas Jefferson University Hospital, Philadelphia, USA and* [2]*Department of Radiology, Yan-Sei University Hospital, Seoul, Korea.*

The specific aim of the study was to evaluate therapeutic feasibility of radiolabelled iodized oil injected into the hepatic artery proper on patients with hepatocellular carcinoma (HCC).

In a recent study, Ethiodol (or Lipiodol), which is an iodized poppy seed oil, was selectivity retained in the tumour vessels of large HCC in the liver for long periods following injection into the hepatic artery.

Recently, we replaced with $^{131}I$ a small quantity (pg) of the stable iodine of the 37% iodine by weight in Ethiodol with 100% exchange efficiency.

Patients with HCC were injected with $^{131}I$-Ethiodol through the hepatic artery. The agent was stable for 8 days *in vivo* and there was no significant activity in the thyroid gland, stomach, lung. Urine and blood activity was negligible. There was a high tumour to normal liver ratio for up to 8 days post-injection indicating that $^{131}I$-Ethiodol will be effective delivering high internal radiation dose to the HCC.

### Activation of human complement by covalent conjugates of mouse monoclonal antibodies and cobra venom factors

B. Müller, H. Harpprecht, M.J.D. Anderson & W. Müller-Ruchholtz

*Abt. Immunologie, Universitat Kiel, D-2300 Kiel, FRG.*

For T-cell depletion in allogeneic bone marrow transplantation it is highly desirable to use antibodies or, rather, antibody conjugates which activate human complement (C), since unspecific toxicity of rabbit sera against human stem cells is usually high

*Methods*: Two different monoclonal antibodies (MoAb; KI36 directed against a monomorphic HLA DR determinant and K39A6 directed against a determinant on human lymphocytes) were conjugated to cobra venom factor (CVF) using the heterobifunctional reagent SPDP. The cytotoxic potential of the non-modified MoAbs and the conjugates against human leukaemia cell lines and lymphocytes from different organs was estimated in a $^{51}$-Chromium release assay, comparing rabbit and human sera as source of C.

*Results*: (1) While KI36 does not fix C, the conjugate KI36/CVF lyses specifically cells which express HLA DR. (2) K39A6 lyses lymphocytes in presence of rabbit C, but is ineffective with human C at any concentration tested. (3) The conjugate K39A6/CVF activates rabbit C at least as well as K39A6 alone. Furthermore, it initiates very efficiently human C dependent cytolysis. (4) CVF-initiated cytolysis, using the alternative pathway of C activation, is slower than Ab-initiated lysis via the classical pathway and reaches maximal killing of target cells after 4 h. These data show that it is possible to use mouse Ab for killing human lymphocytes with autologous C by bonding it covalently to CVF.

### Selective insertion of $^{32}P$ into immunoglobulins by protein kinases

F.R. Burnet[1], C.G. Proud[1], T. Williams[1], A. Richardson[2], E. Heyderman[3], P. Mountford[2] & A. Coakley[2]

[1]*Biological Laboratory, University of Kent at Canterbury,* [2]*Department of Nuclear Medicine, Kent & Canterbury Hospital,* [3]*Department of Histopathology, St. Thomas's Hospital, London SE1, UK.*

Radiolabelled tumour directed immunoglobulins have great potential as a means of both locating and treating solid tumours and their metastases. The radiochemical properties of $^{32}P$ make it a good candidate as a therapeutically useful isotope with which to tag immunoglobulins. We have tested the ability of cyclic AMP dependent protein kinase (cAMP dep PK), Casein Kinase II and Protein Kinase C to phosphorylate both a pan-reactive anti-$\beta$ tubulin mouse monoclonal (IgG, type 2B, KMX-1) and a selection of polyclonal IgG fractions from different animals. One of them was rabbit anti-prostatic acid phosphatase (anti-PAP) which is undergoing clinical evaluation in the imaging of the metastases of prostate tumours. None of the kinases were capable of inserting $^{32}P$ from [$\gamma$-$^{32}P$]ATP into the native immunoglobulins, but cAMP dep PK was found to be capable of phosphorylating all of the immunoglobulins tested after their partial denaturation in urea. This denaturation was found to be reversible. Stoichiometry of $^{32}P$ mol inserted per mol IgG ranged from 0.1 for KMX-1 to 1 for anti-PAP. We are now extending our studies to discover the optimal denaturation condition for phosphorylation and the factors which determine this denaturation's reversibility in immunoglobulins phosphorylated in different domains.

### Immunoscintigram with monoclonal antibodies of liver metastases of colorectal cancer

R.G.E. Holzheimer, K.H. Muhrer, H. Müller & K. Bosslet

*Departments of General Surgery and Nuclear Medicine, Justus-Liebig-Universitat, Giessen and Behringwerke AG, D-3550, Marburg 1, FRG.*

In a pretherapeutic study we examined the value of intra-arterial infusion of CEA-specific monoclonal antibody in 13 patients with liver metastases from colorectal cancer. In these patients a catheter was implanted in the hepatic artery for regional chemotherapy. Monoclonal antibody (Tu MAK BW 431/31) binds specifically to CEA. The antibody was labelled with $^{131}I$. During the 1 h infusion, we observed and documented the distribution kinetics of the monoclonal antibody. We performed a control scintigram after 24 h, 2 and 3 days. Localisation and distribution of the metastases were verified by CT scan. In 9 of 13 patients we could see distinctive enhancement of activity in metastatic lesions during the 1 h intra-arterial infusion. These enhancements resembled defects in the $^{99}Tc$-colloid scintigram. The control scintigram showed less enhancement at 24 or 48 h after infusion of activity but by 72 h there was again increased uptake in the metastases. The positive visualisation of metastases after 1 h infusion lets us conclude that by intra-arterial infusion this monoclonal antibody binds quickly to the tumour.

### Immunoscintigraphy of lung carcinoma: Experience in 45 patients

P. Riva, G. Paganelli, G. Riciputi, G. Pasini, S. Benini, M. Agostini, G. Moscatelli, G. Cacciaguerra, V. Tison, P. Morigi & V. Stancanelli

*Istituto Oncologico Romagnolo, 47023 Cesena, Italy.*

The most widely studied tumours employing monoclonal antibodies (MoAb) are those of the gastrointestinal tract, skin (mainly melanoma) and female genital tract. *In vivo* studies in patients with lung cancer are very few. We decided to carry out an *in vivo* phase I study, since we found positive staining in 80% of tissue sections from lung cancer using the MoAb FO23C5 against CEA. No positive results were obtained either *in vitro* and *in vivo* when we used, as negative control, a non-specific MoAb raised against the HMW MAA (melanoma-associated antigen).

Thirty-three patients with squamous carcinoma, 8 with oat cell carcinoma and 4 with chronic pulmonary disease were studied by means of $^{111}In$ and/or $^{131}I$-labelled $F(ab')_2$ fragments of the above-mentioned MoAb.

The antibody scan visualised 27 out of 31 primary tumours (87%) and 25 out of 30 documented metastatic lesions. No positive scan was obtained in any patient with non-neoplastic pulmonary disease.

In addition, we succeeded in detecting occult metastases not diagnosed by conventional radiology. Best images were obtained with $^{111}In$-labelled MoAb at 72 h after i.v. injection. The tumour to background ratio obtained *in vivo* by means of ROI technique, and *in vitro*, employing a multichannel system, was better when $^{111}In$ label was used and ranged between 2.3 to 4.7.

### Intra-tumoral distribution of labelled antibodies and fragments. Autoradiography of tumour-bearing nude mice

S. Matzku, B. Gamer & W. Tilgen

*Institute of Nuclear Medicine, Deutsches Krebsforschungszentrum and Department of Dermatology, University of Heidelberg, FRG.*

To get access into solid tissue, antibody molecules have to cross the endothelial lining and the basement membrane of peripheral vessels, followed by translocation in the extravascular space. In the tumour tissue, the blood-tissue barrier may be leaky to a variable degree, thus providing a surplus of antibody influx. Yet, strategies to improve access are badly needed. To characterise changes in antibody penetration into tumour tissue, we performed whole body autoradiography of mice with melanoma transplants, systematically looking at the following variables: morphotype of melanoma line, intra-tumoral blood volume, penetration of irrelevant MAb, penetration of various anti-melanoma MAbs, accumulation time, fragment size (i.e. intact IgG, $F(ab')_2$, Fab).

It was observed that all tumour lines showed a surprisingly poor blood supply and diffuse distribution of irrelevant IgG. With MAbs known to exhibit significant uptake in the paired label assay, a highly inhomogeneous activity distribution was obvious in all tumours, activity being deposited in close vicinity to supplying blood vessels. With both types of fragments, whole body autoradiography confirmed the well known fact of rapid clearance of radioiodinated species from the organism, and it revealed an obvious increase in the permeation of radioactivity throughout the tumour node, thus reaching areas of low blood input, too.

### The effect of second antibody in the radioimmunotherapy of colorectal cancer

J.A. Ledermann, R.H.J. Begent, S.R. Riggs, P. Keep, F. Searle, A.J. Green, M.G. Glaser, R.J. Dale & K.D. Bagshawe

*CRC Laboratories, Department of Medical Oncology, Charing Cross Hospital, London W6 8RF, UK.*

We have used a 'second antibody' directed against the radiolabelled anti-tumour antibody in order to establish if the background radioactivity and therefore potential toxicity to normal tissues can be reduced. Six patients with advanced colorectal carcinoma were given 40–55 mCi $^{131}I$ labelled sheep anti-CEA (2.5 mg) and 3 patients received 'second antibody' donkey anti-sheep (10 mg) 24 h later. Radioactivity cleared more rapidly in those receiving 'second antibody', the majority appearing in the urine and <1% in the faeces. Measurements of the dose received by the tumour and normal tissues were made to assess whether the accelerated clearance of anti-tumour antibody could enhance the therapeutic ratio.

In total 11 patients have been treated with either goat or sheep anti-CEA. There were few side-effects: 7/11 developed transient chills and fever; no cases of myelosuppression, impaired renal or hepatic function were seen. Using ELISA human anti-goat antibody was demonstrated prior to treatment in 2 patients and developed in a further 6. Antibodies were not detected in those patients who did not receive 'second antibody'. The tumour could be visualised by scintigraphy in 10 patients and was histologically confirmed in 4/5. An objective response was seen in 2 patients; a fall in CEA and CA19-19 levlels and improvement in pain for one month in one and a reduction in the size of an hepatic metastasis on CT in another. A further 2 patients had an improvement in pain. These results demonstrate the potential benefit of second antibody and the safety of this dose level. However, immunisation of the host against antibodies is a significant problem and may limit the potential for repeated therapy.

### Dosimetry of radiolabelled antibodies

P.K. Leichner

*The Johns Hopkins Oncology Centre, Baltimore, Maryland 21205, USA.*

Optimisation of radiolabelled antibody cancer therapy in clinical trials depends on dosimetry for radiation dose estimates, choice of antibodies and activities to be administered and the timing of administrations. The necessary data are acquired by quantitative imaging and image analysis of X-ray CT and SPECT scans as well as planar gamma camera images. Semi-automatic computer software has been developed to compute tumour and normal organ volumes from CT and SPECT scans and for the *in vivo* quantitation of radiolabelled antibody activities in these volumes from conjugate gamma camera views. Additionally, computerised volumetrics are used to assess tumour response to therapy. Comparison with autopsy data has shown that computerised CT volumetrics of primary hepatic cancers are an accurate (±6%) and reproducible way to determine volumes non-invasively. Phantom studies indicate that the accuracy of *in vivo* quantification of $^{111}In$ activities ranges from 0.9 to 9%, depending on volumes and activities being imaged.

This methodology is being used clinically in an ongoing Phase 1-2 trial of radiolabelled antiferritin IgG in hepatoma. $^{111}In$-labelled antiferritin is used as the imaging agent to calculate $^{90}Y$ antiferritin activities to be administered to achieve predetermined tumour absorbed doses and at the same time reduce toxicity to normal tissues. Labelling ratios of $^{90}Y$ antiferritin have ranged from 10–40 mCi mg$^{-1}$ and administered activities from 8–37 mCi. Tumour dose rates of up to 38 cGy h$^{-1}$ have been achieved after external-beam tumour irradiation of 600 cGy or 900 cGy.

### Regional antibody therapy

A.A. Epenetos

*On behalf of Royal Postgraduate Medical School, London and Imperial Cancer Research Fund, London, UK.*

Following the demonstration (Epenetos *et al., Lancet,* **i**, 1441, 1984) that monoclonal antibody targeted radiotherapy may prove a valuable form of treatment in certain situations, at least when it is delivered into body cavities and regions rather than intravenously, we expanded our studies in various areas.

1. A randomised multicentre clinical trial using radioactively labelled monoclonal antibodies for the treatment of stage III minimal residual disease ovarian cancer has recently been initiated, following the completion of a phase I-II study where data were obtained regarding toxicity and response.

2. Following the completion of a trial involving intracavity administration of antibodies in the treatment of serous effusion (12 responders out of 15 patients), a randomised study has commenced of $^{131}$I-labelled specific *vs.* non-specific antibody administered on an out-patient basis.

3. Six patients with grade III/IV glioma of brain having relapsed after radiotherapy $\pm$ chemotherapy have received intra-arterial infusions of $^{131}$I-labelled monoclonal antibody (EGFR1, against epidermal growth factor receptor) as a therapeutic attempt. Four out of 6 patients responded favourably with clinical and radiological improvement.

4. Monoclonal antibodies to carcinoembryonic antigen (CEA) were radiolabelled with $^{131}$I and administered intra-arterially (via the hepatic artery) concurrently with biodegradable starch microspheres in order to enhance tumour uptake of antibody and achieve a therapeutic response. Favourable results were observed in one patient treated thus far. A pilot study has recently commenced examining the potential therapeutic effect of this approach in patients with small size (<2 cm diam.) liver metastases.

### Analysis of the human anti-mouse Ig response in patients receiving murine monoclonal antibodies

M.A. Ritter, N.S. Courtenay-Luck, M. Larché, B. Dhokia, G. Sivolapenko & A.A. Epenetos

*Departments of Immunology and Clinical Oncology, Royal Postgraduate Medical School, London, UK.*

The use of mouse monoclonal antibodies in the diagnosis and therapy of malignant neoplasms is of considerable clinical interest. However, a consequence of such treatment is the stimulation of a human immune response to the injected mouse immunoglobulins. Prolonged monoclonal antibody treatment leads to an elevation of this response, resulting in an increased risk of Type III immune complex mediated hypersensitivity. An understanding of the underlying immune mechanisms involved in the development of the human anti-mouse Ig response is clearly of importance if such side effects are to be avoided.

We have studied the anti-mouse Ig response in ovarian carcinoma patients who have received both diagnostic and therapeutic injections of $^{131}$I-labelled mouse monoclonal antibodies. The following parameters have been analysed:

(*a*) pre-existing antimouse Ig antibodies, prior to treatment;

(*b*) location of the antigenic determinants on mouse Ig to which the response is directed;

(*c*) the prozone effect obtained with high titre sera and its implications in routine clinical assays;

(*d*) the development of anti-tumour antibodies during monoclonal antibody treatment;

(*e*) the possible risk of autoimmune reactions.

### Primary ocular melanoma: Evaluation with radioimmunoscintigraphy

K. Scheidhauer, F. Stefani, A. Markl, U. Schumacher & E. Moser

*Departments of Radiology, Ophthalmology and Anatomy, University of Munich, 8000 Munich 70, FRG.*

Radioimmunoscintigraphy of cutaneous malignant melanoma has been reported in several clinical trials, mostly in patients with multiple metastases and extended lesions. This study was undertaken to evaluate the potential of radioimmunoscintigraphy in small lesions.

Therefore, 9 patients suffering from primary ocular melanoma were investigated. The average of lesions' prominence was 6.7 mm (range: 3.5–10.8 mm), proved by opthalmoscopy and ultrasound. RIS was performed 18 h post-injection of $^{99m}$Tc-labelled F(ab$'$)$_2$ fragments (350 $\mu$g) of an IgG$_{2a}$ monoclonal antibody (225.28S) – developed by Ferrone *et al.* and supplied as a ready-to-use kit (Sorin, Italy). Scintigrams were achieved in the planar manner as well as by emission computed tomography (ECT). In addition, 3/9 patients were investigated by transmission CT and magnetic resonance imaging (MRI). Three ocular melanomas were treated by enucleation with subsequent immunohistochemistry, 5 were treated by radiation therapy with a 106-Ruthenium plaque.

Despite the small size of the lesions, the scintigraphic results were encouraging. 8/9 lesions were visualised; this was possible by ECT only, whereas planar scintigraphy failed to detect 4/8 ocular melanoma. The presence of the recognised high-molecular-weight melanoma-associated antigen could be documented by immunohistochemistry.

The image quality confirmed that radioimmunoscintigraphy with ECT was able to detect lesions smaller than 1 cm. Unspecific uptake in RES and kidneys, however, was a limiting factor of this antibody in other locations.

### Correlation study between tumour antigenic expression and *in vivo* binding of radiolabelled antibodies

J.Y. Douillard, P.A. Lehur, M. Kremer, G. Aillet, J.F. Chatal & A. Bianco-Arco

*Chirurgie Generale et Digestive, CHU Nord, INSERM Unite 211 and Centre Rene Gauducheau, Nantes, France.*

In order to investigate if the amount of tumour-specific monoclonal antibodies targeted to tumour cells after *in vivo* infusion was dependent in part upon the amount of antigenic expression at the tumour site, a correlation study was undertaken comparing quantitative binding of radiolabelled antibodies to quantitative antigenic expression. $^{125}$I anti-CEA and $^{131}$I anti-Ca 19-9 were infused 3 days in 6 patients and 4 days in one patient before radical surgery for colorectal carcinoma. Three tumour specimens were obtained during surgery along with normal colon, fat, muscle, skin, liver and blood sample. All specimens were evaluated for gamma emission, expressing results as % injected dose per gram of tissue. After counting, specimens were assayed using avidin biotin peroxidase complex to evaluate antigenic expression. Results were scored from 0 to 100 taking into account both the percentage of stained cells and the intensity of staining.

Correlation studied for these 7 patients was excellent for both antibodies. CEA *in vivo* binding correlated to tissue antigenic expression with an $r$ value of 0.69 to 0.90 for each pathologist respectively. The $r$ value of inter-observer correlation was 0.74. Similar good correlation was observed for anti-Ca 19-9 antibody targeting and Ca 19-9 expression with $r$ values of 0.78 and 0.84 and inter-observer $r$ of 0.97. Results of this limited study confirm the rationale for using tumour-specific antibodies for cancer imaging. Ability of a given tumour to be imaged by radiolabelled antibodies is, however, only in part dependent upon antigenic expression, since other factors like blood flow, vascularisation, have also to be considered for accessibility of antibodies to tumour-associated antigens.

### Specific radioimmunotherapy with Yttrium-90 monoclonal antibody

W.T. Anderson-Berg, C.L. Ruegg & M. Strand

*Department of Pharmacology and Experimental Therapeutics, The Johns Hopkins University School of Medicine, Baltimore, MD 21205, USA.*

The specificity, efficacy and toxicity of radioimmunotherapy with radioiodinated monoclonal antibody (MAb) was compared with $^{90}$Y-labelled DTPA-derivated MAb in an animal model system. Although tumour mass was reduced following treatment with $^{131}$I-labelled immunoglobulin (IgG) (ED50 = 50 $\mu$Ci), specific and control IgGs had equal potency attributable to whole body irradiation. In contrast, with $^{90}$Y, specific MAb was threefold more potent than control IgG (ED50 = 9 and 25 $\mu$Ci respectively). Histologically, microscopic tumour foci were eliminated only in animals treated with specific MAb. Further, myelosuppression was dose-limiting with iodine but, with yttrium, was observed only at doses several-fold greater than the therapeutically effective dose.

The enhanced specificity and decreased toxicity observed with yttrium-labelled antibody can be ascribed to both physical and biological factors. The longer physical half-life and long range gamma emissions of $^{131}$I increase its toxicity. Further, at the tumour, iodinated IgG was rapidly dehalogenated with a $T_{\frac{1}{2}}$ of 2–3 h, whereas yttrium remains stably chelated and $^{90}$Y-labelled MAb continued to accumulate at the tumour target over 5 days. Based on these results, $^{90}$Y-labelled antibody appears to be a promising agent for clinical radioimmunotherapy.

### Heteroantibody duplexes target tumour cells for lysis by human cytotoxic T lymphocytes

M.A. Liu[1,2], B.-A. Khaw[2], H.W. Strauss[2] & N. Eisen[1]

[1]*Massachusetts Institute of Technology, Cambridge, MA 02139 and* [2]*Massachusetts General Hospital, Boston, MA 02114, USA.*

Cytotoxic T cells (Tc cells) exhibit a high degree of specificity for their target cells. However, when anti-idiotypic antibodies directed against the T cell antigen-specific $\alpha\beta$ heterodimeric receptor have been attached to diverse tumour cells, a cytotoxic T cell clone effectively and specifically kills the tumour cells regardless of the tumour's indigenous surface antigens. We sought to develop a general means for targeting tumour cells for lysis by any Tc cell independent of the Tc cell's antigen specificity.

We used heteroantibody duplexes composed of an antibody to T3 (which is intimately associated with the $\alpha\beta$ receptors on all human mature T cells)

covalently joined to a second antibody specific for an antigen on the intended human tumour target cell. In the presence of the anti-T3/anti-tumour heteroantibody duplex the tumour cells were lysed by a clone of human T8+ Tc cells of unrelated specificity but not by a non-cytotoxic clone of human T4+ helper T cells. Lysis by the Tc cells was specifically blocked by the uncoupled anti-T3 or the uncoupled anti-tumour antibodies (Liu *et al.*, *Proc. Natl Acad. Sci.*, **82,** 8648, 1985). *In vivo* studies with radiolabelled heteroantibodies and Tc cells in nude mice with solid tumours are in progress. The usage of an anti-T3 antibody facilitates the possible application of this technique for immunotherapy by obviating the development of multiple anti-idiotypic receptor antibodies. Overall, our findings suggest that heteroantibody duplexes containing anti-T3 antibody may be capable of targeting selected cells, such as tumour cells, for destruction *in vivo* by the body's cytotoxic T cells.

### Limitations to tumour killing using radiolabelled antibodies

A.T.M. Vaughan, A.R. Bradwell, P. Anderson & P.W. Dykes

*Department of Immunology, University of Birmingham, Birmingham B15 2TJ, UK.*

Using available data on the distribution of radiolabelled antibodies in man, we have made calculations to illustrate the conditions required for tumour destruction following a single i.v. injection of labelled antibody. The calculations are based on a model containing the following variables. The rate of uptake and loss of activity by the tumour, the rate of elimination of activity from the whole body and the maximum percentage uptake of activity by the tumour. We have set as a target the delivery of 60 Gy of radiation to the tumour in one week, following successful radiotherapy practice. The conclusions to be drawn are clear. Using either Iodine-131 or Yttrium-90 as an antibody label, tumour destruction is associated with lethal whole body irradiation. A minimum 10-fold improvement is required in specific uptake of radioactivity into the tumour to make therapy a practical proposition. For all conditions studied, however, Yttrium-90 appears the isotope of choice, due to its lack of any gamma emission and its more energetic beta decay. Successful therapy may result from either increasing the specific tumour content of radioactivity, or the extraction of radioactivity from sites external to the tumour. The latter can be achieved by direct immunological manipulation, using an antibody directed against the first to clear the blood pool, or the application of methods utilising the particular chemistry of the isotope label. We have investigated two chemical approaches, Yttrium-90 may be non-specifically removed from the body by administration of soluble chelates. More usefully, Gold-199 bound to antibodies is eliminated from the body as it is cleared from the blood but, like Yttrium-90, is retained in extravascular locations.

### Intraperitoneal versus intravenous injection of radiolabelled monoclonal antibodies in patients with colorectal carcinoma

G. Paganelli, P. Riva, G. Sarti, V. Tison, G. Cacciaguerra, G. Fiorentini, G. Moscatelli, S. Benini & M. Agostini

*Istituto Oncologico Romagnolo, 47023 Cesena, Italy.*

The anti-CEA MoAb (clone FO23C5-Sorin Biomedica) employed for this study belongs to the $IgG_1$ class and is directed against the CEA peptide chain. It does not react significantly with cross-reacting antigen (NCA) and with normal colon mucosa extracts.

Sixty patients with colorectal carcinoma were injected i.v. with 2–3 mCi of $^{131}$I-labelled $F(ab')_2$ fragments corresponding to 300–600 $\mu$g of proteins, obtaining positive results in the 80% of documented lesions. No positive results were obtained both *in vivo* and *in vitro* when we used a negative control antibody raised against the HMW-MAA, melanoma antigen.

However, the major drawback was the low sensitivity in liver metastases (59%) and the poor tumour to background ratio achievable. Twenty of these patients were injected intraperitoneally with improved results in the detection of liver metastases (9/11 *vs.* 5/11), abdomen recurrences (17/17 *vs.* 14/17) and lymphatic metastases (9/9 *vs.* 7/9). Tumour to background ratio was also increased in the cases mentioned above: 2.3–16.2 (mean 4.7) after i.p. *vs.* 1.2–3.5 (mean 2.1) after i.v. ($P<0.001$). Subtraction technique was not needed in detecting liver metastases, even if $^{131}$I label was used. This was due to the low non-specific uptake in the normal organ. We injected simultaneously, both i.v. and i.p., the same antibody labelled with two different isotopes in 4 patients bearing primary colon cancer. Tumours were removed and counted in a multichannel system. In these cases, the antibody concentration as well as the tumour/BK ratio were not increased by the i.p. route of injection if the neoplastic cells did not cross the peritoneal wall.

### Diagnostic contribution of I-131 $HMFG_2$ in malignant pleural and peritoneal effusions

J. Malamitsi, D. Skarlos, D. Pektasidis, G. Aravantinos, P. Papakostas, A. Varvarigou, J. Taylor-Papadimitriou, A.A. Epenetos & K. Koutoulidis

*Nuclear Medicine and 1st Internal Medical Departments, NIMTS, Athens, Greece and Imperial Cancer Research Fund, London WC2, UK.*

The aim of this study was to image pleural and peritoneal metastases of breast and ovarian carcinomas using $^{131}I$ $HMFG_2$ monoclonal antibody. Fourteen patients were examined, 7 with pleural metastases from breast carcinoma and 7 with peritoneal and pelvic metastases from ovarian carcinoma. After confirming the positive reaction of $HMFG_2$ with the breast and ovarian cancer cells of the patients under study by the indirect immunoperoxidase staining method, radiolabelling of $HMFG_2$ with $^{131}I$ followed (iodogen or N-br-succinimide method). After drainage of the serous effusion, 500 $\mu$Ci of $^{131}I$ $HMFG_2$ was administered intrapleurally or intraperitoneally respectively. Following that, the patients were scanned at 0, 2, 24, 48, 72 and 96 h. Radioactivity was counted in blood and urine specimens over 96 h and total excreted radioactivity in urine was calculated.

The selective localisation of radiolabelled $HMFG_2$ on pleural and peritoneal metastases was proven over 96 h, whilst radioactive concentrations in urine and blood as well as total excreted radioactivity in urine were negligible.

We conclude that by intrapleural and intraperitoneal administration of radiolabelled $HMFG_2$ in patients with breast and ovarian cancer respectively, a very satisfactory selective imaging of metastases, not easily detectable by other means, is achieved.

### Abstracts of poster exhibits

### Serological monitoring of breast cancer by a low pH ELISA method

B. Dhokia[1,2], N. Courtenay-Luck[1,2], D. Pectasides[1], J. Taylor-Papadimitriou[2], C. Self[1], N.A. Habib[1], M. Hershman[1], C.B. Wood[1], A.J. Munro[1] & A.A. Epenetos[1]

[1]*Royal Postgraduate Medical School, London W12 and* [2]*Imperial Cancer Research Fund, London WC2, UK.*

A new, simple and sensitive low pH ELISA method has been developed to measure serum levels of tumour associated antigens detectable by monoclonal antibodies HMFG1 and HMFG2. We examined sera from healthy controls and patients with noeplastic and non-neoplastic conditions of breast. The majority of patients with metastatic breast cancer had elevated serum antigens (69% HMFG1, 72% HMFG2) compared to healthy controls (6.3% HMFG1, 3.0% HMFG2) or patients with benign breast disease (17% HMFG1, 4% HMFG2).

This new method promises to be of value in the assessment of patients with breast cancer.

### Use of monoclonal antibodies to detect circulating epithelial membrane antigens in breast cancer patients

J.A. Kantor, W.F. Feller, J. Hilkens & J. Hilgers

*Department of Surgery, Georgetown University, Washington, DC, USA and Netherlands Cancer Institute, Amsterdam, The Netherlands.*

We have used two murine monoclonal antibodies, 115D8 and 67D11, derived from antigens present on human milk fat globule membranes to detect circulating antigens in human plasma. Plasma samples from 61 women with breast cancer and 164 healthy controls were studied with these two murine monoclonal antibodies and a low pH enzyme linked immunoabsorbent assay (ELISA). 92% (56/61) of all women with breast cancer had elevated levels of monoclonal antibody defined antigen in their plasma as compared to 6% (10/164) of healthy controls. All women (6/6) with benign proliferative breast disease had elevated levels of circulating antibody defined antigens. We conclude that monoclonal antibodies reactive to epithelial cell membrane antigens can detect elevated levels of these antigens in patients with breast cancer and benign proliferative breast disease.

### Detection of the MAM-6 antigen in sera and urine from breast cancer patients

D.E.A. Zanin, P.F. Bruning & J. Hilkens

*Department of Tumour Biology, The Netherlands Cancer Institute, Plesmanlaan 121, 1066 CX Amsterdam, The Netherlands.*

MAM-6 is a glandular epithelium associated antigen reactive with monoclonal antibody 115D8. The antigen is highly glycosylated and has an apparent molecular weight over 400 kD. MAM-6 is present on several carcinomas, but very high expression has been observed on breast carcinomas. Increased MAM-6 levels were found in serum of 80% of the patients with advanced breast and ovarian carcinoma.

TCA precipitable material immunoreactive with 115D8 was present at low levels in the urine of apparently healthy individuals ($n=18$) and increased levels were observed in urine of breast carcinoma patients ($n=35$).

We measured the urinary excretion of the renal tubular enzymes alanine-aminopeptidase (brush-border enzyme) and N-acetyl-B-glucosaminidase (lysozomal enzyme). These enzymes were not correlated with the excretion of MAM-6 like material, suggesting that the latter was not of renal origin. The creatinine clearance rate was normal in all the healthy volunteers and most of the patients, and not correlated with the clearance rate of MAM-6. Normal individuals had low serum levels and high MAM-6 clearance rates and in patients serum levels were inversely related to the MAM-6 clearance rate. Further studies to investigate the nature of the 115D8 reactive material and the mechanism of its excretion in urine of healthy and advanced breast cancer patients are in progress.

### The clinical significance of pre-operative CA125 in ovarian cancer

D.J. Cruickshank

*Department of Obstetrics and Gynaecology, University of Aberdeen, Scotland, UK.*

There is now an extensive literature confirming that CA125 is the most clinically useful marker in epithelial ovarian cancer with a reported sensitivity of 82% and 93% concordance with clinical disease status. The published literature to date, however, deals almost exclusively with post-operative sera from patients with perhaps poorly quantified persistent or recurrent disease. The aim of this study was to evaluate the significance of pre-operative serum levels.

Pre-operative serum CA125 estimations were performed in 41 patients who subsequently had epithelial ovarian cancer histologically confirmed. Both the frequency of positively ($CA125 \geq U ml^{-1}$) and absolute CA125 level correlated with FIGO stage (tumour load). All histological types released CA125 into the serum although serous and undifferentiated tumours secreted quantitatively more antigen. There was no correlation between CA125 level and histological grade suggesting CA125 is not a differentiation antigen. Similarly, pre-operative CA125 did not predict outcome as defined by clinical status (responder or non-responder) at last presentation.

In this series the sensitivity of CA125 was 73% (30/41). FIGO Iaii was the earliest stage disease expressing significant pre-operative levels of CA125 (2/5) with 0/6 FIGO Iai cases positive. This may be because the tumour capsule must be breached (FIGO Iaii) to release the antigen or the threshold of detection of CA125 is associated with a tumour volume greater than that found in Iai disease.

### The role of c-*myc* oncogene expression in the development of gastric cancer

F. Macdonald, W.H. Allum, H. Stokes, B. Russell & J.W.L. Fielding

*Surgical Immunology Unit, Queen Elizabeth Hospital, Birmingham, UK.*

Oncogenes have been associated with a variety of different cancers including those of the gastro-intestinal tract. We have used a monoclonal antibody to the p62 protein product of the *myc* oncogene in an immunoperoxidase study of normal stomach, premalignant and inflammatory lesions of the stomach as well as gastric cancer and autologous lymph node metastasis to determine whether this oncogene plays any role in the development of gastric malignancies.

Staining occurred in either the parietal cells and/or the surface epithelium depending on the pathology. Parietal cells were positive in all 16 normal stomachs tested. Three showed weak staining of less than 5% of the surface epithelium. Biopsies from chronic superficial gastritis were weaker in comparison, with only 7/19 positive, again in the parietal cells. Only one biopsy showed intense surface staining. 9/20 biopsies from intestinal metaplasia were positive in the parietal cells and 9 also showed surface staining. Dysplastic lesions exhibited the most intense staining. Parietal cells in all 20 biopsies were strongly positive. Over 50% of surface cells were positive in 8/20 specimens, between 25 and 50% of cells were positive in a further 4. The remaining 8 were either negative or weakly reactive and these were classified primarily as mild dysplasias. A wide range of reactivity was seen in the cancers and their lymph node metastases although the intensity of staining was never as strong as in the dysplastic lesions.

These results suggest that increased expression of the *myc* oncogene is an early event in the development of gastric cancer.

**Reactivity of monoclonal antibodies with human gliomas**

D. Stavrou, K. Staemmler, E. Keiditsch, F. Schmidberger, I. Funke & P. Mehraein

*Institute of Pathology and Department of Neurosurgery, Clinicum Bogenhausen; Institute of Immunology and Institute of Neuropathology, University of Munich, FRG.*

BALB/c mice were immunised with cells from a malignant astrocytoma. Hybridoma cells were tested for antibody production against different cell monolayers and tissue sections. The binding of Mabs MUC-1 to MUC-15 is shown in the table:

| | *Murine monoclonal antibodies* | | |
|---|---|---|---|
| *Human tissue* | *MUC-1* | *MUC-2* | *MUC-3* |
| Normal brain (1.occip., m., 70 yrs) | + | + | + |
| Embryonic cells (brain anlage, 9 wks) | + | + | + |
| Blood monocytes, colon carcinoma, breast carcinoma | – | – | – |
| Melanoma, astrocytoma, glioblastoma | + | + | + |

| | *Murine monoclonal antibodies* | | |
|---|---|---|---|
| *Human tissue* | *MUC-4* | *MUC-5* | *MUC-7 to MUC-15* |
| Normal brain (1.occip., m., 70 yrs) | + | + | – |
| Embryonic cells (brain anlage, 9 wks) | + | + | + |
| Blood monocytes, colon carcinoma, breast carcinoma | – | – | – |
| Melanoma, astrocytoma, glioblastoma | + | + | + |

This shows that the established hybridomas produce antibodies which bind to different cell types enabling discrimination between normal and neoplastic brain cells. Variable antigenic profiles among glioma cell populations could be demonstrated. Antigens recognised by the MUC-7 to MUC-15 antibodies persist after fixation with acetone, glutaraldehyde and Bouin solutions and are likely to be differentiation antigen(s).

**Immunophenotype of cytomorphologically defined acute lymphoid leukaemias**

D. Batinić & M. Boranić

*Ruder Bošković Institute, Zagreb, Yugoslavia.*

The diagnostic value of a selected panel of monoclonal antibodies specific for lymphoid cells was assessed in 39 patients (23 children and 16 adults) with newly diagnosed acute leukaemia, initially assigned as acute lymphoid leukaemia (ALL) by cytomorphological criteria. There were an additional 6 children with ALL in relapse. Samples (peripheral blood and bone marrow) containing more than 30% blasts were accepted for the study. Using Coulter Clone monoclonal antibodies T11, T4, T8, B1, I2 (anti-Ia) and J5 (CALLA) in indirect immunofluorescent test and FITC-conjugated rabbit anti-human Ig antiserum (Inep-Torlak) in direct immunofluorescent test, 4 major blast phenotypes could be distinguished: T (T11, $T8^{+/-}$, $T4^{+/-}$), B (Ia, B1, mIg), pre-B 'common' (Ia, CALLA, $B1^{+}$ or $B1^{-}$), and 'null' ($Ia^{+}$ or $Ia^{-}$). In view of the relatively high proportion of negatively-defined 'null' type leukaemias (11 of 45, especially in children – 5/23 newly diagnosed and 2/6 in relapse), some of the antibodies used for the first-line screening of acute leukaemias should be replaced by those determining more precisely the cellular origin of 'null' leukaemias, e.g. by pan-antibodies (for T-, B- and myeloid cells) that have become commercially available.

### Site and accessibility of $AUA_1$ antigen: Its relevance to antibody-guided irradiation

W.J. Mooi, T. Krausz, S. Kirkland, A. Cross & A.A. Epenetos

*Departments of Histopathology and Clinical Oncology, Royal Postgraduate Medical School, London W12, UK.*

The success of antibody-guided irradiation of tumours is not as constant and complete as one might anticipate on the basis of immunostaining results of tumour material. An explanation may be that antigens are present on tumour cells but may be inaccessible due to their location in the *in vivo* situation.

To investigate this, we assessed the reactivity of a colon carcinoma cell line, HRA-19, before and after alcohol fixation, to the monoclonal antibody $AUA_1$, recognising an antigen present on the basolateral surface of neoplastic and non-neoplastic epithelial cells. The HRA-19 cells were grown on glass coverslips, forming compact colonies. After incubation of live cells with $AUA_1$, the cells were alcohol fixed for immunoperoxidase staining. The results were compared to the immunoreactivity when the cells were alcohol-fixed prior to incubation with $AUA_1$.

The live incubation resulted in staining of a rim of cells at the periphery but not in the centres of the colony. Alcohol fixation prior to incubation with $AUA_1$ resulted in strong staining of cell membranes also in the colony centres. This latter pattern was also seen when $AUA_1$ was applied twice: on live cells and again after alcohol fixation. This excluded the possibility of antibody-induced antigenic modulation. Incubation of live cells with EDTA (which induces dissociation of the live cells) also resulted in strong staining of colony centres.

We conclude that accessibility of antigen is an important factor in antibody binding to live cells. In view of these findings, we suggest that screening of immunoreactivity of cancer cells for antibody-guided irradiation should be carried out on live tumour material.

### CA-50 radioimmunoassay inhibition test for diagnosis of prostatic carcinomas

M.J. Hershman, N.A. Habib, M.A. Ferro, C.J. Roberts, K.M. Campion, F. Haberland, R.C.N. Williamson & C.B. Wood

*Departments of Surgery, Royal Postgraduate Medical School, London and Bristol Royal Infirmary, Bristol, UK.*

Cancer cells may secrete or express on their cell surfaces 'foetal components' not normally present in adult cells and may be detected in serum as 'oncofoetal antigens'. Recently, a new antigen, CA-50, has been isolated as a monosialoganglioside which is shed into the serum and can be detected using a radio-immunoassay technique. CA-50 has been shown to be raised in between 50–70% of patients with colorectal, pancreatic and liver cancer. This study assessed the significance of CA-50 in benign and malignant lung disease. Based on a normal population study a level of $17\,U\,ml^{-1}$ was used as a cut-off level between benign and malignant diseases.

The serum CA-50 level was measured in 60 normal individuals and 61 with benign prostatic hypertrophy. All normal subjects and 59 of 61 (97%) patients with BPH had levels below $17\,U\,ml^{-1}$ with mean values of $8.7 \pm 5.3$ and $5.0 \pm 4.3$ respectively. Twenty-nine of 69 (43%) patients with prostatic cancer had raised serum levels (mean of positive values, $32.4 \pm 16.2$). Thirty-four patients had metastases of which 24 had positive values (71%), significantly different from 5 positive values in 34 patients without metastases (15%) ($P < 0.001$). Zero of 8 well differentiated tumours were positive compared with 11 of 33 moderate differential tumours (33%) and 18 of 27 poorly differentiated tumours (66%).

These data suggest that CA-50 levels correlate with stage and grade of prostatic cancer for which it has a high specificity and may therefore have a role as a tumour marker in this condition.

### The expression of *sis* gene in the colonic adenoma-carcinoma sequence

N.A. Habib, G.U.A. Igboaka, M.J. Hershman, R.C.N. Williamson, C.B. Wood & J.W.B. Bradfield

*Departments of Pathology and Surgery, Bristol Royal Infirmary, Bristol and Department of Surgery, Royal Postgraduate Medical School, London, UK.*

The structural and immunological relation between the *sis* gene product (p20) and platelet-derived growth factor (PDGF) provide a link between the transforming properties of oncogenes and the mitogenic action of growth factors.

The purpose of this study was to investigate the expression and distribution of the human c-*sis* oncogene product, p20, by peroxidase-anti-peroxidase technique using monoclonal antibodies to p20, in normal and neoplastic colonic epithelium. We have examined 20 normals, 85 tubulovillous adenomas (24 with mild dysplasia, 14 moderate dysplasia, 31 severe dysplasia and 16 with invasive cancer). It was found that there was no difference in staining intensity or distribution in the superficial parts of the mucosa in both normal and neoplastic tissue. By contrast, p20 was absent in the deep parts of the glandular epithelium in normal mucosa and it was present with varying degrees in neoplastic tissue. There was a significant increase in the p20 staining of the deep parts of the mucosa as the tissue progressed from mild (12%) through severe dysplasia (23%), reaching 88% incidence in invasive cancer.

### CA-50 radioimmunoassay inhibition test in liver, pancreatic and colonic carcinomas

M.J. Hershman, N.A. Habib, C.J. Roberts, K.M. Campion, R.I. Swift, V. Kallmeyer, R.C.N. Williamson & C.B. Wood

*Departments of Surgery, Royal Postgraduate Medical School, London and Bristol Royal Infirmary, Bristol, UK.*

Recently, a new antigen, CA-50, has been isolated as a monosialoganglioside. This antigen is shed into the serum and can be detected using radioimmunoassay technique. The antigen level of $17\,U\,ml^{-1}$ based on normal population study was used as a cut-off level between benign and malignant diseases.

The clinical application of this test in colorectal diseases has shown that all normal subjects ($n=50$) and control patients with inflammatory diseases ($n=20$) had levels below $17\,U\,ml^{-1}$. This test was positive in 40 of 77 (51%) patients with colorectal carcinomas. In the field of pancreatic diseases, 8 of 9 patients with pancreatitis were negative. Twenty-four of 26 (92%) patients with pancreatic carcinoma had CA-50 levels above $17\,U\,ml^{-1}$. Lastly, in liver diseases, we found that 25 of 28 (89%) patients with benign liver diseases had a CA-50 level below $17\,U\,ml^{-1}$, while 2 patients with sclerosing cholangitis and 55 of 88 (63%) of patients with liver tumours were positive.

The CA-50 test seems to be a useful non-specific tumour marker.

### CA-50 radioimmunoassay inhibition test for diagnosis of lung carcinomas

N.A. Habib, M.J. Hershman, C.J. Roberts, R. Stein, D.T. Reilly, R.I. Swift, R.C.N. Williamson & C.B. Wood

*Departments of Surgery, Bristol Royal Infirmary and Royal Postgraduate Medical School, London, UK.*

This study assessed the significance of CA-50 in benign and malignant lung disease. based on a normal population study, a level of $17\,U\,ml^{-1}$ was used as a cut-off level between benign and malignant diseases. The serum CA-50 level was measured in 50 normal individuals and 28 with inflammatory lung conditions, including 10 TB, 3 sarcoid, 6 alveolitis, 9 acute bronchitis + pneumonia. All normal subjects and 27 of 28 (96%) patients with benign lung conditions had levels below $17\,U\,ml^{-1}$ with a mean value of $10.1 \pm 4.9$. Twenty-one of 55 (38%) patients with lung cancer had raised serum levels (mean $32.3 \pm 16.5$). Six of 13 (46%) squamous tumours were positive, compared to 14 of 24 (58%) oat cell tumours, 1 out of 4 adenòcarcinomas, none of 12 large cell tumours, and none of 2 lymphomas.

These data suggest CA-50 may be a useful tumour marker for lung cancer, particularly if used in conjunction with other markers.

### The localisation of the human c-*myc* gene product (p62) in normal and neoplastic human colonic mucosa

G.U.A. Igboaka, N.A. Habib, M.J. Hershman, R.C.N. Williamson, C.B. Wood, J.W.B. Bradfield, G. Evan & K. Sikora

*Departments of Pathology and Surgery, Bristol Royal Infirmary, Bristol and Departments of Clinical Oncology and Surgery, Royal Postgraduate Medical School, London, UK.*

Cellular oncogenes are concerned with the regulation of normal cell growth and differentiation. Over-expression of these genes may be associated with some malignant diseases. The purpose of this study was to examine the expression and distribution of the human c-*myc* gene product, p62, by peroxidase-antiperoxidase technique using monoclonal antibodies to p62, *myc*-1 6E10 in normal and neoplastic colonic epithelium.

We examined 20 normal, 75 tubulovillous adenomas (18 with mild dysplasia, 16 moderate dysplasia, 31 severe dysplasia and 10 with invasive cancer).

The distribution and the intensity of staining with *myc*-1 6E10 showed a pattern. The intracytoplasmic p62 increased significantly as the tissue progressed from mild through severe dysplasia to invasive cancer. With increasing dysplasia, there was an increase in the oncogene expression at the lower parts of glandular epithelium.

### The expression of *ras*-oncogene in the normal and neoplastic colonic mucosa

G.U.A. Igboaka, N.A. Habib, J.W.B. Bradfield, M.J. Hershman, R.C.N. Williamson & C.B. Wood

*Department of Surgery, Royal Postgraduate Medical School, London and Departments of Pathology and Surgery, Bristol Royal Infirmary, Bristol, UK.*

Oncogene over-expression is associated with malignant transformation of normal cells. Activation of c-*ras* oncogene, which is situated on chromosome 12 in humans, has been demonstrated in colonic carcinomas. The purpose of this study was to examine the expression and distribution of the normal form of *ras*-oncogene product (p21) by peroxidase-antiperoxidase technique using monoclonal antibodies to p21 in normal and neoplastic colonic epithelium.

We have examined 20 normal, 21 metaplastic polyps, 75 tubulovillous adenomas (6 with mild dysplasia, 27 moderate dysplasia, 32 severe dysplasia and 10 with invasive cancer). It was found that the p21 expression significantly increased as the tissue progressed from normal through mild and severe dysplasia and finally invasive cancer. In metaplastic polyps there was a decrease in p21 expression compared to normal. By contrast, in neoplastic tissue, both the intensity and distribution of p21 showed a pattern in that with increasing dysplasia there was an increase in expression of p21 in the lower parts of the glandular epithelium.

### Immunohistochemical localisation of tumour markers in a human cell line

T.W. Briers, E.J. Nouwen, S. Dauwe, P. Stroobants, P.G. Hendrix, D.E. Pollet & M.E. De Broe

*Department of Nephrology-Hypertension, University Hospital, Antwerp, Belgium.*

The cell line DOooo-s originated from the ascites of a patient with a mucinous cystadenocarcinoma of the ovary (January, 1985). The cells were cultured as a suspension in RPMI-1640 supplemented with 2% FCS or in Dulbecco's MEM supplemented with 5% FCS. They were vermiform and appeared as clusters of 10 to 30 cells surrounded by a glycoprotein layer.

Prominent nuclei, numerous vacuoles, lipid droplets, and bizarre mitochondria were ultrastructural characteristics of these cells. HPLAP, CA125, HMFG (1 & 2), CA19-9 and CEA were localised on paraffin sections using monoclonal antibodies (respectively H17E2, 17E3, E6 and H327; OC125; HMFG1 and 2; the Hybritech antibody). All cells were positive for CA125 and HMFG2. The stain was normally found on the plasma membrane and some staining was present in the cytoplasm. For HMFG1, small groups of cells were positive. Only 17E3 and E6 could visualise HPLAP in single or small groups of cells. Histochemical AP activity was completely inhibited by L-phenylalanine and was almost not influenced by L-bromotetramisole. These results indicate that in DOooo-s AP is present as HPLAP. The cells were not positive for CA19-9. Light microscopic analysis was confirmed by electron microscopy of immunohistochemically stained cells. In xenografts induced in athymic nude mice the same immunohistochemical pattern could be demonstrated.

### Construction and characterisation of a murine/human chimeric MAb using recombinant DNA techniques

G.P. Moore, S. Sahagan, H.D. Dorai, J. Saltzgaber-Muller, G. Davis & F. Tonneguzzo

*E.I. du Pont de Nemours, Co., Inc., No. Billerica, MA, USA.*

Chimeric immunoglobulin genes were constructed by fusing murine variable region exons to human constant region exons. The ultimate goal was to produce an antibody capable of escaping surveillance by the human immune system while retaining the tumour specificity of a murine monoclonal. The murine variable regions were isolated from the functionally expressed kappa and gamma 1 immunoglobulin genes of the murine hybridoma cell line B6.2, whose secreted monoclonal antibody reacts with a surface antigen from human breast, lung and colon carcinomas. The kappa and gamma 1 chain fusion genes were co-introduced into non-antibody producing murine myeloma cells by electroporation. Transfectants which produce murine/human chimeric antibody were obtained at high frequency as indicated by immunoblots probed with an antisera specific for human immunoglobulin. ELISA analysis demonstrated that this chimeric antibody is secreted from the myeloma cells and retains the ability to bind selectively to human tumour cells. The ability of chimeric antibody to recognise human tumour-associated antigen makes feasible a novel approach to cancer immunotherapy.

**Yttrium-90 radiolabelling of the monoclonal antibody H17E2. Preliminary results**

D. Snook & A.A. Epenetos

*Royal Postgraduate Medical School, London W12 and Imperial Cancer Research Fund, London WC2, UK.*

Placental alkaline phosphatase (PLAP), the antigen for H17E2, is expressed in a variety of neoplasms. Indium-111 ($^{111}In$) labelled H17E2 has been used successfully for radioimmunodetection in patients. Yttrium-90 ($^{90}Y$) is a potentially useful isotope for radioimmunotherapy because it is a pure $\beta$-emitter ($E\beta$max 2.3 MeV, $t_{\frac{1}{2}}$ 64 h), can be generator produced and bound to antibodies by the same method as $^{111}In$ (Hnatowich *et al.*, *J. Nucl. Med.*, **26**, 503, 1985).

H17E2 was conjugated to diethylenetriaminepentacetic acid (DTPA) using an anhydride to antibody molar ratio of 42:1. An average of 1.6 DTPA molecules per H17E2 molecule were bound.

$^{90}Y$ was produced at AERE Harwell and contained $<1\,\mu$Ci Strontium-90 mCi$^{-1}$ $^{90}Y$. $^{90}Y$ in 0.04 M HC1 was mixed with 3.8% (w/v) sodium citrate, the H17E2-DTPA conjugate added and incubated for 2 h at 22°C, pH 7.0. Purification by Sephadex G50 column gave a labelling efficiency of 61%. A specific activity of $\simeq 0.5\,\mu$Ci $^{90}Y\,\mu g^{-1}$ H17E2 was achieved.

All subsequent studies occurred 24 h post-preparation. A live cell radioimmunoassay of H17E2-DTPA-$^{90}Y$ on HEP2 cells (PLAP +ve) and MCF7 cells (PLAP −ve) showed 51% and 4% binding respectively. HPLC analysis revealed 97% of the $^{90}Y$ on monomeric IgG, the remaining 3% being free isotope.

These preliminary results are promising since 24 h post-preparation >50% antigen binding is retained with no evidence of radiolysis or aggregation.

**Extended analysis of site-specific antibody labelling methodology: 111-Indium radioimmunoimaging in nude mice with MAbs against renal cell, colon and breast carcinoma**

V.L. Alvarez, A.D. Lopes, C. Lee, J.D. Rodwell & T.J. McKearn

*Cytogen Corporation, Princeton, NJ 08540, USA.*

Site-specifically modified monoclonal antibodies have been shown to give excellent 111-Indium radioimmunoimaging in a BN-rat lymphoma xenograft system (Rodwell *et al.*, *Proc. Natl Acad. Sci.*, in press). We report here several other systems using human tumour xenografts in nude mice and monoclonal antibodies characterised by other groups. These are: a renal cell carcinoma cell line, 7860 and two antibodies, A6H (IgG1; Moon *et al.*, *J. Urol.*, **130**, 584, 1983) and S4 (IgG2a; Cordon-Cardo *et al.*, *J. Hist. Cyt.*, **32**, 1035, 1984), a colon carcinoma using the cell lines HT29 and SW403 and the antibody HT29.15 (IgG1; Ueda *et al.*, *Proc. Natl Acad. Sci.*, **78**, 5122, 1981); and the breast/colon tumour-reactive antibodies B72.3 and B6.2 (both IgG1; Schlom *et al.*, *Adv. Cancer Res.*, **43**, 143, 1985) tested on xenografts of the colon cell line LS174T and breast cell lines MS-1, BT20 and ZR75-1. Each antibody/tumour system gave excellent imaging results, substantiating the benefit of site-specific modification. Immunoreactivity was largely retained, with values ranging from 98% of the control for A6H and S4 to better than 90% for B72.3. Among the differences noted were: one, rate of localisation. Both anti-renal cell carcinoma antibodies localised within 6 h, while the B72.3 showed optimal localisation only after 72 h. Other antibodies showed intermediate localisation times. Two, target antigen variability. HT29.15 recognised a shed antigen on the xenograft HT29.15, but the antigen on SW403 appeared not to be shed. Finally, the dose of antibody that saturates each tumour ranged from 10 $\mu$g antibody g$^{-1}$ tumour for the S4/7860 system to greater than 200 $\mu$g g$^{-1}$

tumour for the B72.3/LS174T system. We conclude that site-specific modification of tumour-reactive monoclonal antibodies results in excellent imaging and biodistribution results.

### Labelling of monoclonal antibodies with a $^{67}$Ga phenolic aminocarboxylic acid chelate (P-EDDHA)

J. Schuhmacher & S. Matzku

*Institute of Nuclear Medicine, German Cancer Research Center, Heidelberg, FRG.*

Labelling of antibody with $^{67}$Ga was achieved by coupling of the pre-formed Ga complex of propionic acid substituted ethylenediamine-N,N′-di-⟨(o-hydroxyphenyl)acetic acid⟩ via carbodiimide to the anti-melanoma monoclonal antibody M.2.9.4. This was accompanied by a low degree of oligomerization but a considerable degree of intra-molecular cross-linking, which however, did not impair immunoreactivity nor the half-life of labelled antibody *in vivo*. Biodistribution analysis in normal mice in comparison to the $^{131}$I-labelled antibody showed Ga:I ratios near unity in the blood and in all tissues devoid of degradative or excretory potential. In tissues of the reticulo-endothelial system and in the kidneys, Ga:I ratios up to 2.5 were reached within 4 days after application. In the antigen-positive MeWo tumour, $^{67}$Ga retention was clearly superior, so that the tumour:organ ratios obtained with the $^{67}$Ga-labelled antibody were higher than those of the $^{131}$I-antibody in all organs but the liver. It is concluded that the method of coupling pre-established $^{67}$Ga P-EDDHA chelate to antibody results in a functionally intact tracer molecule, whose persistence *in vivo* is not significantly impaired. The major difference to I-labelled antibodies may consist in a prolonged retention of Ga in tissues (cells) involved in antibody catabolism.

### Factors affecting the routine preparation of In-111 DTPA labelled antibodies

A.P. Richardson[1], P.J. Mountford[1], E. Heyderman[2] & A.J. Coakley[1]

[1]*Department of Nuclear Medicine, Kent and Canterbury Hospital, Kent CT1 3NG, and* [2]*United Medical and Dental Schools, St. Thomas's Hospital, London SE1, UK.*

Radioimmunolocalisation using antibodies labelled with In-111 DTPA has been the subject of research at a number of centres and consequently a range of labelling regimes is practised. Wider use of the technique requires the availability of more effective antibodies and the improvement and standardisation of labelling methods.

We have examined the labelling of antibodies with In-111 and report a simple procedure which can be completed to pharmaceutical standard in less than one working day. Labelling efficiency, antibody activity and reaction rate (see table below) are examined.

*Rate of chelation of In-111 DTPA-antibody (by TLC)*

| *Time (h)* | *Labelling efficiency (%)* |
|---|---|
| 0 | 5.6 |
| 0.08 | 42.3 |
| 0.25 | 70.9 |
| 0.75 | 85.0 |
| 2.25 | 89.0 |
| 6.75 | 87.9 |
| 23.30 | 88.2 |

Furthermore, we show that antibodies may be conjugated with DTPA in batch (with quality control carried out at this stage). Samples were sterilized and stored until required. In-111 chloride was then simply added and the efficiency of labelling determined by TLC. By this means a regional centre with access to antibodies could act as a source of pharmaceutical grade DTPA-antibody in 'kit' form. It would distribute these antibody kits to other departments to be stored until required when simple labelling with In-111 is completed in one hour to be ready for use in patient studies.

### $^{199}$Au, $^{111}$Ag, $^{143}$Pr-radionuclides for radioimmunotherapy in India

D.K. Hazra[1], S. Dass[2], V. Lahiri[1], M. Kumari[1], S. Saran[1] & R. Singh[1]

[1]*Department of Nuclear Medicine, SN Medical College, Agra and* [2]*Department of Chemistry, Dayalbagh Educational Institue, Dayalbagh, Agra, India.*

Radioimmunotherapy (RIT) requires the use of isotopes emitting high LET radiations, alpha, energetic beta or auger electrons. Their half-life should permit not only radiochemical processing but also tumour accumulation and adequate tumour residence time; and preferably minimal bone localisation in the event of the isotope being deconjugated from the antibody. The daughter products should not be undesirable.

Cyclotron-produced radionuclides are not available in India. Out of a short list of about 15 radionuclides, $^{199}$Ag, $^{111}$Ag, $^{143}$Pr, $^{122}$Sb and $^{186}$Re are suitable from the above viewpoints. The radiochemistry and preparation of these radionuclides in carrier-free state is described *vis à vis* their suitability for linkage to bifunctional chelates and relevant methods are being optimised and will be described.

### Radiolabelled monoclonal antibodies against human carcinomas: Effect of incubation in human serum on the binding activity

M. Ripamonti, S. Canevari, S. Camagni, D. Mezzanzanica, R. Orlandi & M.I. Colnaghi

*Istituto Nazionale Tumori, Milan, Italy*

Murine monoclonal antibodies (MoAbs) with restricted reactivity for breast or ovarian carcinomas were labelled with $^{125}$I in the perspective of obtaining specific and stable radioimmunopharmaceutical reagents. Three radiolabelled MoAbs (MBrl and MOv2 of IgM class and MOv8 of IgG class) were found to maintain their integrity, homogeneity and binding specificity after iodination and were further analysed for their *in vitro* stability in human blood. The MoAbs were incubated at 37°C for various lengths of time in human or, as control, murine blood and their binding capacity, evaluated by solid-phase RIA, was compared with that obtained after incubation with buffer. In human blood, serum and plasma, but not with other components such as erythrocytes, leucocytes, HSA and IgG, the MoAbs revealed a loss of binding reactivity which was marked and constant for IgM MoAbs and only occasional for the IgG MoAb. In murine serum the decrease was not so evident nor so rapid. The same change in the binding capacity was observed when the MoAbs were labelled with $^{3}$H or $^{35}$S or when they were incubated in the presence of protease inhibitors. In the perspective of using IgM MoAbs for intraperitoneal therapy we are now evaluating the effect of ascitic fluid on their binding activity.

(Partially supported by grants from the Italian National Research Council, Contracts no. 85.02067.44 and 85.01499.57).

### *In vitro* removal of carcinoma cells from bone marrow using a pool of monoclonal antibodies and magnetic beads

G. Porro, S. Villa, S. Menard & M.I. Colnaghi

*Istituto Nazionale Tumori, Milan, Italy.*

Several methods involving monoclonal antibodies have been used to remove tumour cells from bone marrow in the perspective of autologous transplantation. However, due to tumour heterogeneity in particular, with these methods purging was incomplete. In order to overcome this obstacle, we selected different murine monoclonal antibodies which had complementary antitumour reactivities with the aim of creating a suitable pool. This pool was capable of recognising 100% of the tested carcinomas and 90–100% of the tumour cells within a given tumour and, moreover, it is unreactive with normal bone marrow cells. The usefulness of the pool for specific detection of metastatic cells in bone marrow suspensions was demonstrated. Subsequently, the pool was studied to evaluate its ability to purge bone marrow *in vitro*. To this end tumour cells, stained with bisbenzimide and treated with the pool, were artificially mixed with normal bone marrow cells. After incubation with antimurine Ig-linked magnetic beads, the cell suspension was passed through a magnetic field and the number of tumour cells still present was then determined by the count of bisbenzimide stained cells. This procedure allowed the removal of 75–95% of tumour cells, depending on the technique used. The possible negative effect on the the bone marrow stem cells was measured in CFU/c assays.

(Partially supported by a grant from the Italian National Research Council, Special Project 'Oncology', Contract no. 85.02103.44.)

### *In vivo* fate and anti-tumour activity of ricin A-chain and deglycosylated-ricin A-chain immunotoxins

D.C. Blakey, G.J. Watson, P.P. Knowles & P.E. Thorpe

*Imperial Cancer Research Fund, PO Box 123, Lincoln's Inn Fields, London WC2, UK.*

The ability of ricin A-chain immunotoxins to prolong the survival time of tumour bearing mice has been less dramatic than might have been expected from *in vitro* studies. This may reflect their rapid clearance from the blood possibly by hepatic reticuloendothelial cells which possess receptors that can bind the mannose terminal oligosaccharides present on ricin A-chain. The effect of chemical deglycosylation of ricin A-chain on the *in vivo* fate and anti-tumour activity of monoclonal anti-Thy1.1 antibody-ricin A-chain immunotoxins has been investigated. The ricin A-chain immunotoxin was rapidly removed from the bloodstream of mice, only 10% of the injected immunotoxin remaining in the bloodstream 3 h after i.v. administration. The major site of clearance was the liver, ~30% of the injected dose localising in this tissue within 10 min. The deglycosylated ricin A-chain immunotoxin was cleared less rapidly, 10% of the injected dose still remaining in the bloodstream after 16 h and liver uptake was greatly diminished. Both immunotoxins were equally effective at inhibiting protein synthesis *in vitro*, in AKR-A cells which express Thy1.1 antigen. In contrast, the deglycosylated ricin A-chain immunotoxin was significantly more effective than the native ricin A-chain immunotoxin at prolonging the survival time of mice injected with AKR-A cells intraperitoneally. It is concluded that recognition of the terminal oligosaccharides present on ricin A-chain does reduce the anti-tumour activity of ricin A-chain immunotoxins *in vivo* and that this can be overcome by chemical deglycosylation of the ricin A-chain.

### Identification of immunoreactive monoclonal antibody fragments for improved immunoscintigraphy

S.J. Mather & H. Durbin

*Imperial Cancer Research Fund, PO Box 123, Lincoln's Inn Fields, London WC2, UK.*

Recent results obtained in animal models and confirmed in patient studies have indicated that antibody fragments possess advantages over the whole immunoglobulin for *in vivo* localisation studies.

Proteolytic digestion of monoclonal antibodies does not always, however, follow classical textbook dogma. Different antibody isotypes respond to digestion in different ways and even within a class of antibody such as the common IgG1, large differences can be observed.

This report describes a method whereby immunoreactive products of antibody digestion can be easily identified. This permits parameters such as the choice of enzyme and digestion conditions to be optimised with a minimum of effort.

Following a suggested digestion protocol, the products of digestion are separated by SDS-polyacrylamide gel electrophoresis. The gel may be stained to indicate the number of protein fragments and their molecular weights. The proteins from an unstained gel are now transferred to nitrocellulose paper by 'Western blotting' and the nitrocellulose is incubated with an antigen probe relevant to the antibodies under investigation in order that immune fragments may be identified.

In addition to simplifying the digestion of an antibody of particular interest and promise, this procedure permits a series of closely related antibodies to be screened for the ability to produce good yields of immunoreactive fragments.

### Human recombinant γ-interferon enhances *in vivo* expression of HLA-DR antigen

G. Rowlinson[1], F. Balkwill[2], D. Snook[2,3], G. Hooker[3] & A.A. Epenetos[2,3]

[1]*MRC Cyclotron Unit, Hammersmith Hospital,* [2]*Imperial Cancer Research Fund, Lincoln's Inn Fields, London WC2 and* [3]*Royal Postgraduate Medical School and Hammersmith Hospital, London W12, UK.*

Athymic (nude) mice bearing subcutaneous human breast tumours were treated systemically with recombinant human interferon-γ, rHuIFN-γ. These tumours were phenotypically negative for HLA-DR prior to therapy, but after 4 days of treatment 80% of the cells expressed this antigen *in vivo*, as assessed by immunoperoxidase staining.

A radioiodine-labelled murine monoclonal antibody TAL-1B5 against HLA-DR specifically localised to the tumours in rHuIFN-γ-treated mice but not in control mice. Tumour to normal tissue ratios greater than 10 were obtained in treated mice. An isotype identical murine monoclonal antibody that did not react with this tumour did not show any specific localisation in control or rHuIFN-γ-treated mice.

These results demonstrate that specific localisation to tumours of radiolabelled monoclonal antibodies to HLA-DR can be facilitated by systemic therapy with 1FN-γ.

**Antibody guided localisation of intraperitoneal tumours following i.p. or i.v. antibody administration**

G. Rowlinson[1], D. Snook[2,3], A. Busza[1] & A.A. Epenetos[2,3]

[1]*MRC Cyclotron Unit, Hammersmith Hospital,* [2]*Imperial Cancer Research Fund, Lincoln's Inn Fields, London WC2 and* [3]*Royal Postgraduate Medical School, Hammersmith Hospital, London W12, UK.*

Intraperitoneal delivery of monoclonal antibodies may prove beneficial in the diagnosis and therapy of malignancies involving the peritoneum.

I.p. tumours of the colon carcinoma cell line LoVo were established in athymic (nude) mice by i.p. inoculation of a single cell suspension. Two preparations of monoclonal antibody AUA1, radiolabelled with 125-Iodine or 131-Iodine, were injected i.p. and i.v. into the same animals. Localisation was assessed by dissection and counting the activity in tumour and normal organs.

Tumour/tissue ratios 1 h after i.p. injection were approximately 50 times higher than after i.v. administration. This i.p./i.v. advantage fell to ~4 by 8 h, and to just greater than 1 by 24 h. When an irrelevant antibody raised against a different tumour was injected intraperitoneally, there was slight tumour uptake within the first 4 h, but this fell rapidly to normal organ levels by 12 h. In contrast, the specific antibody remained specifically bound to the tumour for several days.

**Xenograft localisation of a monoclonal antibody reacting with colorectal cancer**

C.Y. Yiu[1], L.A. Baker[1], K. Roberts[2], J.H. Westwood[3], R.C. Coombes[2,3] & C.G. Clark[1]

[1]*Department of Surgery, University College, London,* [2]*Royal Marsden Hospital, Surrey and* [3]*Ludwig Institute for Cancer Research, London Branch, UK.*

A mouse monoclonal antibody 77.1 of the IgG2a subclass showed positive immunocytochemical staining of colorectal cancer sections (Yiu *et al*, *Anticancer Res.*, **5**, 608, 1985) and with a renal carcinoma xenograft XKI. A xenograft localisation study was undertaken to assess its potential for clinical radioimmunodetection.

48.2, a mouse monoclonal antibody, which was non-reactive with XKI, was used as control. Antibodies were labelled with 125-Iodine by the iodogen method. XKI was implanted subcutaneously into flanks of nude mice. Each mouse was injected i.p. with 1–2 MBq (500–1000 ng) of labelled antibody and sacrificed 24, 48 and 72 h later (4 mice/time point/monoclonal).

The tumour to blood ratios (T:B) for 77.1 at 24, 48 and 72 h were mean $\pm$s.e. $1.37\pm0.08$, $1.88\pm0.45$, $2.36\pm0.32$; for 48.2 $0.40\pm0.08$, $0.49\pm0.06$, $0.53\pm0.005$. The localisation indices, i.e. T:B 77.1/T:B 48.2 were 3.42, 3.83 and 4.34.

The results showed that specific localisation of 77.1 was achieved with the xenograft and suggested its suitability for radioimmunodetection of colorectal cancer.

**Comparison of three monoclonal antibodies for immunoscintigraphy of human colon carcinoma in nude mice**

J. Gretarsdottir[1], E. Forssell[1], L. Jacobsson[1], S. Mattsson[1], S.B. Holmberg[2], L. Hafström[2], L. Lindholm[3], B. Karlsson[4] & O. Nilsson[4]

*Departments of* [1]*Radiation Physics,* [2]*Surgery,* [3]*Medical Microbiology and* [4]*Neurochemistry, University of Gothenburg, Sweden.*

The monoclonal antibodies C241, C242 and C151, reacting with different epitopes of a carcinoma-associated glycoprotein, Canag, have been used for experimental immunoscintigraphy. C241 reacts with Si-Le$^{a}$. All MAbs are of the isotype IgG1.

The MAbs were iodinated with $^{131}I$, using the iodogen method. Human colon carcinoma was inoculated into nude mice. The animals were injected with 2.5–3.0 $\mu$g Mab i.v., corresponding to an activity of 0.5–0.8 MBq. Gamma camera images were registered in the period 1–144 h after injection. Blood samples were drawn at different times. Plasma components were separated by exclusion chromatography and the activity in the various fractions was measured. The activity concentration in tumour and organs was measured after the animals were sacrificed.

| | *Tumour/blood ratio at* | | |
|---|---|---|---|
| *MAb* | *1 d* | *3 d* | *6 d* |
| C241 | 0.3–0.9 (0.5) | 0.2–1.6 (0.5) | 0.2–0.7 (0.6) |
| C242 | 0.3–0.9 (0.5) | 0.5–3.2 (0.7) | 0.4–1.1 (0.4) |
| C151 | — | 0.5–0.7 (0.6) | 0.6–1.0 (0.7) |

| | *Tumour/muscle ratio at* | | |
|---|---|---|---|
| *MAb* | *1 d* | *3 d* | *6 d* |
| C241 | 0.3–5.7 (2.1) | 0.7–9.4 (3.5) | 1.6–4.2 (3.6) |
| C242 | 2.3–4.9 (3.6) | 4.2–9.5 (5.1) | 0.2–5.5 (2.5) |
| C151 | — | 5.1–8.6 (5.8) | 3.4–10.9 (6.9) |

### Antibody-dependent cellular cytotoxicity against gliomas: A tracer study using the avidin–biotin system

T. Bilzer & S. Aumann

*Institute of Veterinary Pathology, University of Munich, FRG.*

Monoclonal antibodies (Mab) of $IgG_{2a}$ isotype raised against membrane components of an experimental glioma have been used for directing antibody-dependent cellular cytotoxicity against experimental gliomas. Mouse peritoneal macrophages were coupled *in vitro* with biotinilated Mab and administered i.p. in athymic, macrophage-depleted mice bearing s.c. tumour grafts. At 24, 48, 72 and 96 h after injection tumour tissue was investigated by post-embedding staining adding avidin-biotin-alkaline phosphatase and avidin-biotin-ferritin, respectively.

After 24 h Mab-macrophages were detectable in intratumour blood vessels and first signs of perivascular infiltration occurred. From 48 h to 96 h widespread distribution of Mab-macrophages in the tumour tissue developed. Electron microscopically, Mab-macrophages could be found in close apposition to glioma cells contacting them over Mab-coated areas. Target cell death seemed to be a common event, mostly in the mode of apoptosis.

(Supported by the K.L. Weigand Foundation (54773).)

### Antibody-guided detection of primary medullary carcinoma by means of $^{111}$In labelled anti-CEA monoclonal antibody: A case report

G. Moscatelli, M. Agostini, S. Benini, G. Paganelli, P. Riva & V. Tison

*Istituto Oncologico Romagnolo, 47023 Cesena, Italy*

The MoAb employed in this study was an IgG1 directed against the CEA peptide chain.

Since we found 60% positive staining in a large number of fresh frozen tissue sections from breast carcinoma, we decided to study by external body scintigraphy a patient who had already undergone operation for breast carcinoma.

This 40 year old patient had high levels of circulating CEA without clinical or radiological evidence of disease. The antibody-guided scan using $^{111}$In-labelled $F(ab')_2$ fragments of the MoAb mentioned above, detected an unsuspected accumulation area of radioactivity located in the thyroid, corresponding to a node of the gland: a fine needle aspiration showed a medullary carcinoma.

No positive scan was obtained when we injected, as negative control, a non-specific antibody directed against melanoma. This finding seems promising in the use of immunoscintigraphy for the follow-up of patients with thyroid medullary cancer.

### The pre-operative detection of axillary lymph node metastasis in breast cancer patients using I-123-labelled monoclonal antibodies

R. Mandeville, N. Pateisky, K. Philipp, E. Kubista, F. Dumas & B. Grouix

*Immunology Research Centre, Institut Armand-Frappier, University of Quebec, LDR, Canada and 1st Department of Obstetrics and Gynaecology, University of Vienna, Austria.*

In an attempt to improve the accuracy of pre-operative detection of axillary lymph node metastases, we have used a radiolabelled monoclonal antibody designated 3C6F9 and directed against a glycoprotein of 37,000 mol. wt found on the surface of primary and metastatic breast tumours. The 3C6F9 immunoglobulin (IgG2a) was purified, radiolabelled with I-123 and used for gamma-scintigraphy in 9 patients with the clinical diagnosis of breast tumours. Each patient received 1 mCi (specific activity 2 mCi $mg^{-1}$ of antibody) as a s.c. injection into the finger webs between the 2nd and the 3rd fingers of both hands, i.e. the healthy side serving as a control for the affected side. Images of lymph node metastases were visible 18 to 24 h after injection of the antibody and without the aid of background substration manipulations. Seven of the 9 patients studied were positive by scanning and 6 showed positive lymph node involvement by histopathology (true positive, 86%). Two patients did not show any iodine uptake in the axilla and were subsequently found to be free of lymph node metastases (true negative, 100%). Only one patient with a positive scan had no lymph node metastases on serial sectioning (false positive, 14%). These results given an overall specificity of RIS of 88% and demonstrate that 3C6F9 localises preferentially in affected axillary lymph node compared to normal lymph nodes. These preliminary results support the hope that this non-invasive approach can be used to image metastatic tumour deposits in axillary lymph node and can therefore have important implications in the diagnosis, staging and monitoring of breast cancer patients.

**The detection of c-*myc* oncogene product in patients with solid tumours**

S. Chan, G. Evan & K. Sikora

*Ludwig Institute for Cancer Research, MRC Centre, Hills Road, Cambridge, UK.*

We have studied the utility of the c-*myc* oncogene product as a tumour marker using a set of monoclonal antibodies raised against synthetic peptides constructed from sequence data of the human c-*myc* oncoprotein. Antibodies Myc1-9E10 and Myc1-CT14, raised against the C-terminal 32 amino acids, have been shown to detect specifically the 62,000 dalton c-*myc* gene product in tumour cells. Iodine-131 labelled Myc1-CT14 was injected i.v. into 14 patients with primary lung cancer. There was selective uptake at the primary tumour site of 12 patients, suggesting a large quantity of the p62-*myc* in these areas. The protein is usually found within the nucleus and it is unlikely that the antibody can enter this site for binding. It is probable that the oncoprotein is present in the extracellular space surrounding the tumour cells as a result of cellular breakdown. Shed oncogene product or their metabolites may be detected in the serum.

Immunoblotting of sera and urine with antibody Myc1-9E10 consistently revealed a single 40,000 dalton band (p40). Quantitative analysis using dilution dot immunoblotting demonstrated a considerable increase in the titre of p40 in the sera of 51 patients with a wide range of advanced solid tumours when compared to 17 healthy controls and 50 patients with nonmalignant diseases. Serial measurement of the p40 titre in 12 patients with resected colorectal carcinoma shows a gradual return to normal with a half-life of 7 days. Our data suggest that p40 may be a useful new marker for monitoring tumour activity.

**Immunoscintigraphy of bone metastases from breast carcinoma**

K.R. Roberts, D. Clarke, M.C. Ward, J.H. Westwood, V.R. McCready & R.J. Ott

*Royal Marsden Hospital and Ludwig Institute for Cancer Research, Sutton, Surrey, UK.*

The mouse monoclonal antibody 2-94-2 (an $IgG_3$) was raised against a crude membrane extract from a metastatic bladder carcinoma. Immunocytochemical staining indicated features related to epithelial membrane antigen (EMA) antibodies, one of which (LICR-LON-M8) has been used successfully to image bone metastases in breast cancer patients (Rainsbury *et al., Lancet,* **ii,** 934, 1983). The usefulness of 2-94-2 for clinical immunoscintigraphy in this group of patients was evaluated.

The antibody was conjugated to DTPA, labelled with $^{111}$Indium and injected i.v. into 8 patients, 7 of whom had previously received chemotherapy. The immunoscintigraphic results were correlated with the known sites of metastatic disease, assessed using a combination of the clinical findings, histology, MDP bone scans, radiographs and CT scans. No false positive antibody scans resulted. 2-94-2 was effective in imaging skull (6/6 patients) and femoral metastases (8/8) but only imaged 1/6 patients with rib metastases. High radiolabel uptake by the liver and spleen tended to obscure the lower thoracic and upper lumbar regions.

The results indicate that 2-94-2 immunoscintigraphy is not adequate by itself for detecting bone metastases from breast carcinoma; a cocktail of 2-94-2 and LICR-LON-M8 may be more sensitive in detecting such lesions.

**Selective modification of NMR relaxation time in human colorectal carcinoma using gadolinium-DTPA-Mab 19-9 complex. Use of different Gd-DTPA concentrations/MAb molecule**

C. Curtet, J. Bohy, C. Tellier, J.C. Saccavini & J.F. Chatal

*Unite 211, INSERM, Lab. RMN, Nantes and ORIS Industrie, Saclay, France.*

The use of paramagnetic agents for contrast enhancement may extend the diagnostic potential of nuclear magnetic resonance imaging (MRI). Monoclonal antibody 19-9 (MAb 19-9) against human colon adenocarcinoma was conjugated with gadolinium DTPA (Gd-DTPA) and used as contrast agent in NMR in an effort to improve target selectivity. Our previous work had indicated that Gd-DTPA MAb 19-9 in solution decreased $T_1$ relaxation of water protons at 90 MHz and that this effect was greater than in Gd-DTPA solutions. $T_1$ relaxation time at 90 MHz measured in tumours removed from nude mice, 24 h after injection of Gd-DTPA-MAb 19-9 (Gd 0.08 mmol $kg^{-1}$, 15 DTPA/MAb) decreased significantly (15%) as compared with the control group.

An attempt was made in the present work to enhance gadolinium concentration by coupling 16, 25 and 50 DTPA/MAb. Immunoreactivity was tested against cell lines in culture (human colorectal carcinomas HRT18, SW948; and breast carcinoma MDA) with 2, 16, 25, 50 DTPA/MAb. Immunoreactivity was not affected to 25 DTPA/MAb and decreased at 50 DTPA/MAb. Pharmacokinetics were performed with $^{153}$Gd-DTPA-73-3 (25 DTPA/MAb); the specific activity was 0.15 $\mu$Ci $\mu g^{-1}$ and 8% of the injected dose $g^{-1}$ of tissue was found at the tumour site without variation between days 1 and 5. $T_1$ relaxation time was calculated at 20 MHz on tumour grafted on nude mice removed at day 5. After injection of Gd-DTPA-73-3 (25 DTPA/MAb), 3 MAb concentrations were tested (60 $\mu$M, 30 $\mu$M, 15 $\mu$M). There was respectively a decrease of 22%, 16% and 10% of the $T_1$ compared with the control.

**Induction of an 'anti-idiotypic network' as a result of monoclonal antibody therapy**

N.S. Courtnay-Luck[1,2], A.A. Epenetos[1] & M.A. Ritter[2]

*Departments of [1]Clinical Oncology and [2]Immunology, Royal Postgraduate Medical School, London, UK.*

Repeated administration of radiolabelled murine monoclonal antibodies for the diagnosis and therapy of some malignant neoplasms results in the development of an 'anti-idiotypic network'.

Initially, the recipient develops a human anti-mouse response to antigenic determinants on both the Fc and, later, $F(ab')_2$ components of the murine immunoglobulin of which a component is anti-idiotypic (anti-$Id_1$). With further administration an anti-$Id_2$ antibody is produced which possesses the 'internal image' of the tumour antigen and consequently recognises the same antigen as the injected murine monoclonal. These antibodies can therefore be described as anti-tumour.

**Formation of immune complexes and the subsequent increase in clearance rate of radiolabel**

G. Hooker[1] & N. Courtnay-Luck[2]

*[1]Department of Medical Physics, Royal Postgraduate Medical School, London and [2]Department of Immunology, Royal Postgraduate Medical School of London and Imperial Cancer Research Fund, London WC2, UK.*

More than 30 patients have been treated thus far for malignant disease by systemic or direct i.p. injections of $^{131}$I-labelled monoclonal antibodies (range 30–150 mCi $^{131}$I, specific activity ~10 mCi $mg^{-1}$ murine immunoglobulin). In each case the human anti-mouse response, the total radioiodine content of peripheral blood and total body radiation were measured. Patients receiving two or more treatments have shown a markedly more rapid clearance of radiolabel and this has been accompanied by an elevated anti-murine serum immunoglobulin. The formation of immune complexes is suggested as an explanation for this phenomenon since it is well established that they are cleared more rapidly than uncomplexed soluble antigen. The observed change in kinetics associated with second and subsequent treatments has implications with regard to the dosimetry and effectiveness of a regime requiring more than one treatment.

The organisers wish to acknowledge the support of the following organisations: CIS/UK International, Hybritech Incorporated, E.I. Dupont De Nemours, Sorin Biomedica, Behringwerke, Unipath, Pharmacia, Imperial Chemical Industries, Serotec, Lederle & the Imperial Cancer Research Fund.